# Complete Plastid Genome of the Recent Holoparasite *Lathraea squamaria* Reveals Earliest Stages of Plastome Reduction in Orobanchaceae

**DOI:** 10.1371/journal.pone.0150718

**Published:** 2016-03-02

**Authors:** Tahir H. Samigullin, Maria D. Logacheva, Aleksey A. Penin, Carmen M. Vallejo-Roman

**Affiliations:** 1 A. N. Belozersky Institute of Physico-Chemical Biology, Lomonosov Moscow State University, Moscow, Russia; 2 Pirogov Russian National Research Medical University, Moscow, Russia; 3 Department of Genetics, Faculty of Biology, Lomonosov Moscow State University, Moscow, Russia; University of Lausanne, SWITZERLAND

## Abstract

Plants from the family Orobanchaceae are widely used as a model to study different aspects of parasitic lifestyle including host–parasite interactions and physiological and genomic adaptations. Among the latter, the most prominent are those that occurred due to the loss of photosynthesis; they include the reduction of the photosynthesis-related gene set in both nuclear and plastid genomes. In Orobanchaceae, the transition to non-photosynthetic lifestyle occurred several times independently, but only one lineage has been in the focus of evolutionary studies. These studies included analysis of plastid genomes and transcriptomes and allowed the inference of patterns and mechanisms of genome reduction that are thought to be general for parasitic plants. Here we report the plastid genome of *Lathraea squamaria*, a holoparasitic plant from Orobanchaceae, clade Rhinantheae. We found that in this plant the degree of plastome reduction is the least among non-photosynthetic plants. Like other parasites, *Lathraea* possess a plastome with elevated absolute rate of nucleotide substitution. The only gene lost is *petL*, all other genes typical for the plastid genome are present, but some of them–those encoding photosystem components (22 genes), cytochrome b_6_/f complex proteins (4 genes), plastid-encoded RNA polymerase subunits (2 genes), ribosomal proteins (2 genes), *ccsA* and *cemA*–are pseudogenized. Genes for cytochrome b_6_/f complex and photosystems I and II that do not carry nonsense or frameshift mutations have an increased ratio of non-synonymous to synonymous substitution rates, indicating the relaxation of purifying selection. Our divergence time estimates showed that transition to holoparasitism in *Lathraea* lineage occurred relatively recently, whereas the holoparasitic lineage Orobancheae is about two times older.

## Introduction

Photosynthesis has been lost independently numerous times in flowering plant lineages during the transition to a parasitic lifestyle. By recent estimates, approximately 4500 species (~1%) of flowering plants are parasitic [1]. About 80% of these are hemiparasites, i.e. photosynthetically competent plants with various efficiencies of photosynthesis; some parasites can even survive without a host (facultative hemiparasites), though usually they are associated with the host. The largest parasitic family is Orobanchaceae; it consists of ca. 2047 species of 89 genera [2] and includes both a single autotrophic lineage, the genus *Lindenbergia* Lehm., and all types of parasitism: facultative, obligate hemiparasites, and holoparasites (plants that completely lack photosynthetic activity) [3]. The results of phylogenetic studies indicate that there are at least three (up to five) origins of holoparasitism in Orobanchaceae, one on the branch to *Orobancheae* (~180 species), another on the branch to *Lathraea* (5–7 species), the third on the branch to *Hyobanche* (*Harveya*) (~70 species) [4, 5, 6, 7].

There are sixteen Orobanchaceae parasitic species for which complete sequences of the plastid genome are now available, including *Epifagus virginiana* (L.) W.P.C.Barton, the first parasitic plant with a fully sequenced plastome [8], the hemiparasite *Schwalbea americana L*., *and* holoparasites *Boulardia latisquama* F.W.Schultz, *Cistanche phelypaea* Cout., *Conopholis americana* (L.) Wallr., *Myzorrhiza californica* (Cham. & Schltdl.) Rydb., *Orobanche crenata* Forssk., *Orobanche gracilis* Sm., *Phelipanche purpurea* (Jacq.) Soják, *Phelipanche ramosa* (L.) Pomel [9], *Cistanche deserticola* Ma [10], *Orobanche austrohispanica* M.J.Y.Foley, *Orobanche densiflora* Salzm. Ex. Reut., *Orobanche rapum-genistae* Thuill., *Orobanche cumana* Wallr., *Orobanche pancicii* Beck [11]. All of the holoparasites represent the Orobancheae branch of loss of photosynthesis, thus processes of plastome modification in this clade are well characterized. The plastomes of holoparasites exhibit various degrees of reduction of both gene content and size due to the release of selective constraints after the switchover to parasitic lifestyle [9]. The reduction appears to be a common feature of plastid genome evolution of heterotrophic plants (obtaining nutrients from other plants or from fungi) and to follow a certain path generalized in a model of plastid genome degradation proposed by Barrett and Davis [12, 13]. Increased rate of nucleotide substitution seems to be another general feature that characterizes the nuclear, mitochondrial, and plastid genomes of parasitic plants [14, 15]. Faster rate of plastome nucleotide substitutions was also reported in another group of heterotrophic plants, mycoheterotrophs [16, 17] (but see [18]), and in some photosynthetic plants; in this latter case it is usually correlated with highly rearranged plastid genome [19, 20].

In this study, we report the complete plastid genome of *Lathraea squamaria* L. (common toothwort)—a non-photosynthetic plant widespread in Europe; it parasitizes on the roots of hazel, alder and other trees. *L*. *squamaria* represents the second branch within the Orobanchaceae family where loss of photosynthesis occurred—the Rhinantheae clade (sensu McNeal *et al*. [5]). In contrast to the Orobancheae clade, where most representatives are non-photosynthetic, the genus *Lathraea* is the only holoparasitic one in this clade. The plastomes of the Orobancheae clade have been extensively studied, allowing inference of patterns and mechanisms of genome reduction that are thought to be general for parasitic plants [9, 11]. Characterization of the *L*. *squamaria* plastome will reveal if these patterns are conserved in other clades. A partial sequence of the plastid genome of its close relative, the facultative hemiparasite *Bartsia inaequalis Benth*., was recently published [21], and thus provides a basis for comparative analysis.

## Materials and Methods

### Genome sequencing, assembly, and annotation

Total DNA was extracted using the CTAB method [22] from a single plant growing in the wild in Vorobyevy Gory Park (Moscow, Russia). *L*. *squamaria* is not an endangered or protected species and no specific permissions were required for collection. A voucher specimen was deposited at the Herbarium of Moscow State University, Russia (MW, M.D.Logacheva 16). One microgram of DNA was sheared by sonication using a Covaris S220 instrument (Covaris Inc., USA). Further steps of library preparation were performed using a TruSeq DNA Sample Prep Kit (Illumina, USA) according to the manufacturer’s instructions. The library was sequenced using an Illumina HiSeq 2000 instrument with read length 101 bp from each end of the fragment. Resulting reads were processed using the CLC Genomics Workbench v. 5.5 software. The processing included trimming of low quality (q<30) nucleotides and adapters and assembly with the following parameters: bubble size 50 nt, word size 21 nt (automatic choice), minimal contig length 1000 nt, no scaffolding. Among the resulting contigs, we selected those that have high similarity to plastid genomes but do not contain any sequences with similarity to mitochondrial genomes. These contigs were aligned to the *Lindenbergia* plastome; since no rearrangements relative to *Lindenbergia* were found within contigs, we assumed that gene order in *Lathraea* is not changed in general. Based on this assumption, we ordered the contigs and designed primers that match the ends of the contigs for joining them using PCR (the list of primers is available in S1 Table). PCR products were sequenced using Sanger technology (short amplicons, less than 1000 bp) or using Illumina sequencing (longer amplicons). The boundaries of the inverted repeats and single copy regions as well as the lack of *petL* in the *psbE-petG* region were verified with PCR followed by Sanger sequencing and by mapping of trimmed read pairs to the assembly and to *Lindenbergia petL* sequence using the Novoalign V.3.02.10 software (www.novocraft.com). The assembled plastid genome sequence was annotated using the online tool CpGAVAS [23] with default options and checked manually.

### Gene expression and editing

To check the presence of spliced transcripts and/or stop-codon RNA editing of several plastid genes (*rpoC1*, *rpoC2*, *rbcL*), we extracted RNA and obtained cDNA of *Lathraea*. RNA was extracted from two parts–immature fruits and perianth. The cDNA was synthesized with a ThermoScript RT-PCR System for first-strand cDNA synthesis (Invitrogen, USA) with random hexamers used as primers. Then partial cDNA was amplified using gene-specific primers (S1 Table), and the PCR products were cleaned and sequenced using the same primers.

### Genome analyses

Phylogenetic analysis was performed using a set of sequences of two rRNA and 35 protein-coding genes from 14 Orobanchaceae plastid genomes (*Lindenbergia*, NC_022859; *Schwalbea*, NC_023115; *Boulardia*, NC_025641; *Cistanche phelypaea*, NC_025642; *Cistanche deserticola*, NC_021111; *Conopholis*, NC_023131; *Epifagus*, NC_001568; *Myzorrhiza*, NC_025651; *Orobanche crenata*, NC_024845; *Orobanche gracilis*, NC_023464; *Phelipanche purpurea*, NC_023132; *Phelipanche ramosa*, NC_023465; *Bartsia*, KF922718 and *Lathraea*, NC_027838) and three genomes as an outgroup (*Tectona*, NC_020098; *Salvia*, NC_020431; and *Solanum lycopesicum*, NC_007898). We considered only plastid genes present both in *Lathraea* and in other plants. Nucleotide sequences were aligned according to the corresponding amino acid alignment produced by MUSCLE [24], and frameshift mutations were corrected manually. The most variable and gap-rich positions were excluded from the alignment using the GBLOCKS program [25], the “softest” settings being used. We tested for saturation of substitutions using DAMBE [26], and the analysis showed no substantial saturation (Iss<Iss.c; *P*<0.0001) [27]. Phylogenetic trees were reconstructed using the maximum likelihood approach as implemented in RAxML [28]. The GTR+Γ model was used, and the best partition scheme was selected in the PartitionFinder program [29]. ML branch support was assessed via 100 nonparametric bootstrap pseudoreplicates using the “rapid” bootstrap approach. The obtained topology was used in the divergence time estimation with the Bayesian approach as implemented in MCMCtree of PAML 4.8 [30]. We used the GTR+Γ model for nucleotide substitution and the independent rates model for the relaxed molecular clock, which was selected after comparison of marginal likelihoods estimated with the stepping stone sampling (-log likelihoods were 197,259, 198,531, and 202,465 for independent rates, correlated rates, and strict clock, respectively). Three time calibration points obtained by Wikström *et al*. [31] − the Lamiales–Solanales split at 95–109 million years ago (MYA), the Lamiaceae–Orobanchaceae split at 44–60 MYA, and the Orobanchaceae crown group age at 13–38 million years (MY) − were applied to corresponding nodes as age constraints. To ascertain whether convergence was achieved, three independent MCMC analyses were performed, each with 2×10^6^ steps, the first 2×10^5^ steps being discarded as burn-in. Absolute substitution rate was calculated by dividing the ML tree branch length by the age of the branch.

The codon-based Z-test of neutrality [32] and Tajima’s relative rate test [33] were performed in the MEGA 6 program [34] using a reduced five-species set (*Lathraea*, *Bartsia*, *Lindenbergia*, *Tectona*, *Salvia*). Relative rates in *Lathraea* and *Bartsia* versus *Lindenbergia* with *Tectona* as an outgroup were compared, as well as *Lathraea* versus *Bartsia* with *Lindenbergia* as an outgroup. Comparisons were performed using all concatenated data, as well as separately in groups of genes combined according to their function (ribosomal RNA genes, *psa+psb+pet* genes, *rpo* genes, *atp* genes, *rpl+rps* genes), and genes *accD*, *clpP*, *matK*, *ycf1*, and *ycf2* were analyzed individually.

The proportion of synonymous and nonsynonymous substitutions per synonymous and nonsynonymous site (*d*_*S*_ and *d*_*N*_, respectively) and the ratio ω = *d*_*N*_ /*d*_*S*_ were assessed with the maximum likelihood approach as implemented in the CODEML program of the PAML package, “branch” models with the F3x4 codon frequency model being used. Evaluation of ω difference in parasitic and photosynthetic plant lineages was performed using the five-species set because of reduction of gene content in Orobancheae plastid genomes. Four hypotheses of different *d*_*N*_ /*d*_*S*_ ratios for *Lathraea* versus photosynthetic plants + *Bartsia* and *Bartsia* versus photosynthetic plants + *Lathraea* were tested. Branch models corresponding to the hypotheses were a “one-ratio” model that assumes that all the branches have the same ω-values (model M1), a “three-ratio” model that assumes different ω-values for photosynthetic, hemiparasitic, and holoparasitic branches (model M3), a “two-ratio” model in which the hemiparasitic branch (*Bartsia*) is allowed to have a separate ω-value (model M2B), and another “two-ratio” model in which the same is allowed in the holoparasitic branch (*Lathraea*, model M2L). Likelihood ratio tests (LRT) M1 versus M2B and M2L versus M3 examined whether the ω-ratio for the *Bartsia* branch is different from the background ratio, and LRT M1 versus M2L and M2B versus M3 examined the *Lathraea* branch. Twice log-likelihood difference between models was tested against the χ^2^ distribution with one degree of freedom, and *P* values for multiple comparisons were Bonferroni corrected: the corrected significance level was calculated as α divided by the number of hypotheses being tested.

Evidence for positive selection acting on the *accD*, *clpP*, *matK*, *ycf1*, *ycf2* and *atp* genes was tested using the full set of taxa (except for those that were lacking the corresponding gene) with the CODEML program in the PAML package using “branch-site” models, which allow ω to vary both among sites in the sequence and across pre-specified branches or clades on the tree. We used a model A and performed test 2 as described in Zhang *et al*. [35]. When *atp* genes were used, both terminal and internal branches within the parasitic subtree of Orobanchaceae were tested with Bonferroni correction for multiple comparisons. The same test was applied solely to the *Lathraea* branch using *accD*, *clpP*, *matK*, *ycf1*, and *ycf2* sequences.

## Results

As a result of sequencing, 7,079,624 paired end reads were obtained and assembled into 293 contigs. Among them, we revealed eight contigs of length more than 5,000 nucleotides with high similarity to the plastid genome of *Lindenbergia*. The coverage of two of them, 251X and 293X, suggesting that they correspond to repeat region, and the coverage of the remaining six was 130X to 143X. The complete plastid genome of *L*. *squamaria* is a circular molecule 150,504 bp in length and possesses a typical architecture with a large single-copy (LSC) region of 81,981 bp separated from the 16,061 bp small single-copy (SSC) region by two inverted repeats (IRs), each of 26,231 bp. The GC content is heavily biased (38.13%) (Table 1).

**Table 1:** Characteristics of *Lathraea squamaria* plastid genome.

Genome length (bp)	150,504
LSC length (bp)	81,981
SSC length (bp)	16,061
IR length (bp)	26,231
Number of different genes	78
Number of different protein-coding genes	46
Number of different tRNA genes (duplicated in IR)	30 (7)
Number of different rRNA genes (duplicated in IR)	4 (4)
Number of genes duplicated in IR	14
Number of different genes with introns	12
Number of pseudogenes	32
GC content (%)	38.13

**Fig 1:**
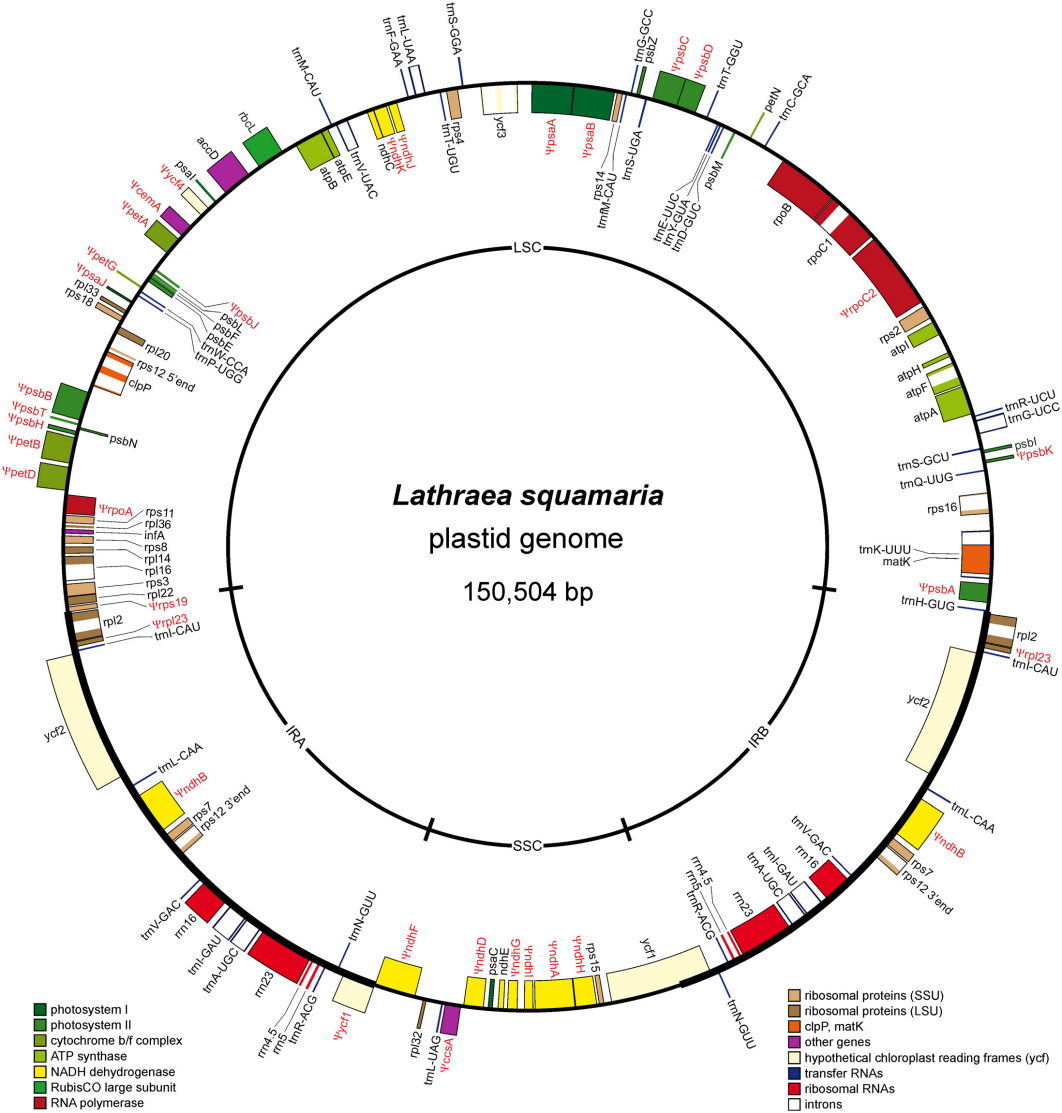
Gene map of the plastid chromosome of *Lathraea squamaria*. Genes shown inside the circle are transcribed clockwise; those outside the circle are transcribed counterclockwise. The large single copy region (LSC) and the small single copy region (SSC) are separated by two inverted repeats (IRa and IRb). Pseudogenes are marked by Ψ.

The annotated plastome was deposited in GenBank (accession number NC_027838). The gene map for the plastome of *L*. *squamaria* is shown in Fig 1.

Among the 78 unique genes present in *L*. *squamaria*, a set of 46 protein-coding genes, 30 tRNA genes, and four rRNA genes were identified (S2 Table). No major rearrangements were observed based on alignment to the autotrophic *Lindenbergia* plastome. The *petL* gene was completely lost (no reads were mapped to *petL* of *Lindenbergia*), and 32 putative pseudogenes (reading frame interrupted by internal stop codons or insertion/deletion events) were detected among the protein coding genes in the *Lathraea* plastome. Nine plastid genes encoding subunits of the NAD(P)H dehydrogenase complex (*ndh* genes) became pseudogenes, and *ndhC* and *ndhE* ORF persisted. *Three genes of* photosystem I (*psaA*, *psaB*, *psaJ*), eight of the photosystem II subunit (*psbA*, *psbB*, *psbC*, *psbD*, *psbH*, *psbJ*, *psbK*, *psbT*), four members of the cytochrome b_6_/f complex (*petA*, *petB*, *petD*, *petG*), and two genes for ribosomal proteins (*rps19*, *rpl23*) were detected as pseudogenes. Two of four genes of the plastid-encoded RNA polymerase (PEP), *rpoA* and *rpoC2*, were also found to be pseudogenes due to multiple frameshifts in the former and an internal stop codon in the latter. The sequence of the RUBISCO large subunit gene *rbcL* contains an internal stop codon. *Lathraea* also has pseudogenes for cytochrome C biogenesis protein *ccsA*, envelope membrane protein *cemA*, and the putative photosystem I protein *ycf4*. No pseudogenes were detected in encoding subunits of the ATP synthase complex (*atp* genes).

Visual inspection of the mapped reads recovered multiple single nucleotide polymorphisms and indels, which were unevenly scattered over the plastome with *rpoC1*, *rpoB*, *psaA*, *psaB*, *cemA*, *ycf2*, *ndhB*, and *ndhG* being the most abundant. Interestingly, within the *rbcL* sequence one of three single nucleotide variants affected the premature stop codon so that a CGA triplet was restored whereas others did not change amino acids but always restored conserved codons (S1 Appendix), thus at least two different *rbcL* copies coexist in *Lathraea*. A second copy location is currently unknown: it may either be transferred to the mitochondrial genome or represent plastid heteroplasmy. Sequencing of the *rbcL* cDNA showed that both the pseudogene (major variant) and the putatively functional (minor) sequence were transcribed in a proportion similar to a major-to-minor DNA variants ratio suggesting no RNA editing in the *Lathraea rbcL* sequence (S1 Fig). *rpoC2* cDNA sequencing also showed no stop codon editing and confirmed *rpoC2* pseudogenization. It should be noted also, that the *rpoC1* spliced intron was found in fruits, but no intron processing was detectable in perianth.

**Fig 2:**
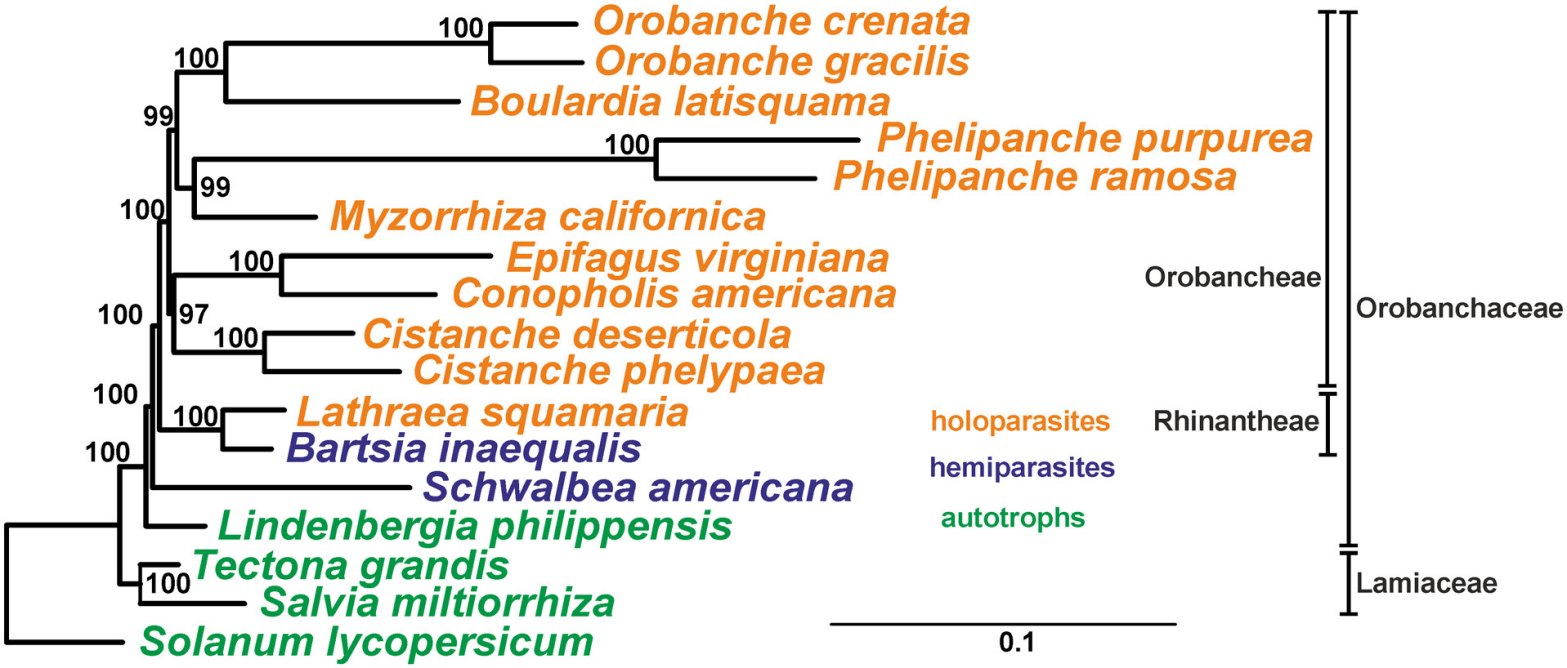
Phylogenetic tree of the 17 taxa inferred by the maximum likelihood approach. Bootstrap support values are provided at the nodes. The scale bar corresponds to 0.1 substitution per site.

Phylogenetic analyses of the coding genes yielded a completely resolved Orobanchaceae tree (Fig 2) with the autotrophic genus *Lindenbergia* as a basal branch with bootstrap support (BS) 100%.

**Fig 3:**
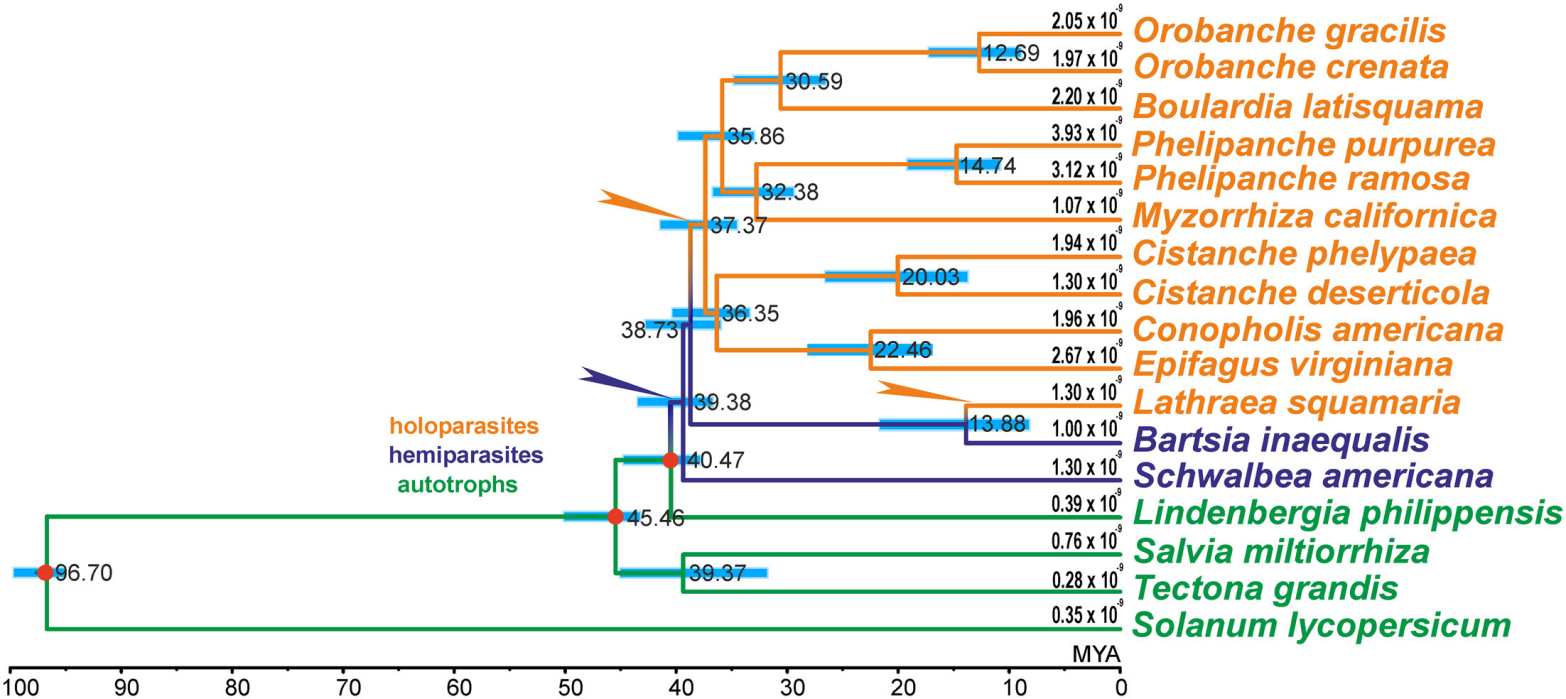
Estimates of divergence time of 17 taxa on the ML tree. The values at the nodes represent mean ages in million years, and the 95% highest posterior density (HPD) interval is indicated by a blue bar. At terminal branches, substitution rates (per site per year) are provided. Nodes where parasitism and holoparasitism evolved are indicated by blue and yellow arrows, respectively. Age constraints were applied to nodes marked by red dots.

Hemiparasitic *Schwalbea* is a sister to the remaining broomrape clades (BS 100%). The next split within the plastid tree occurs between the *Bartsia* and *Lathraea* clades on one hand and the clade including the other holoparasitic genera (BS 100%) on the other. The latter clade groups *Cistanche* species with *Conopholis* and *Epifagus*, as well as *Phelipanche* species and *Myzorrhiza* with *Orobanche* species and *Boulardia* (BS 97%-100%). The estimated time of split of *Bartsia* and *Lathraea* lineages was 13.88 MYA (95% HPD 8.17–21.59 MY, S2 Fig), which gives absolute substitution rates 1×10^−9^ and 1.3×10^−9^ substitutions per site per year in *Bartsia* and *Lathraea* lineages, respectively, whereas in sampled autotrophic lineages the substitution rate does not exceed 0.76×10^−9^ (Fig 3).

The length of a *Lathraea* branch on the phylogram is comparable with the branches of *Bartsia*, *Tectona*, or *Lindenbergia*. The fact that *Lindenbergia* is at least two times older than *Lathraea* and *Bartsia* implies considerable difference in their sequence evolution rates. The genome-wide relative rate test confirms that the rate difference is statistically significant (at α = 0.05) in all comparisons. However, the substitution rate acceleration is not uniform across compared genomes and functional groups of genes. Thus, there is no significant rate difference in rRNA sequences, but other groups of genes in *Lathraea* and *Bartsia* evolve faster than in photosynthetic plants. Relative rate tests show also insignificant rate difference in *rpo*, *atp*, and *rpl/rps* sequences between *Bartsia* and *Lathraea*, while *psa/psb/pet* genes and a group of miscellaneous genes (*accD/clpP/matK/ycf1/ycf2*) in *Lathraea* evolve faster than in *Bartsia*. In the latter case the difference is due to the *matK* gene, because in *accD*, *ycf1*, and *ycf2* the rate difference is not significant (*clpP* from *Bartsia* is not available) when the genes are tested separately.

Ratio ω (*d*_*N*_ /*d*_*S*_) can be considered uniform for all groups of genes when hemiparasitic *Bartsia* is pre-specified as a foreground branch: ω-value differences are not significant, and the null hypotheses (neither “one-ratio” nor “two-ratio” models) cannot be rejected at 1.25% level. The same is true for the *rpo*, *atp*, and *rpl/rps* genes when *Lathraea* is a branch of interest. However, for photosynthesis-related genes *psa/psb/pet*, models M2L and M3 fit the data significantly better than M1 and M2B (*P*<0.0001), suggesting individual ω-value for a holoparasitic lineage (*Lathraea*) being more than 7× that of other taxa (0.286 and 0.04, respectively). The LRT also indicates a better fit of a “two-ratio” model (models M3 and M2B were not tested) for the *clpP* gene (*P*<0.0001) with the ω estimate for *Lathraea* being equal to 1, i.e. reaching neutrality (ω = 0.05 for background branches). For *accD*, *matK*, *ycf1*, or *ycf2*, none of the tested branch models has a significantly better fit.

Ratios ω (*d*_*N*_ /*d*_*S*_) estimated using the “three-ratio” model M3 did not exceed 1 and exceeded 0.5 (close to neutral evolution) for a few fast evolving genes (*clpP*, *matK*, *ycf1*, *ycf2*). The codon-based Z-test rejected the null hypothesis of neutrality in favor of negative selection in pairwise comparisons for individually analyzed genes and all groups except for the *clpP* gene. The branch-site test for evidence of positive selection acting on *atp* genes was applied to all eleven branches of the parasitic subtree of Orobanchaceae. The likelihood ratio statistic was non-zero for the *Lathraea* (1.126) and *Myzorrhiza* (3.99) branches, but in no case was the LRT statistic significant after Bonferroni correction (*P* = 0.144 and *P* = 0.023 for *Lathraea* and *Myzorrhiza*, respectively) at α = 0.0045. Among other genes (*accD*, *clpP*, *matK*, *ycf1*, *ycf2*) tested with *Lathraea* lineage specified as a foreground branch, only *clpP* displayed statistically significant positive selection (*P* = 0.001) at α = 0.05, but no particular codons with high posterior probability (higher than 0.95) of being under positive selection were identified.

## Discussion

The newly determined sequence for the *L*. *squamaria* plastid genome displays a typical quadripartite architecture with the large single-copy and small single-copy regions separated by two inverted repeats. The plastome is the longest one among known holoparasites of the Orobanchaceae family [9, 11], and it has not suffered structural rearrangements. However, relaxed selective constraint for the function of photosynthesis resulted in extensive pseudogenization of the plastome.

The result of the phylogenetic analysis (Fig 2) is consistent with results of phylogenetic analyses of all plastid ribosomal protein genes [9] and with previous studies with multilocus data sets based on the three nuclear (ITS, *PHYA*, *PHYB*) and two plastid (*matK*, *rps2*) markers [5] except for the placement of *Cistanche*: in McNeal *et al*. [5] *Cistanche* species are closer to the *Orobanche*/*Phelipanche* clade than to *Conopholis*/*Epifagus*. In our tree parasitic species form a monophyletic group whereas holoparasites do not, and it is very unlikely that hemiparasitic *Bartsia* and holoparasitic *Lathraea* lineages could have a holoparasite as a common ancestor. Therefore, the topology obtained does not contradict a single transition to a parasitic lifestyle and independent transitions to obligate parasitism in Orobanchaceae as it was stated earlier in [6, 36, 4].

**Fig 4:**
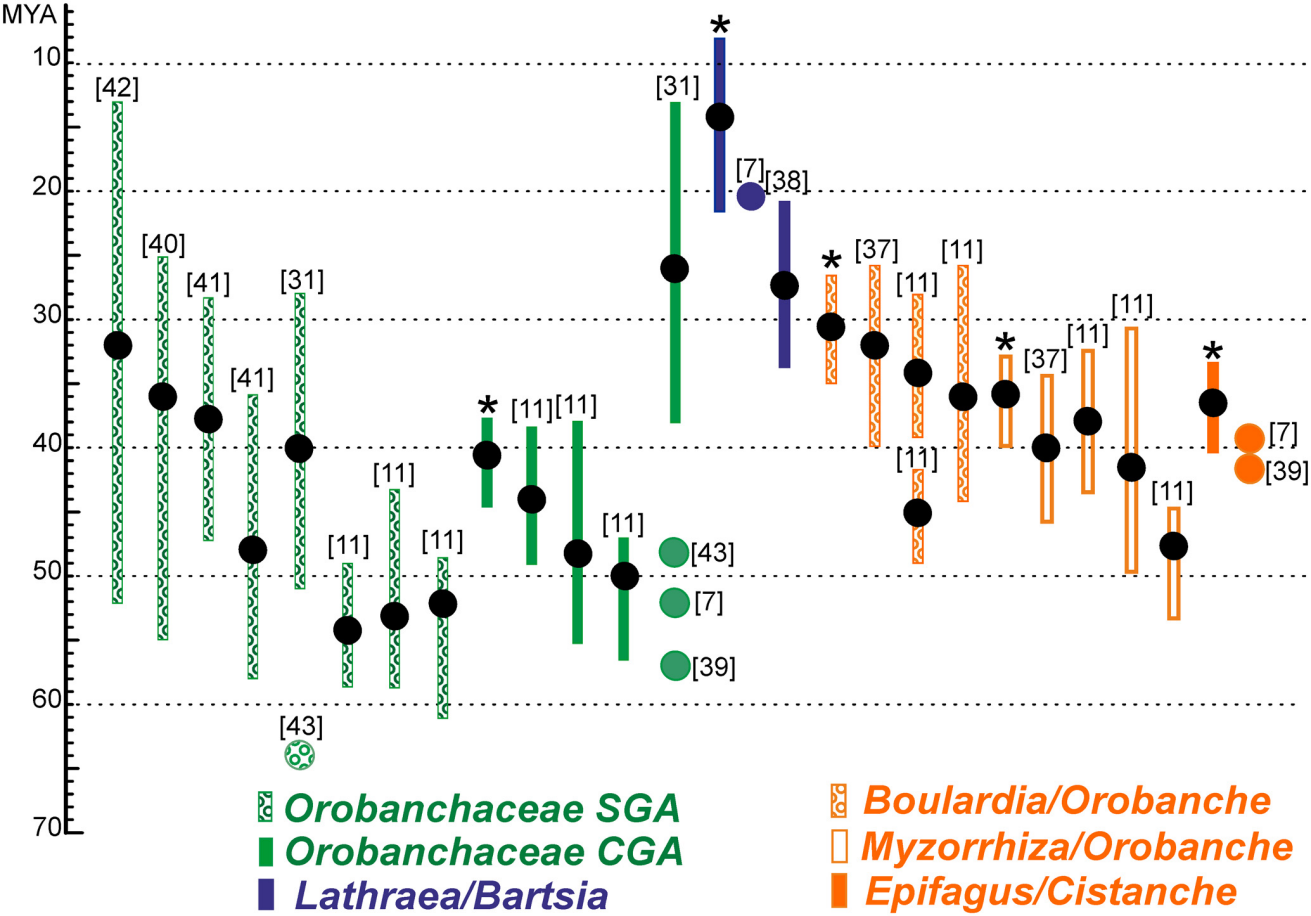
Comparison of estimated here divergence ages for four nodes and the Orobanchaceae crown group age (CGA) with reported ages from other studies and with the Orobanchaceae stem group age (SGA). Mean and 95% HPD interval of each estimation (when provided) is shown with a reference given in brackets. Asterisks mean this study.

Our divergence time estimates (Fig 3, S2 Fig) show that this transition in *Lathraea* lineage occurred relatively recently, whereas the holoparasitic lineage Orobancheae is about two times older, and support recent findings that parasitism in Orobanchaceae originated within 5 MY [11]. Age estimates for some nodes are compared to those reported earlier in other studies in Fig 4.

The age estimates of nodes *Myzorrhiza*–*Phelipanche* (32.78 MY, 95% HPD 29.33–36.84 MY), *Boulardia*–*Orobanche* (30.59 MY, 95% HPD 26.45–34.90 MY) and *Myzorrhiza*–*Orobanche* (35.85 MY, 95% HPD 32.85–39.90 MY) are close to those calculated with a clock calibrated with an ITS substitution rate taken from *Gentianella* (Gentianaceae) [37] and with the results of recent age-constrained dating analyses of Orobanchaceae plastid genomes [11], while ages of some other nodes differ substantially from those obtained earlier. Thus, based on nuclear ITS molecular clock, estimate of the absolute timing of the diversification of lineages within Orobanchaceae resulted in divergence time of a *Lathraea*–*Bartsia* split at 21 MYA (no HPD interval provided) [7], which is marginal in our HPD interval of 8.17–21.59 MY (mean 13.88 MY). Recently, Uribe-Convers and Tank used geological data in addition to secondary calibration points from Wolfe *et al*. [7] and deduced the age of the same point at 27.38 MY (95% HPD 20.87–33.57 MY) from plastid *trnT–trnL* intergenic spacer and the nuclear ITS region [38]. Focusing on basal eudicots and using 79 chloroplast protein-coding genes Wu *et al*. [39] revealed the *Epifagus*–*Cistanche* split age of 42 MY, the same node was calculated at 39.4 MY by Wolfe *et al*. [7], while a younger age of 36.35 MY (95% HPD 33.29–40.43 MY) is obtained here. For the split of *Conopholis* and *Epifagus*, an age of 7.8 MY was estimated by Wolfe *et al* [7] which is here estimated at 22.46 MY (95% HPD 17–28 MY). We deduced the Orobanchaceae crown group age of 40.47 MY (95% HPD 37.67–44.8 MY), which is consistent with analyses using similar age-constraints [11], but it corresponds rather to recent estimates of the Orobanchaceae stem group age (40 MY, 95% HPD 28–51 MY in Wikström *et al*. [31]; 35.91 MY, 95% HPD 25.38–55.01 MY in Magallón *et al*. [40]; 38 MY, 95% HPD 28–47 MY in Bell *et al*. [41], 32 MY, 95% HPD 13–52 MY in Naumann *et al*. [42]; but the crown group age of 57 MY was provided in Wu *et al*. [39], 52 MY in Wolfe *et al*. [7], 48 MY and the stem group age of 64 MY in Bremer *et al*. [43]), thus ages obtained here may be overestimated to some extent. Limited with available broomrape plastid genomes, our dating approach suffers from undersampling of Orobanchaceae; nevertheless, even being only rough approximations, our results reveal relative ages of broomrape holoparasites and clearly demonstrate absolute rate difference in *Lathraea* and photosynthetic lineages, the genome-wide rate of nucleotide substitution in plastid genome of *Lathraea* being elevated as in other parasitic plants.

Surprisingly, the overall genomic structure of the *L*. *squamaria* plastome is more similar to the plastid genome of autotrophic *Lindenbergia* than to that of the closely related holoparasite *Lathraea clandestina*. Hybridization experiments demonstrated that the plastome of *L*. *clandestina* was approximately 110-kb long and has a drastically reduced SSC region (which in land plants contains most of the *ndh* genes for subunits of a plastid NAD(P)H dehydrogenase), in addition to pseudogenization of *atpB* and *atpE* genes [44]. In the *L*. *squamaria* plastome, *atpB* and *atpE* were maintained as functional genes and none of the *ndh* genes was lost, but nine out of the eleven *ndh* genes were pseudogenized. Degradation of the *ndh* complex is not attributed solely to heterotrophs and occurred independently in many angiosperm lineages, including bryophytes [45], gymnosperms [46], orchids [47, 48, 49], Lentibulariaceae [50], Alismatales [51, 52], Santalales [53], Cactaceae [54], and Geraniaceae [55]. The lack of functional *ndh* genes is typical for parasitic Orobanchaceae ([9, 11], this study). Wicke *et al*. [9] proposed that four *ndh* genes (*ndhA*, *ndhD*, *ndhG*, *ndhF*) were pseudogenized after the transition to obligate parasitism and mapped this event to a common ancestor of *Schwalbea* and other parasites [9, 11]. However, a functional *ndhA* gene is present in facultative hemiparasite *Bartsia* [21], while *ndhD*, *ndhG* and *ndhF* are not pseudogenized in another hemiparasite *Castilleja miniata* (*Pedicularideae* clade, sensu McNeal *et al*. [5]) [38], what suggests that the *ndh* loss-of-function in *Schwalbea*, *Lathraea*, and Orobancheae lineages occurred convergently. Within mycoheterotrophic Orchidaceae, *ndh* losses have been frequent and have occured in both photosynthetic and nonphotosynthetic lineages [49], in parallel with that, one may also expect a complex pattern of independent gene loss and pseudogenization across both holo- and hemiparasites in Orobanchaceae.

The majority of subunit genes of photosystem I and II (*psa*, *psb*), as well as many members of the cytochrome b_6_/f complex (*pet*) of the *Lathraea* plastome, have been turned into pseudogenes. The remaining *psa/psb/pet* genes retained intact open-reading frames but clearly displayed signature of relaxed selective constraint: the estimated ω-ratio (*d*_*N*_ /*d*_*S*_) is approximately seven times higher than that of non-holoparasite lineages (0.286 and 0.040, respectively), and this difference is statistically significant (*P*<0.0001). Contrary to *Lathraea*, the *psa/psb/pet* genes in *Bartsia* evolved under the same negative selection constraints as in the photosynthetic *Lindenbergia* and outgroups.

Since in the *Lathraea* plastid genome only two of four plastid-encoded RNA-polymerase (PEP) genes (*rpoB* and *rpoC1*) have intact reading frames, the PEP seems to be nonfunctional, similarly to *L*. *clandestina* [56]. It is believed that the PEP transcribed by the nuclear-encoded RNA-polymerase (NEP) is responsible for transcription of photosynthesis-related genes, so this function in *Lathraea* may be rendered redundant. The *rpo* genes themselves are transcribed, and at least two of them, *rpoC1* and *rpoC2*, were amplified by RT-PCR and did not contain detectable RNA-editing sites. It is interesting to note a tissue-specificity of processing of the *rpoC1* intron: spliced intron was found in fruits, but no intron processing was detectable in perianth (S1 Fig), it still being not quite clear why such specificity exists. In *Arabidopsis rpoC1* intron is spliced by the nuclear encoded CRS2–CAF1 complex [57], so the cause will be found most probably outside of the plastid genome.

As neither ribosomal RNA genes nor transfer RNAs are lost or pseudogenized in the *Lathraea* plastome, all basic housekeeping functions seem to be preserved despite pseudogenization of two ribosomal protein genes, *rps19* and *rpl23*. In the plastomes of eudicots, *rpl23*, as well as some other ribosomal protein genes, have been independently lost in several lineages [58] with no harm to the translation apparatus due to import of missing elements [59].

Not being associated with housekeeping processes, a set of ATP synthase complex genes (*atp*) is also putatively functional. A similar situation has been reported for other non-photosynthetic plants–holoparasites [60, 9] and mycoheterotrophs [45, 13, 18]. Recently, an ATP hydrolysis function in assistance to the Twin-arginine translocator (Tat) system was suggested for non-photosynthetic plastid ATP synthase genes [61]. The Tat-related role may explain the retention of the intact *atp* complex genes in the *Lathraea* plastome after the loss of photosynthesis.

In non-green mycoheterotrophic *Corallorhiza* species the *atp* complex displayed evidence for positive selection [13], but our test showed no signature of divergent selection acting on *atp* genes in any of branches of the parasitic subtree of Orobanchaceae. Our inference of positive selection in fast-evolving gene *clpP* needs to be confirmed by further investigation with broader sampling of Orobanchaceae representatives. In the last decade, many studies of DNA variation patterns have inferred positive selection at different plastid sequences, including photosynthesis related, PEP, *matK*, *clpP*, *ycf1*, and *ycf2* genes (reviewed in Bock *et al*. [62]). However, without experimental evidence, drivers and evolutionary significance of the revealed examples of adaptive organelle evolution remain obscure.

Lastly, our mapping of the paired reads to the plastome assembly revealed the high level of nucleotide polymorphism, and polymorphic sites were recovered both in pseudogenes and coding sequences. Definitely, some of single nucleotide variants and indels are of mitochondrial origin, since the transfer of plastid DNA into the mitochondrial genome is a well-known phenomenon [63] and almost all of plastid genes have been shown in various plants transferred to the mitochondria (e.g., [64, 65]. Most of the transferred protein-coding sequences have no intact gene structure or have frameshifts/indels but still may be transcribed: transcription of non-plastid *ndh* sequences (including pseudogenes) was recently shown in Orchidaceae [66]. On the other hand, we cannot exclude presence of plastid heteroplasmy in *Lathraea*. In mycoheterotrophic *Neottia nidus-avis* at least three distinct *rbcL* sequences were found as pseudogenes and were likely located in the plastid genome [67]. Among broomrape species, *Hyobanche glabrata* was shown to have five different *rbcL* transcripts and all also encoded pseudogenes [68]. In recent years, growing data on plant mitochondrial genomes have made possible more thorough evaluation of plastome heteroplasmy which appeared to be more common than thought previously (discussed in Sabir *et al*. [69]). We expect that the characterization of the *Lathraea* complete mitochondrial genome sequence will help to distinguish between the possible causes of the observed polymorphism.

## Supporting Information

S1 Appendix Alignments of *Lathraea squamaria* minor sequences and pseudogenes to orthologs of photosynthetic plants. (PDF)

S1 Fig Analysis of *rpoC1*, *rpoC2*, and *rbcL* cDNAs. (PDF)

S2 Fig Estimated mean ages and 95% highest posterior density intervals. (PDF)

S1 Table Primers used for plastome assembly, verification, and cDNA amplification. (PDF)

S2 Table List of genes present in the plastid genome of *Lathraea squamaria* and *Lindenbergia philippensis*. (PDF)
