# Supplementary material for: Complete Plastid Genome of the Recent Holoparasite Lathraea squamaria Reveals Earliest Stages of Plastome Reduction in Orobanchaceae
Source: PLoS One. 2016 Mar 2;11(3):e0150718. doi: 10.1371/journal.pone.0150718 (PMC4775063; doi:10.1371/journal.pone.0150718)
Supplement: S1 Appendix — (PDF) [file pone.0150718.s001.pdf]

S1 Appendix. Alignment of some plastid sequences and minor sequences of *Lathraea*.

rbcL*, *ndhA*, *ndhJ*, *ndhB*, *psaA*, *psaB* and *ycf2

rbcL

```
                                   10        20        30        40        50        60        70        80        90       100       110       120
                          ....|....|....|....|....|....|....|....|....|....|....|....|....|....|....|....|....|....|....|....|....|....|....|....|
L.squamaria minor variant ATGTCACCACAAACAGAGACTAAAGCAAGTGTTGGATTCAAAGCGGgTGTTAAAGAGTACAAATTGACTTATTATACTCCTGAATACGAAACAAAAGATACTGATATCTTGGCAGCATTC
                           M  S  P  Q  T  E  T  K  A  S  V  G  F  K  A  G  V  K  E  Y  K  L  T  Y  Y  T  P  E  Y  E  T  K  D  T  D  I  L  A  A  F 
Lathraea squamaria        ATGTCACCACAAACAGAGACTAAAGCAAGTGTTGGATTCAAAGCGGATGTTAAAGAGTACAAATTGACTTATTATACTCCTGAATACGAAACAAAAGATACTGATATCTTGGCAGCATTC
                           M  S  P  Q  T  E  T  K  A  S  V  G  F  K  A  D  V  K  E  Y  K  L  T  Y  Y  T  P  E  Y  E  T  K  D  T  D  I  L  A  A  F 
Bartsia inaequalis        ATGTCACCACAAACAGAGACTAAAGCAAGTGTTGGATTCAAAGCGGGTGTTAAAGAGTACAAATTGACTTATTATACTCCGGAATACGAAACAAAAGATACTGATATCTTGGCAGCATTC
                           M  S  P  Q  T  E  T  K  A  S  V  G  F  K  A  G  V  K  E  Y  K  L  T  Y  Y  T  P  E  Y  E  T  K  D  T  D  I  L  A  A  F 

                                  130       140       150       160       170       180       190       200       210       220       230       240
                          ....|....|....|....|....|....|....|....|....|....|....|....|....|....|....|....|....|....|....|....|....|....|....|....|
L.squamaria minor variant cGAGTAACTCCTCAACCTGGAGTTCCGCCTGAaGAAGCaGGGGCTGCAGTCGCTGCCGAATCTTCGACTGGTACATGGACAACTGTGTGGACCGATGGACTTACcAGCCTTGATCGTTAC
                           R  V  T  P  Q  P  G  V  P  P  E  E  A  G  A  A  V  A  A  E  S  S  T  G  T  W  T  T  V  W  T  D  G  L  T  S  L  D  R  Y 
Lathraea squamaria        TGAGTAACTCCTCAACCTGGAGTTCCGCCTGAGGAAGCCGGGGCTGCAGTCGCTGCCGAATCTTCGACTGGTACATGGACAACTGTGTGGACCGATGGACTTACTAGCCTTGATCGTTAC
                           *  V  T  P  Q  P  G  V  P  P  E  E  A  G  A  A  V  A  A  E  S  S  T  G  T  W  T  T  V  W  T  D  G  L  T  S  L  D  R  Y 
Bartsia inaequalis        CGAGTAAGTCCTCAACCTGGAGTTCCGCCTGAAGAAGCAGGGGCTGCAGTCGCTGCCGAATCTTCTACTGGTACATGGACAACTGTGTGGACCGATGGACTTACCAGCCTTGATCGTTAC
                           R  V  S  P  Q  P  G  V  P  P  E  E  A  G  A  A  V  A  A  E  S  S  T  G  T  W  T  T  V  W  T  D  G  L  T  S  L  D  R  Y 

                                  250       260       270       280       290       300       310       320       330       340       350       360
                          ....|....|....|....|....|....|....|....|....|....|....|....|....|....|....|....|....|....|....|....|....|....|....|....|
L.squamaria minor variant AAAGGACGATGCTACCACATcGAGCCTGTTCCTGGAGAAACAGATCAATAtATCTGTTATGTAGCTTACCCTTTAGACCTTTTTGAAGAAGGTTCTGTTACTAACATGTTTACTTCCATT
                           K  G  R  C  Y  H  I  E  P  V  P  G  E  T  D  Q  Y  I  C  Y  V  A  Y  P  L  D  L  F  E  E  G  S  V  T  N  M  F  T  S  I 
Lathraea squamaria        AAAGGACGATGCTACCACATTGAGCCTGTTCCTGGAGAAACAGATCAATACATCTGTTATGTAGCTTACCCTTTAGACCTTTTTGAAGAAGGTTCTGTTACTAACATGTTTACTTCCATT
                           K  G  R  C  Y  H  I  E  P  V  P  G  E  T  D  Q  Y  I  C  Y  V  A  Y  P  L  D  L  F  E  E  G  S  V  T  N  M  F  T  S  I 
Bartsia inaequalis        AAAGGACGATGCTACCACATCGAGCCTGTTCCTGGAGAAACAGATCAATATATCTGTTATGTAGCTTACCCTTTAGACCTTTTTGAAGAAGGTTCTGTTACTAACATGTTTACTTCCATT
                           K  G  R  C  Y  H  I  E  P  V  P  G  E  T  D  Q  Y  I  C  Y  V  A  Y  P  L  D  L  F  E  E  G  S  V  T  N  M  F  T  S  I 

                                  370       380       390       400       410       420       430       440       450       460       470       480
                          ....|....|....|....|....|....|....|....|....|....|....|....|....|....|....|....|....|....|....|....|....|....|....|....|
L.squamaria minor variant GTAGGAAACGTATTTGGATTCAAAGCCCTGCGTGCTCTACGTCTGGAAGATCTGCGAATCcCCCCTGCTTATATTAAAACTTTCCAAGGACCGCCTCACGGGATCCAAGTTGAGAGAGAT
                           V  G  N  V  F  G  F  K  A  L  R  A  L  R  L  E  D  L  R  I  P  P  A  Y  I  K  T  F  Q  G  P  P  H  G  I  Q  V  E  R  D 
Lathraea squamaria        GTAGGAAACGTATTTGGATTCAAAGCCCTGCGTGCTCTACGTCTGGAAGATCTGCGAATCTCCCCTGCTTATATTAAAACTTTCCAAGGACCGCCTCACGGGATCCAAGTTGAGAGAGAT
                           V  G  N  V  F  G  F  K  A  L  R  A  L  R  L  E  D  L  R  I  S  P  A  Y  I  K  T  F  Q  G  P  P  H  G  I  Q  V  E  R  D 
Bartsia inaequalis        GTAGGAAACGTATTTGGATTCAAAGCCCTGCGTGCGCTACGTCTGGAAGATCTGCGAATCCCCCCTGCTTATATTAAAACTTTCCAAGGACCGCCTCACGGGATCCAAGTTGAGAGAGAT
                           V  G  N  V  F  G  F  K  A  L  R  A  L  R  L  E  D  L  R  I  P  P  A  Y  I  K  T  F  Q  G  P  P  H  G  I  Q  V  E  R  D 

                                  490       500       510       520       530       540       550       560       570       580       590       600
                          ....|....|....|....|....|....|....|....|....|....|....|....|....|....|....|....|....|....|....|....|....|....|....|....|
L.squamaria minor variant AAATTGAACAAGTATGGTCGTCCACTGTTGGGATGTACTATTAAACCTAAATTGGGGTTATCTGCTAAAAACTATGGTAGAGCAGTTTATGAATGTCTTCGCGGTGGACTTGATTTTACC
                           K  L  N  K  Y  G  R  P  L  L  G  C  T  I  K  P  K  L  G  L  S  A  K  N  Y  G  R  A  V  Y  E  C  L  R  G  G  L  D  F  T 
Lathraea squamaria        AAATTGAACAAGTATGGTCGTCCACTGTTGGGATGTACTATTAAACCTAAATTGGGGTTATCTGCTAAAAACTATGGTAGAGCAGTTTATGAATGTCTTCGCGGTGGACTTGATTTTACC
                           K  L  N  K  Y  G  R  P  L  L  G  C  T  I  K  P  K  L  G  L  S  A  K  N  Y  G  R  A  V  Y  E  C  L  R  G  G  L  D  F  T 
Bartsia inaequalis        AAATTGAACAAGTATGGTCGTCCACTGTTGGGATGTACTATTAAACCTAAATTGGGGTTATCTGCTAAAAACTATGGTAGAGCAGTTTATGAATGTCTTCGCGGTGGACTTGATTTTACC
                           K  L  N  K  Y  G  R  P  L  L  G  C  T  I  K  P  K  L  G  L  S  A  K  N  Y  G  R  A  V  Y  E  C  L  R  G  G  L  D  F  T 

                                  610       620       630       640       650       660       670       680       690       700       710       720
                          ....|....|....|....|....|....|....|....|....|....|....|....|....|....|....|....|....|....|....|....|....|....|....|....|
L.squamaria minor variant AAAGATGATGAGAACGTGAACTCCCAGCCATTTATGCGTTGGAGAGATCGTTTCTTATTTTGTGCCGAAGCAATTTATAAAGCACAGGCTGAAACAGGCGAAATAAAAGGTCATTACTTG
                           K  D  D  E  N  V  N  S  Q  P  F  M  R  W  R  D  R  F  L  F  C  A  E  A  I  Y  K  A  Q  A  E  T  G  E  I  K  G  H  Y  L 
```

```
Lathraea squamaria          AAAGATGATGAGAACGTGAACTCCCAGCCATTTATGCGTTGGAGAGATCGTTTCTTATTTTGTGCCGAAGCAATTTATAAAGCACAGGCTGAAACAGGCGAAATAAAAGGTCATTACTTG
                             K  D  D  E  N  V  N  S  Q  P  F  M  R  W  R  D  R  F  L  F  C  A  E  A  I  Y  K  A  Q  A  E  T  G  E  I  K  G  H  Y  L
Bartsia inaequalis          AAAGATGATGAGAACGTGAACTCCCAGCCATTTATGCGTTGGAGAGATCGTTTCTTATTTTGTGCCGAAGCACTTTATAAAGCACAGGCTGAAACAGGTGAAATCAAAGGTCATTACTTG
                             K  D  D  E  N  V  N  S  Q  P  F  M  R  W  R  D  R  F  L  F  C  A  E  A  L  Y  K  A  Q  A  E  T  G  E  I  K  G  H  Y  L

                            730 740 750 760 770 780 790 800 810 820 830 840
L.squamaria minor variant   AATGCTACTGCGGGTACATGCGAAGAAATGATGAAAAGAGCTGTATTTGCTAGAGAATTGGGAGTTCCTATCGTAATGCATGACTACTTAACAGGCGGATTCACTGCAAATACTAGCTTG
                             N  A  T  A  G  T  C  E  E  M  M  K  R  A  V  F  A  R  E  L  G  V  P  I  V  M  H  D  Y  L  T  G  G  F  T  A  N  T  S  L
Lathraea squamaria          AATGCTACTGCGGGTACATGCGAAGAAATGATGAAAAGAGCTGTATTTGCTAGAGAATTGGGAGTTCCTATCGTAATGCATGACTACTTAACAGGCGGATTCACTGCAAATACTAGCTTG
                             N  A  T  A  G  T  C  E  E  M  M  K  R  A  V  F  A  R  E  L  G  V  P  I  V  M  H  D  Y  L  T  G  G  F  T  A  N  T  S  L
Bartsia inaequalis          AATGCTACTGCGGGTACATGCGAAGAAATGATGAAAAGAGCTGTATTTGCTAGAGAATTGGGAGTTCCTATCGTAATGCATGACTACTTAACAGGAGGATTCACTGCAAATACTAGCTTG
                             N  A  T  A  G  T  C  E  E  M  M  K  R  A  V  F  A  R  E  L  G  V  P  I  V  M  H  D  Y  L  T  G  G  F  T  A  N  T  S  L

                            850 860 870 880 890 900 910 920 930 940 950 960
L.squamaria minor variant   GCTCATTATTGCCGAGATAATGGCCTACTTCTTCACATTCACCGTGCAATGCACGCTGTTATTGATAGACAGAAGAATCACGGTATACACTTCCGTGTACTAGCTAAAGCATTACGTATG
                             A  H  Y  C  R  D  N  G  L  L  L  H  I  H  R  A  M  H  A  V  I  D  R  Q  K  N  H  G  I  H  F  R  V  L  A  K  A  L  R  M
Lathraea squamaria          GCTCATTATTGCCGAGATAATGGCCTACTTCTTCACATTCACCGTGCAATGCACGCTGTTATTGATAGACAGAAGAATCACGGTATACACTTCCGTGTACTAGCTAAAGCATTACGTATG
                             A  H  Y  C  R  D  N  G  L  L  L  H  I  H  R  A  M  H  A  V  I  D  R  Q  K  N  H  G  I  H  F  R  V  L  A  K  A  L  R  M
Bartsia inaequalis          GCTCATTATTGCCGAGATAATGGCCTACTTCTTCACATTCACCGTGCAATGCACGCCGTTATTGATAGACAGAAGAATCACGGTATACACTTCCGTGTACTAGCTAAAGCGTTACGTATG
                             A  H  Y  C  R  D  N  G  L  L  L  H  I  H  R  A  M  H  A  V  I  D  R  Q  K  N  H  G  I  H  F  R  V  L  A  K  A  L  R  M

                            970 980 990 1000 1010 1020 1030 1040 1050 1060 1070 1080
L.squamaria minor variant   TCTGGTGGAGATCATATTCACTCTGGTACCGTAGTAGGTAAACTTGAAGGAGAAAGAGACATCACTTTGGGCTTTGTTGATTTACTGCGCGATTATTTTATTGAAAAAGATCGAAGTCGC
                             S  G  G  D  H  I  H  S  G  T  V  V  G  K  L  E  G  E  R  D  I  T  L  G  F  V  D  L  L  R  D  Y  F  I  E  K  D  R  S  R
Lathraea squamaria          TCTGGTGGAGATCATATTCACTCTGGTACCGTAGTAGGTAAACTTGAAGGAGAAAGAGACATCACTTTGGGCTTTGTTGATTTACTGCGCGATTATTTTATTGAAAAAGATCGAAGTCGC
                             S  G  G  D  H  I  H  S  G  T  V  V  G  K  L  E  G  E  R  D  I  T  L  G  F  V  D  L  L  R  D  Y  F  I  E  K  D  R  S  R
Bartsia inaequalis          TCTGGTGGAGATCATATTCACTCTGGTACCGTAGTAGGGAAACTTGAAGGAGAAAGAGATATCACTTTGGGCTTTGTTGATTTACTGCGTGATGATTTTATTGAAAAAGATCGAAGTCGC
                             S  G  G  D  H  I  H  S  G  T  V  V  G  K  L  E  G  E  R  D  I  T  L  G  F  V  D  L  L  R  D  D  F  I  E  K  D  R  S  R

                            1090 1100 1110 1120 1130 1140 1150 1160 1170 1180 1190 1200
L.squamaria minor variant   GGTATTTATTTCACTCAAGATTGGGTCTCTCTACCAGGTGTTATTCCCGTGGCTTCAGGGGGTATTCACGTTTGGCATATGCCTGCTCTGACCGAGATCTTTGGGGATGATTCCGTACTA
                             G  I  Y  F  T  Q  D  W  V  S  L  P  G  V  I  P  V  A  S  G  G  I  H  V  W  H  M  P  A  L  T  E  I  F  G  D  D  S  V  L
Lathraea squamaria          GGTATTTATTTCACTCAAGATTGGGTCTCTCTACCAGGTGTTATTCCCGTGGCTTCAGGGGGTATTCACGTTTGGCATATGCCTGCTCTGACCGAGATCTTTGGGGATGATTCCGTACTA
                             G  I  Y  F  T  Q  D  W  V  S  L  P  G  V  I  P  V  A  S  G  G  I  H  V  W  H  M  P  A  L  T  E  I  F  G  D  D  S  V  L
Bartsia inaequalis          GGTATTTATTTCACTCAAGATTGGGTCTCTATGCCAGGTGTTATTCCCGTGGCTTCAGGGGGTATTCACGTTTGGCATATGCCTGCTCTGACCGAGATCTTTGGGGATGATTCGGTACTA
                             G  I  Y  F  T  Q  D  W  V  S  M  P  G  V  I  P  V  A  S  G  G  I  H  V  W  H  M  P  A  L  T  E  I  F  G  D  D  S  V  L

                            1210 1220 1230 1240 1250 1260 1270 1280 1290 1300 1310 1320
L.squamaria minor variant   CAGTTCGGCGGAGGAACTTTAGGACACCCTTGGGGTAATGCACCGGGTGCCGTAGCTAACCGAGTAGCTCTAGAAGCATGTGTACAAGCTCGTAATGAAGGACGTGATCTTGCTGCTGAG
                             Q  F  G  G  G  T  L  G  H  P  W  G  N  A  P  G  A  V  A  N  R  V  A  L  E  A  C  V  Q  A  R  N  E  G  R  D  L  A  A  E
Lathraea squamaria          CAGTTCGGCGGAGGAACTTTAGGACACCCTTGGGGTAATGCACCGGGTGCCGTAGCTAACCGAGTAGCTCTAGAAGCATGTGTACAAGCTCGTAATGAAGGACGTGATCTTGCTGCTGAG
                             Q  F  G  G  G  T  L  G  H  P  W  G  N  A  P  G  A  V  A  N  R  V  A  L  E  A  C  V  Q  A  R  N  E  G  R  D  L  A  A  E
Bartsia inaequalis          CAGTTCGGTGGAGGAACTTTAGGACACCCTTGGGGTAATGCACCGGGTGCCGTAGCTAACCGAGTAGCTCTAGAAGCATGTGTAAAAGCTCGTAATGAAGGACGTGATCTTGCTGCTGAG
                             Q  F  G  G  G  T  L  G  H  P  W  G  N  A  P  G  A  V  A  N  R  V  A  L  E  A  C  V  K  A  R  N  E  G  R  D  L  A  A  E

                            1330 1340 1350 1360 1370 1380 1390 1400 1410 1420 1430
L.squamaria minor variant   GGTAATGCAATTATCCGCGAGGCTAGCAAATGGAGTCCTGAACTAGCTGCCGCTTGTGAGGTATGGAAAGAGATCAAATTTGAGTTTCGCGCAGTAGATACTTTGGATAAG
                             G  N  A  I  I  R  E  A  S  K  W  S  P  E  L  A  A  A  C  E  V  W  K  E  I  K  F  E  F  R  A  V  D  T  L  D  K
Lathraea squamaria          GGTAATGCAATTATCCGCGAGGCTAGCAAATGGAGTCCTGAACTAGCTGCCGCTTGTGAGGTATGGAAAGAGATCAAATTTGAGTTTCGCGCAGTAGATACTTTGGATAAG
                             G  N  A  I  I  R  E  A  S  K  W  S  P  E  L  A  A  A  C  E  V  W  K  E  I  K  F  E  F  R  A  V  D  T  L  D  K
Bartsia inaequalis          GGTAATGCAATTATCCGCGAGGCTAGCAAATGGAGTCCTGAACTAGCTGCCGCTTGTGAGGTATGGAAAGAGATCAAATTTGAGTTTCGCGCAGTAGATACTTTGGATAAG
                             G  N  A  I  I  R  E  A  S  K  W  S  P  E  L  A  A  A  C  E  V  W  K  E  I  K  F  E  F  R  A  V  D  T  L  D  K
```

ndhA

```
                                     10        20        30        40        50        60        70        80        90       100       110       120
                             ....|....|....|....|....|....|....|....|....|....|....|....|....|....|....|....|....|....|....|....|....|....|....|....|
Lindenbergia_philippensis    ATGATAATTGATACAACAGAAATACAAGCTATCAATTCTTTTTCTAGATTGGAATCCTTA~~~~~~AAAGAAGTTTACGGAATGATATGGATGCTTGGCCCTATTTTAACTCTTGTATTA
                              M  I  I  D  T  T  E  I  Q  A  I  N  S  F  S  R  L  E  S  L        K  E  V  Y  G  M  I  W  M  L  G  P  I  L  T  L  V  L
Bartsia_inaequalis           ATGATAATGGATACAATAGAAATACAAGCTATCAATTCTTTTTCCGGATTGGAATCCTTAAACTTAAAAGAAGTCTATGGAATTATATGGCTGTTTAGCCCGATTTTAACGCTTGTATTA
                              M  I  M  D  T  I  E  I  Q  A  I  N  S  F  S  G  L  E  S  L  N  L  K  E  V  Y  G  I  I  W  L  F  S  P  I  L  T  L  V  L
Lathraea_squamaria           ATGATAATGTATACAATAGAAATACAAGCTATCAATTCTTTTTCCGGATTGGAATCCTTA~~~~~~AAAGAAGTCTATGGTATTATATGGATGTTTATCCCTATTTTAACGCTTGTATTA
                              M  I  M  Y  T  I  E  I  Q  A  I  N  S  F  S  G  L  E  S  L        K  E  V  Y  G  I  I  W  M  F  I  P  I  L  T  L  V  L

                                    130       140       150       160       170       180       190       200       210       220       230       240
                             ....|....|....|....|....|....|....|....|....|....|....|....|....|....|....|....|....|....|....|....|....|....|....|....|
Lindenbergia_philippensis    GGAATCACACTAGGTGTACTAGTAATTGTTTGGTTAGAAAGAGAAATATCTGCAGGGATACAACAACGTATTGGACCTGAATACGCCGGGCCTTTGGGACTTCTGCAAGCTCTAGCAGAT
                              G  I  T  L  G  V  L  V  I  V  W  L  E  R  E  I  S  A  G  I  Q  Q  R  I  G  P  E  Y  A  G  P  L  G  L  L  Q  A  L  A  D
Bartsia_inaequalis           GGAATTACACTAGGCGTATTAGTAATTGTTTGGTTAGAAAGAGAAATATCTGCGGCGATACAACAACGTATTGGACCCGAATACGCCGGGCCGTTGGGAATTCTACAAGCTATAGCAGAC
                              G  I  T  L  G  V  L  V  I  V  W  L  E  R  E  I  S  A  A  I  Q  Q  R  I  G  P  E  Y  A  G  P  L  G  I  L  Q  A  I  A  D
Lathraea_squamaria           GGAATCACACTAGGCTTATTAGTAATTGTTTGGTTAGAAAGAGAAATATCTGCGGCGATACAACAACGTATTGGACCCGAATA-GCCGGGCCTTTGGGAATTCTGCAAGCTATAGCAGAC
                              G  I  T  L  G  L  L  V  I  V  W  L  E  R  E  I  S  A  A  I  Q  Q  R  I  G  P  E  *   P  G  L  W  E  F  C  K  L  *  Q  T

                                    250       260       270       280       290       300       310       320       330       340       350       360
                             ....|....|....|....|....|....|....|....|....|....|....|....|....|....|....|....|....|....|....|....|....|....|....|....|
Lindenbergia_philippensis    GGTACAAAACTACTTTTC~~~AAAGAGAATCTTCTTCCATCTAGAGGAGATACTCGTTTATTCAGTATCGGACCATCCATAGCAGTCATATCAATTTTACTAAGTTATTCAGTAATTCCT
                              G  T  K  L  L  F     K  E  N  L  L  P  S  R  G  D  T  R  L  F  S  I  G  P  S  I  A  V  I  S  I  L  L  S  Y  S  V  I  P
Bartsia_inaequalis           GGTACAAAACTACTTTTT~~~AAAGAAAATCTTCTTCCGTCTAGAGGGGATATTCGTTTATTCAGCATCGGACCATCCATAGCAGTCATATCAATTTTACTAACTTATTTAGTAATTCCT
                              G  T  K  L  L  F     K  E  N  L  L  P  S  R  G  D  I  R  L  F  S  I  G  P  S  I  A  V  I  S  I  L  L  T  Y  L  V  I  P
Lathraea_squamaria           GGTACAAAACTACTTTAT~~CAAAGAGAATCTTCTTCCGTCTAGAGGAGATATTCGTTTATTCAGTATCGGACCATCCATAGCAGTCATATCAATTTTACTAACTTATTTAGTAATTCCT
                               V  Q  N  Y  F  I    K  E  N  L  L  P  S  R  G  D  I  R  L  F  S  I  G  P  S  I  A  V  I  S  I  L  L  T  Y  L  V  I  P

                                    370       380       390       400       410       420       430       440       450       460       470       480
                             ....|....|....|....|....|....|....|....|....|....|....|....|....|....|....|....|....|....|....|....|....|....|....|....|
Lindenbergia_philippensis    TTTAGCTATCGCTTTATTCTAGCTGATCTTAGTATTGGTATTTTTTTATGGATTGCCGTTTCAAGCCTTGCTCCCGTTGGACTTCTTATGTCGGGGTATGGATCAAATAATAAATATTCC
                              F  S  Y  R  F  I  L  A  D  L  S  I  G  I  F  L  W  I  A  V  S  S  L  A  P  V  G  L  L  M  S  G  Y  G  S  N  N  K  Y  S
Bartsia_inaequalis           TTTAGCCATCGCTTTATTCTAGCTGATCTTAGTATTGGTATTTTTTTATGGATTGCCGTTTCCAGCCTTGCTCCCGTTGGACTTCTTATGTCGGGATATGGCTCAAATAATAAATATTCC
                              F  S  H  R  F  I  L  A  D  L  S  I  G  I  F  L  W  I  A  V  S  S  L  A  P  V  G  L  L  M  S  G  Y  G  S  N  N  K  Y  S
Lathraea_squamaria           TTTAGCTATCGCTTTATTCTAGCTTATCTTAGTATTGGTATTTTTATATGGATTGCCGTTTCGAGTCTTGCTCCCGTTGGACTTCTTATGTCAGGATATGGCTCAAATAATAAA~~~~~~
                              F  S  Y  R  F  I  L  A  Y  L  S  I  G  I  F  I  W  I  A  V  S  S  L  A  P  V  G  L  L  M  S  G  Y  G  S  N  N  K

                                    490       500       510       520       530       540       550       560       570       580       590       600
                             ....|....|....|....|....|....|....|....|....|....|....|....|....|....|....|....|....|....|....|....|....|....|....|....|
Lindenbergia_philippensis    TTTTTAGGTGGATTAAGGGCTGCTGCTCAATCAATTAGTTATGAAATACCATTAACTCTATGTGTATTATCAATATCTCTACTATCTAACAGTTTAAGTACAGTTGATATAGTTGAAGTG
                              F  L  G  G  L  R  A  A  A  Q  S  I  S  Y  E  I  P  L  T  L  C  V  L  S  I  S  L  L  S  N  S  L  S  T  V  D  I  V  E  V
Bartsia_inaequalis           TTTTTAGGTGGATTAAGGGCTGCTGCTCAATCAATTAGTTATGAAATACCATTAACTCTATGTGTATTATCAATATCTCTACTATCTAACAGTTTAAGTACAGTTGATATAGTTGAAGCG
                              F  L  G  G  L  R  A  A  A  Q  S  I  S  Y  E  I  P  L  T  L  C  V  L  S  I  S  L  L  S  N  S  L  S  T  V  D  I  V  E  A
Lathraea_squamaria           ~~~TTAGGTGGATTAAGGGTTGCTGCTCAATCAATTAGTTATGAAATACCATTAACTCTATGTGTATTATCAATATCTCTACTATCTAACAGTTTAAGTACAGTTGATATAGTTGAAGCG
                                 L  G  G  L  R  V  A  A  Q  S  I  S  Y  E  I  P  L  T  L  C  V  L  S  I  S  L  L  S  N  S  L  S  T  V  D  I  V  E  A

                                    610       620       630       640       650       660       670       680       690       700       710       720
                             ....|....|....|....|....|....|....|....|....|....|....|....|....|....|....|....|....|....|....|....|....|....|....|....|
Lindenbergia_philippensis    CAGTCAAAATATGGTTTATGGGGGTGGAATTTCTGGCGTCAACCTATAGGGTTTATTGTTTTTCTAATTTCTTCTCTAGCCGAGTGTGAGAGATTACCTTTTGATTTACCAGAAGCAGAA
                              Q  S  K  Y  G  L  W  G  W  N  F  W  R  Q  P  I  G  F  I  V  F  L  I  S  S  L  A  E  C  E  R  L  P  F  D  L  P  E  A  E
Bartsia_inaequalis           CAGTCGAAATATGGTTTTTGGGGATGGAATTTATGGCGCCAACCCATAGGGTTTATCGTTTTTCTAATTTCGTCTCTAGCCGAGTGTGAGAGATTGCCTTTTGATTTACCAGAAGCAGAA
                              Q  S  K  Y  G  F  W  G  W  N  L  W  R  Q  P  I  G  F  I  V  F  L  I  S  S  L  A  E  C  E  R  L  P  F  D  L  P  E  A  E
Lathraea_squamaria           CAGTCAAAATATGGTTTTTGGGGATGGAATTTATGGCGCCAACCTGTAAGGTTTATCGTTTTTCTAATTTCGTCTCTAGCCGAGTGTGAGAGATTGCCTTTTGATTTACCAGAAGCAGAA
                              Q  S  K  Y  G  F  W  G  W  N  L  W  R  Q  P  V  R  F  I  V  F  L  I  S  S  L  A  E  C  E  R  L  P  F  D  L  P  E  A  E
```

```
                                       730       740       750       760       770       780       790       800       810       820       830       840
                                ....|....|....|....|....|....|....|....|....|....|....|....|....|....|....|....|....|....|....|....|....|....|....|....|
Lindenbergia_philippensis       GAAGAATTAGTAGCAGGTTATCAAACCGAATATTCCGGTATAAAATTTGGTTTATTTTACGTTGCTTCGTATCTAAATCTACTAGTTTCTTCATTATTTGTAACAGTTCTTTATTTGGGG
                                 E  E  L  V  A  G  Y  Q  T  E  Y  S  G  I  K  F  G  L  F  Y  V  A  S  Y  L  N  L  L  V  S  S  L  F  V  T  V  L  Y  L  G
Bartsia_inaequalis              GAAGAATTAGTGGCAGGTTATCAAACCGAATATTCAGGTATCAAATTTGGTTTATTTTACGTTGCTTCGTATCTAAATCTACTAGTTTCTTCATTGTTTGTAACAGTTCTTTACTTGGGG
                                 E  E  L  V  A  G  Y  Q  T  E  Y  S  G  I  K  F  G  L  F  Y  V  A  S  Y  L  N  L  L  V  S  S  L  F  V  T  V  L  Y  L  G
Lathraea_squamaria              GAAGAATTAGTGGCAGGTTATCAAACCGAATATTCAGGTATCAAATTTGGTTTATTTTACGTTGCTTCGTATCTAAATCTACTAGTTTCTTCATTGTTTGTAACAGTTCTTTACTTGGGG
                                 E  E  L  V  A  G  Y  Q  T  E  Y  S  G  I  K  F  G  L  F  Y  V  A  S  Y  L  N  L  L  V  S  S  L  F  V  T  V  L  Y  L  G

                                       850       860       870       880       890       900       910       920       930       940       950       960
                                ....|....|....|....|....|....|....|....|....|....|....|....|....|....|....|....|....|....|....|....|....|....|....|....|
Lindenbergia_philippensis       GGTTGGAATCTTTCTATTCCATACATACCCGTTCCTGAGCTTTTTGACATAAATAAAAGAAGTCAAGTTTTTGGAACAACAGTCGGTATCTTTATTACATTAGCTAAAACTTATTTGTTC
                                 G  W  N  L  S  I  P  Y  I  P  V  P  E  L  F  D  I  N  K  R  S  Q  V  F  G  T  T  V  G  I  F  I  T  L  A  K  T  Y  L  F
Bartsia_inaequalis              GGTTGGAATCTTTCTATTCCATACATATCCGTTCCTGGACTTTTTGACATAAATAAAAGAAGTCAAGTTTTTGGAATAATAATCGGTATCTTTATTACATTAGCTAAAACTTATTTGTTC
                                 G  W  N  L  S  I  P  Y  I  S  V  P  G  L  F  D  I  N  K  R  S  Q  V  F  G  I  I  I  G  I  F  I  T  L  A  K  T  Y  L  F
Lathraea_squamaria              GGTTGGAATCTTTCTATTCCATACATACCCGTTCCTGGGCTTTTTGACATAAATAAAAGAAGTCAAGTTTTTGGAACAACAATAGGTATCTTTATTACATTAGCTAAAACTTATTTGTTC
                                 G  W  N  L  S  I  P  Y  I  P  V  P  G  L  F  D  I  N  K  R  S  Q  V  F  G  T  T  I  G  I  F  I  T  L  A  K  T  Y  L  F

                                       970       980       990      1000      1010      1020      1030      1040      1050      1060      1070      1080
                                ....|....|....|....|....|....|....|....|....|....|....|....|....|....|....|....|....|....|....|....|....|....|....|....|
Lindenbergia_philippensis       TTGTTCATTTCTATTGCAACAAGATGGACTTTGCCGAGACTGAGAATGGACCAACTATTAAATCTTGGCTGGAAATTTCTTTTACCCATTTCTTTAGGTAATCTATTATTAACAACTTCG
                                 L  F  I  S  I  A  T  R  W  T  L  P  R  L  R  M  D  Q  L  L  N  L  G  W  K  F  L  L  P  I  S  L  G  N  L  L  L  T  T  S
Bartsia_inaequalis              TTGTTCATTGCTATTGCAACAAGATGGACTTTACCGAGACTACGAATGGACCAATTATTAAATCTTGGATGGAAATTTCTTTTACCTATTTCGCTAGGTAATCTATTATTAACAACTTCG
                                 L  F  I  A  I  A  T  R  W  T  L  P  R  L  R  M  D  Q  L  L  N  L  G  W  K  F  L  L  P  I  S  L  G  N  L  L  L  T  T  S
Lathraea_squamaria              TTGTTCATTGCTATTGTAACAAGATGGACTTTACCGAGACTAAGAATCGACCAACTATTAAATCTTGGATGGAAATTTCTTTTACCTATTTCGCTAGGTAATCTATTATTAACAACTTCG
                                 L  F  I  A  I  V  T  R  W  T  L  P  R  L  R  I  D  Q  L  L  N  L  G  W  K  F  L  L  P  I  S  L  G  N  L  L  L  T  T  S

                                      1090      1100
                                ....|....|....|....|.
Lindenbergia_philippensis       TCCCAACTTCTTTCACTGTAA
                                 S  Q  L  L  S  L  *
Bartsia_inaequalis              TCCCAACTTTTTTCACTCTAA
                                 S  Q  L  F  S  L  *
Lathraea_squamaria              TCCCAACTTCTTTCACTCTAA
                                 S  Q  L  L  S  L  *
```

==

ndhJ

```
                                        10        20        30        40        50        60        70        80        90       100       110       120
                                ....|....|....|....|....|....|....|....|....|....|....|....|....|....|....|....|....|....|....|....|....|....|....|....|
Lindenbergia philippensis       ATGCGGGGTCGTTTGTCTGCTTGGCTAGTCAAGCACGGGATAATTCATAGATCTTTGGGCTTTGATTATCAAGGAATAGAGACTTTACAAATCAAGTCTGAGGATTGGCATTCCATCGCT
                                 M  R  G  R  L  S  A  W  L  V  K  H  G  I  I  H  R  S  L  G  F  D  Y  Q  G  I  E  T  L  Q  I  K  S  E  D  W  H  S  I  A
Schwalbea americana             GTGCAGGGTCTTTTGTCTGCTTGGCTAGTCAAGCATGGGATAATTCATAGATCTTTGGGTTTTGATTATCAAGGAATAGAGACTTTACAAATAAAGTCTGAGGATTCGCATTCCATTGCT
                                 M  Q  G  L  L  S  A  W  L  V  K  H  G  I  I  H  R  S  L  G  F  D  Y  Q  G  I  E  T  L  Q  I  K  S  E  D  S  H  S  I  A
Bartsia inaequalis              ATGCAGGGTCATTTGTCTGCTTGGTTAGTTAAGCATGGGATAATTCATAGATCTTTGGGCTTTGATTATCAAGGAATAGAGACTTTACAAATAAAGTTTGAGGATTGGCATTCCATTGCT
                                 M  Q  G  H  L  S  A  W  L  V  K  H  G  I  I  H  R  S  L  G  F  D  Y  Q  G  I  E  T  L  Q  I  K  F  E  D  W  H  S  I  A
Lathraea squamaria              ATGCAGGGTCGTTTGTCTGCTTGGTTAGTTAAGCATGGGATAATTCATAGATCTTTGGGCTTTGATTATCAAGGAATAGAGACTTTACAAATAAAGTTTGAGGATTGGCATTCCATTGCT
                                 M  Q  G  R  L  S  A  W  L  V  K  H  G  I  I  H  R  S  L  G  F  D  Y  Q  G  I  E  T  L  Q  I  K  F  E  D  W  H  S  I  A

                                       130       140       150       160       170       180       190       200       210       220       230       240
                                ....|....|....|....|....|....|....|....|....|....|....|....|....|....|....|....|....|....|....|....|....|....|....|....|
Lindenbergia philippensis       GTCATTTTATATGTATATGGTTACAATTATTTACGTTCCCAATGTGCCTATGATGTAGCACCTGGCGGACTGTTAGCTAGTGTGTATCATCTTACCAGAATAGAGTATGGTGTGGATCAA
                                 V  I  L  Y  V  Y  G  Y  N  Y  L  R  S  Q  C  A  Y  D  V  A  P  G  G  L  L  A  S  V  Y  H  L  T  R  I  E  Y  G  V  D  Q
Schwalbea americana             GTCATTTTATATGTATATGGTTACAATTATTTACGCTCCCAGTGTGCTTATGAGGTAGCACCGGGCGGATTGTTAGCTAGT~~~~~TCATCTTACCAGACTAGAGTATGGTGTGGATCAA
```

```
                               V  I  L  Y  V  Y  G  Y  N  Y  L  R  S  Q  C  A  Y  E  V  A  P  G  G  L  L  A  S        S  S  Y  Q  T  R  V  W  C  G  S
Bartsia inaequalis            GTCATTTTATATGTATATGGTTACAATTATTTACGCTCCCAATGTGCCTATGATGTAGCACCTGGCGGACTGTTAGCTAGTGTGTATCATCTTACTAGAATAGAGTATGGTGTGGATCAA
                               V  I  L  Y  V  Y  G  Y  N  Y  L  R  S  Q  C  A  Y  D  V  A  P  G  G  L  L  A  S  V  Y  H  L  T  R  I  E  Y  G  V  D  Q
Lathraea squamaria            GTCATTTTATATGTATATGGTTACAATTATTTACGCTCCCAATGTGCCTATGATGTAGCACCTGGCGGACTGTTAGCTAGTGTGTATCATCTTACCAGAATAGAGTATGGTGTGGATCAA
                               V  I  L  Y  V  Y  G  Y  N  Y  L  R  S  Q  C  A  Y  D  V  A  P  G  G  L  L  A  S  V  Y  H  L  T  R  I  E  Y  G  V  D  Q
```

```
                                      250       260       270       280       290       300       310       320       330       340       350       360
                              ....|....|....|....|....|....|....|....|....|....|....|....|....|....|....|....|....|....|....|....|....|....|....|....|
Lindenbergia philippensis     CCAGAAGAAGTATGCATAAAAGTATTTGCCTCAAGGAGGAATCCTAGAATTCCGTCCGTTTTCTGGGTTTGGAAAAGCGTGGATTTTCAAGAACGAGAATCCTATGATATGTTGGGAATC
                               P  E  E  V  C  I  K  V  F  A  S  R  R  N  P  R  I  P  S  V  F  W  V  W  K  S  V  D  F  Q  E  R  E  S  Y  D  M  L  G  I
Schwalbea americana           CCAGAAGAAGTATGCATAAAAGTATTTGCCTCAAGGAAGAATCCTAGAATTCCGTCCGCTTTCTGGGTTTGGAAAAGTGTGGATTTTCAAGAACGAGAATCCTATGATATGTTGGGAATC
                              T  R  R  S  M  H  K  S  I  C  L  K  E  E  S  *  N  S  V  R  F  L  G  L  E  K  C  G  F  S  R  T  R  I  L  *  Y  V  G  N
Bartsia inaequalis            CCGGAAGAGGTATGCATAAAAGTATTTGCCGCAAGGAGGAATCCTAGAATTCCGTCTGCTTTCTGGGTTTGGAAAAGCGCGGATTTTCAAGAACGAGAATCCTATGATATGTTGGGAATC
                               P  E  E  V  C  I  K  V  F  A  A  R  R  N  P  R  I  P  S  A  F  W  V  W  K  S  A  D  F  Q  E  R  E  S  Y  D  M  L  G  I
Lathraea squamaria            CCGGAAGAGGTATGCATAAAAGTATTTGCCGCAAGAAGGAATCCCAGAATTCCGTCCGCTTTCTGGGTTTGGAAAAGCGCGGATTTTCAAGAACGGGAATCCTATG~~~~~TCGAGAATC
                               P  E  E  V  C  I  K  V  F  A  A  R  R  N  P  R  I  P  S  A  F  W  V  W  K  S  A  D  F  Q  E  R  E  S  Y        V  E  N
```

```
                                      370       380       390       400       410       420       430       440       450       460       470
                              ....|....|....|....|....|....|....|....|....|....|....|....|....|....|....|....|....|....|....|....|....|....|....|..
Lindenbergia philippensis     TCTTATGATAATCATCCGCGCTTGAAACGTATCTTAATGCCTGAAAGTTGGATAGGATGGCCCCTGCGTAAGGATTATATTGCCCCCAATTTTTATGAAATACAAGATGCTCATTGA
                               S  Y  D  N  H  P  R  L  K  R  I  L  M  P  E  S  W  I  G  W  P  L  R  K  D  Y  I  A  P  N  F  Y  E  I  Q  D  A  H  *
Schwalbea americana           TCTTATGATAATCATCCGCGCTTGAAACGTATCTTAATGCCTGGAAGTTGGATAGGATGGCCCTTACGTAAGGATTATATTGCCCCCAATTTTTATGAAATACAAGATGCTCATTGA
                              L  L  *  *  S  S  A  L  E  T  Y  L  N  A  W  K  L  D  R  M  A  L  T  *  G  L  Y  C  P  Q  F  L  *  N  T  R  C  S  L
Bartsia inaequalis            TCTTATGATAATCATCCGCGCTTGAAACGTATCTTAATGCCGGAAAGTTGGATAGGATGGCCCTTACGTAAGGATTATATTGCCCCCAATTTTTATGAAATTCAAGATGCTCATTGA
                               S  Y  D  N  H  P  R  L  K  R  I  L  M  P  E  S  W  I  G  W  P  L  R  K  D  Y  I  A  P  N  F  Y  E  I  Q  D  A  H  *
Lathraea squamaria            TCTTATGATAATCATCCGCGCTTGAAACGTATCTTAATGCCGGAAAGTTGGATAGGATGGCCCTTACGTAAGGATTATATTGCCCCCAATTTTTATGAAATACAAGATGCTCATTGA
                              L  L  *  *  S  S  A  L  E  T  Y  L  N  A  G  K  L  D  R  M  A  L  T  *  G  L  Y  C  P  Q  F  L  *  N  T  R  C  S  L
```

===

ndhB

```
                                       10        20        30        40        50        60        70        80        90       100       110       120
                               ....|....|....|....|....|....|....|....|....|....|....|....|....|....|....|....|....|....|....|....|....|....|....|....|
L.squamaria minor_sequence     ATGATCTGGCATGTACAGAATGAAgttTTCATTCTCGATTCTACGAGAATTTTTATGAAAGCCTTTCATTTGCTTCTCTTCGATGGAAGTTTGATTTTCCCAGAATGTATCCTAATTTTT
                                M  I  W  H  V  Q  N  E  V  F  I  L  D  S  T  R  I  F  M  K  A  F  H  L  L  L  F  D  G  S  L  I  F  P  E  C  I  L  I  F
Lathraea squamaria             ATGATCTGGCATGTACAGAATGAAAACTTCATTCTCGATTCTACGAGAATTTTTATGAAAGCCTTTCATTTGCTTCTCTTCGATGGAAGTTTGATTTTCCCAGAATGTATCCTAATTTTT
                                M  I  W  H  V  Q  N  E  N  F  I  L  D  S  T  R  I  F  M  K  A  F  H  L  L  L  F  D  G  S  L  I  F  P  E  C  I  L  I  F
Bartsia_inaequalis             ATGATCTGGCATGTACAGAATGAAAACTTCATTCTCGATTCTACGAGAATTTTTATGAAAGCCTTTCATTTGCTTCTATTCGATGGAAGTTTGATTTTCCCAGAATGTATCCTAATTTTT
                                M  I  W  H  V  Q  N  E  N  F  I  L  D  S  T  R  I  F  M  K  A  F  H  L  L  L  F  D  G  S  L  I  F  P  E  C  I  L  I  F
```

```
                                      130       140       150       160       170       180       190       200       210       220       230       240
                               ....|....|....|....|....|....|....|....|....|....|....|....|....|....|....|....|....|....|....|....|....|....|....|....|
L.squamaria minor_sequence     GGCCTAATTCTTCTTCTGATGATCGATTCAACCTCTGATCAAAAAGATATACCTTGGTT~~~~TTCATCTCTTCAACAAGTTTAGTAATGGGCATAATGGCCCTATTGTTCCGATGGAGA
                                G  L  I  L  L  L  M  I  D  S  T  S  D  Q  K  D  I  P  W  F        S  S  L  Q  Q  V  *  *  W  A  *  W  P  Y  C  S  D  G  E
Lathraea squamaria             GGCCTAATTCTTCTTCTGATGATCGATTCAACCTCTGATCAAAAAGATATACCTTGGTTATATTTCATCTCTTCAACAAGTTTAGTAATGGGCATAATGGCCCTATTGTTCCGATGGAGA
                                G  L  I  L  L  L  M  I  D  S  T  S  D  Q  K  D  I  P  W  L  Y  F  I  S  S  T  S  L  V  M  G  I  M  A  L  L  F  R  W  R
Bartsia_inaequalis             GGCCTAATTCTTCTTCTGATGATCGATTCAACCTCTGATCAAAAAGATATACCTTGGTTATATTTCATCTCTTCAACAAGTTTAGTAATGGGCATAACGGCCCTATTGTTCCGATGGAGA
                                G  L  I  L  L  L  M  I  D  S  T  S  D  Q  K  D  I  P  W  L  Y  F  I  S  S  T  S  L  V  M  G  I  T  A  L  L  F  R  W  R
```

```
                                      250       260       270       280       290       300       310       320       330       340       350       360
                               ....|....|....|....|....|....|....|....|....|....|....|....|....|....|....|....|....|....|....|....|....|....|....|....|
L.squamaria minor_sequence     GAAGAACCTATGATTCGCTTTTCGGGAAATTTCCAAACGAACAATTTCAACGAAATCTTTCAATTTCTTATTTTACTATGTTCAACTCTATGTATTCCTCTATCCGTAGAGTACATTGGA
                                 K  N  L  *  F  A  F  R  E  I  S  K  R  T  I  S  T  K  S  F  N  F  L  F  Y  Y  V  Q  L  Y  V  F  L  Y  P  *  S  T  L  D
Lathraea squamaria             GAAGAACCTATGATTCGCTTTTCGGGAAATTTCCAAACGAACAATTTCAACGAAATCTTTCAATTTCTTATTTTACTATGTTCAACTCTATGTATTCCTCTATCCGTAGAGTACATTGGA
                                E  E  P  M  I  R  F  S  G  N  F  Q  T  N  N  F  N  E  I  F  Q  F  L  I  L  L  C  S  T  L  C  I  P  L  S  V  E  Y  I  G
Bartsia_inaequalis             GAAGAACCTATGATTAGCTTTTCGGGAAATTTCCAAACGAACAATTTCAACGAAATCTTTCAATTTCTTATTTTACTATGTTCAACTCTATGTATTCCTCTATCCGTAGAGTACATTGAA
```

```
                              E E P M I S F S G N F Q T N N F N E I F Q F L I L L C S T L C I P L S V E Y I E

                                   370       380       390       400       410       420       430       440       450       460       470       480
                              ....|....|....|....|....|....|....|....|....|....|....|....|....|....|....|....|....|....|....|....|....|....|....|....|
L.squamaria minor_sequence    TGTACAGAAATGGCTATAACAGAGTTTCTCTTATTCGTATTAACAGCTACTCTAGGAGGAATGTTTTTATGCGGTGCTAACGATTTAATAACTATCTTTGTAGCTCCAGAATGTTTCAGT
                               V Q K W L * Q S F S Y S Y * Q L L * E E C F Y A V L T I * * L S L * L Q N V S V
Lathraea squamaria            TGTACAGAAATGGCTATAACAGAGTTTCTCTTATTCGTATTAACAGCTACTCTAGGAGGAATGTTTTTATGCGGTGCTAACGATTTAATAACTATCTTTGTAGCTCCAGAATGTTTCAGT
                               C T E M A I T E F L L F V L T A T L G G M F L C G A N D L I T I F V A P E C F S
Bartsia_inaequalis            TGTACAGAAATGGCTATAACAGAGTTTCTCTTATTCGTATTAACAGCTACTCTAGGAGGAATGTTTTTATGCGGTGCTAACGATTTAATAACTATCTTTGTAGCTCCAGAATGTTTCAGT
                               C T E M A I T E F L L F V L T A T L G G M F L C G A N D L I T I F V A P E C F S

                                   490       500       510       520       530       540       550       560       570       580       590       600
                              ....|....|....|....|....|....|....|....|....|....|....|....|....|....|....|....|....|....|....|....|....|....|....|....|
L.squamaria minor_sequence    TTATGC~~~TCCTACCTATTATCTGGATATACCAAGAAAGATGTACGGTCTAATGAGGCTACTATGAAATATTTACTCATGGGTGGGGCAAGCTCTTCTATTCTGGTTCATGGTTTCTCT
                               Y A   P T Y Y L D I P R K M Y G L M R L L * N I Y S W V G Q A L L F W F M V S L
Lathraea squamaria            TTATGG~~CTCCTACCTATTATCTGGATATACCAAGAAAGATGTACGGTCTAATGAGGCTACTATGAAATATTTACTCATGGGTGGGGCAAGCTCTTCTATTCTGGTTCATGGTTTCTCT
                               L W   L L P I I W I Y Q E R C T V * * G Y Y E I F T H G W G K L F Y S G S W F L
Bartsia_inaequalis            TTATGC~~~TCCTACCTATTATCTGGATATACCAAGAAAGATGTACGGTCTAATGAGGCTACTATGAAATATTTACTCATGGGTGGGGCAAGCTCTTCTATTCTGGTTCATGGTTTCTCT
                               L C   S Y L L S G Y T K K D V R S N E A T M K Y L L M G G A S S S I L V H G F S

                                   610       620       630       640       650       660       670       680       690       700       710       720
                              ....|....|....|....|....|....|....|....|....|....|....|....|....|....|....|....|....|....|....|....|....|....|....|....|
L.squamaria minor_sequence    TGGCTATATGGTTTATCCGGGGGAGAGATCGAGCTTCAAGAAATAGTGAATGGTCTTATCAATACACAAATGTATAACTCCCCAGGAATTTCAATTGCGCTCATATTCATCACTGTAGGA
                                G Y M V Y P G E R S S F K K * * M V L S I H K C I T P Q E F Q L R S Y S S L * E
Lathraea squamaria            TGGCTATATGGTTTATCCGGGGGAGAGATCGAGCTTCAAGAAATAGTGAATGGTCTTATCAATACACAAATGTATAACTCCCCAGGAATTTCAATTGCGCTCATATTCATCACTGTAGGA
                              L A I W F I R G R D R A S R N S E W S Y Q Y T N V * L P R N F N C A H I H H C R
Bartsia_inaequalis            TGGCTATATGGTTTATCCGGGGGAGAGATCGAGCTTCAAGAAATAGTGAATGGTCTTATCAATACACAAATGTATAACTCCCCAGGAATTTCAATTGCGCTCATATTCATCACTGTAGGA
                               W L Y G L S G G E I E L Q E I V N G L I N T Q M Y N S P G I S I A L I F I T V G

                                   730       740       750       760       770       780       790       800       810       820       830       840
                              ....|....|....|....|....|....|....|....|....|....|....|....|....|....|....|....|....|....|....|....|....|....|....|....|
L.squamaria minor_sequence    ATTGGGTTCAAGCTTTCCCCAGCCCCTTCTCAT~~~~GGACTCCTGACGTATACGAAGGAGTGCGGTTCGTTCGATAAATTCCTACCTCTCTATCTATCTCTGAGATGTTTGGATTTTTC
                                L G S S F P Q P L L M   D S * R I R R
Lathraea squamaria            ATTGGGTTCAAGCTTTCCCCAGCCCCTTCTCATCAATGGACTCCTGACGTATACGAAGGAGTGCGGTTCGTTCGATAAATTCCTACCTCTCTATCTATCTCTGAGATGTTTGGATTTTTC
                              N W V Q A F P S P F S S M D S * R I R R
Bartsia_inaequalis            ATTGGGTTCAAGCTTTCCCCAGCCCCTTCTCATCAATGGACTCCTGACGTATACGAAGGAGTGCGGTTCGTTCGAGAAATTCCTACCTCTCTATCTATCTCTGAGATGTTTGGATTTTTC
                               I G F K L S P A P S H Q W T P D V Y E G [ intron sequence

                                   850       860       870       880       890       900       910       920       930       940       950       960
                              ....|....|....|....|....|....|....|....|....|....|....|....|....|....|....|....|....|....|....|....|....|....|....|....|
L.squamaria minor_sequence    AAAACTCCACGGACATGCAGAAGAGAAATGCTATCCCCACTCGGACCAAGACAGAACTTTgACTTGTTCAAATAACAATTAAGGTGAAGCGGGGTCAGGAACGACGAATCTCTTTATGAT
Lathraea squamaria            AAAACTCCACGGACATGCAGAAGAGAAATGCTATCCCCACTCGGACCAAGACAGAACTTTTACTTGTTCAAATAACAATTAAGGTGAAGCGGGGTCAGGAACGACGAATCTCTTTATGAT
Bartsia_inaequalis            AAAACTCCACGGACATGCAGAAGAGAAATGCTATCCCCACTCGGACCAAGACAGAACTTTTACTTGTTCAAATAACAATTAAGGTGAAGCAGGGTCAGGAACGACGAATCTCTTTATGAT

                                   970       980       990      1000      1010      1020      1030      1040      1050      1060      1070      1080
                              ....|....|....|....|....|....|....|....|....|....|....|....|....|....|....|....|....|....|....|....|....|....|....|....|
L.squamaria minor_sequence    AAACAGATCCATTTTGCAAGTTCGTTATTACGGGTAGTTCCTACAAAGGATCGGACTAATGACGTATCCAATACTTGAATTCTCGATGTAgATGCTACATAGTTGGTTCTCATCCTTCAG
Lathraea squamaria            AAACAGATCCATTTTGCAAGTTCGTTATTACGGGTAGTTCCTACAAAGGATCGGACTAATGACGTATCCAATACTTGAATTCTCGATGTAAATGCTACATAGTTGGTTCTCATCCTTCAG
Bartsia_inaequalis            AAACAGATCCATTTTGCAAGTTCGTTATTACGGGTAGTTCCTACAAAGGATCGGACTAATGACGTATCCAATACTTGAATTCTCGATGTAGATGCTACATAGTTGGTTCTCATCCTTCAG

                                  1090      1100      1110      1120      1130      1140      1150      1160      1170      1180      1190      1200
                              ....|....|....|....|....|....|....|....|....|....|....|....|....|....|....|....|....|....|....|....|....|....|....|....|
L.squamaria minor_sequence    AGACTACGAGTGTAATAGGAGCATCCGTCGACAAAAGGATCACCCTAAGATGATCATTTCATGGCTATTGAGAACGAATTAAATCAGATGGTTCTATTTCTCAATCTTTCTGACTTGCTC
Lathraea squamaria            AGACTACGAGTGTAATAGGAGCATCCGTCGACAAAAGGATCACCCTAAGATGATCATTTCATGGCTATTGAGAACGAATTAAATCAGATGGTTCTATTTCTCAATCTTTCTGACTTGCTC
Bartsia_inaequalis            AGACTACGAGTGTAATAGGAGCATCCGTCGACAAAAGGATCACCCTAAGATGATCATTTCATGGCTATTGAGAACGAATTAAATCAGATGGTTCTATTTCTCAATCTTTCTGACTTGCTC

                                  1210      1220      1230      1240      1250      1260      1270      1280      1290      1300      1310      1320
                              ....|....|....|....|....|....|....|....|....|....|....|....|....|....|....|....|....|....|....|....|....|....|....|....|
```

```
L.squamaria minor_sequence    CTAcggaaccaaggTCGAAAAGATTGAAAAAATCAGTCATTCACAACCACTGATGAAGGATTCCTCGAAAAGTgAAGGATTAGTAATCTTTTTTAGAAATCGAATGGATTCGGTCTTATA
Lathraea squamaria            CTAC~~~~~~~~~~TCGAAAAGATTGAAAAAATCAGTCATTCACAACCACTGATGAAGGATTCCTCGAAAAGTTAAGGATTAGTAATCTTTTTTAGAAATCGAATGGATTCGGTCTTATA
Bartsia_inaequalis            CTACGGAACCAAGGTCGAAAAGATTGAAAAAATCAGTCATTCACAACCACTGATGAAGGATTCCTCGAAAAGTTAAGGATTAGTAATCTTTTTTAGAAATCGAATGGATTCGGTCTTATA

                                    1330      1340      1350      1360      1370      1380      1390      1400      1410      1420      1430      1440
                              ....|....|....|....|....|....|....|....|....|....|....|....|....|....|....|....|....|....|....|....|....|....|....|....|
L.squamaria minor_sequence    CATACGCGAGGAAGGTAATCAAAAAAGAAAGAAGATGCGTTCTTCTGTCTTTTATCACTTAGGAGCCGTGTGAGATGAAAGtCTCATGCACGGTTTTGAATGAGAGAAAGAAGTGAGGAA
Lathraea squamaria            CATACGCGAGGAAGGTAATCAAAAAAGAAAGAAGATGCGTTCTTCTGTCTTTTATCACTTAGGAGCCGTGTGAGATGAAAGGCTCATGCACGGTTTTGAATGAGAGAAAGAAGTGAGGAA
Bartsia_inaequalis            CATACGCGAGGAAGGTAATCAAAAAAGAAAGAAGATGCGTTCTTCTGTCTTTTATCACTTAGGAGCCGTGTGAGATGAAAGTCTCATGCACGGTTTTGAATGAGAGAAAGAAGTGAGGAA

                                    1450      1460      1470      1480      1490      1500      1510      1520      1530      1540      1550      1560
                              ....|....|....|....|....|....|....|....|....|....|....|....|....|....|....|....|....|....|....|....|....|....|....|....|
L.squamaria minor_sequence    TCCTCTTTTCGACTCTAAC~~TCTCCCACTCCAGTCGTTGCTTTTCTTTCTGTTACTTCGAAAGTAGCTGCTCCAGCTTCAGCCACTCGAATTTTaGATATTCCTTTTTATTTCTCATCA
                                                   L  S  H  S  S  R  C  F  S  F  C  Y  F  E  S  S  C  S  S  F  S  H  S  N  F  R  Y  S  F  L  F  L  I
Lathraea squamaria            TCCTCTTTTCGACTCTAAC~~TCTCCCACTCCAGTCGTTGCTTTTCTTTCTGTTACTTCGAAAGTAGCTGCTCCAGCTTCAGCCACTCGAATTTTCGATATTCCTTTTTATTTCTCATCA
                                                   L  S  H  S  S  R  C  F  S  F  C  Y  F  E  S  S  C  S  S  F  S  H  S  N  F  R  Y  S  F  L  F  L  I
Bartsia_inaequalis            TCCTCTTTTCGACTCTGAC~~TCTCCCACTCCAGTCGTTGCTTTTCTTTCTGTTACTTCGAAAGTAGCTGCTCCAGCTTCAGCCACTCGAATTTTCGATATTCCGTTTTATTTCTCATCA
                               intron sequence   ]  S  P  T  P  V  V  A  F  L  S  V  T  S  K  V  A  A  P  A  S  A  T  R  I  F  D  I  P  F  Y  F  S  S

                                    1570      1580      1590      1600      1610      1620      1630      1640      1650      1660      1670      1680
                              ....|....|....|....|....|....|....|....|....|....|....|....|....|....|....|....|....|....|....|....|....|....|....|....|
L.squamaria minor_sequence    AACGAATGGCATCTTCTTCTGGAAATCCTAGCTATTCTTAGCATGATATTCGGAAATATCATTGCTATTACTCAAACAAGCATGAAACGTATGCTTGCATATTCGTCCATAGGTCAAATC
                              K  R  M  A  S  S  S  G  N  P  S  Y  S  *  H  D  I  R  K  Y  H  C  Y  Y  S  N  K  H  E  T  Y  A  C  I  F  V  H  R  S  N
Lathraea squamaria            AACGAATGGCATCTTCTTCTGGAAATCCTAGCTATTCTTAGCATGATATTCGGAAATATCATTGCTATTACTCAAACAAGCATGAAACGTATGCTTGCATATTCGTCCATAGGTCAAATC
                              K  R  M  A  S  S  S  G  N  P  S  Y  S  *  H  D  I  R  K  Y  H  C  Y  Y  S  N  K  H  E  T  Y  A  C  I  F  V  H  R  S  N
Bartsia_inaequalis            AACGAATGGCATCTTCTTCTGGAAATCCTAGCTATTCTTAGCATGATATTCGGAAATATCATTGCTATTACTCAAACAAGCATGAAACGTATGCTTGCATATTCGTCCATAGGTCAAATA
                               N  E  W  H  L  L  L  E  I  L  A  I  L  S  M  I  F  G  N  I  I  A  I  T  Q  T  S  M  K  R  M  L  A  Y  S  S  I  G  Q  I

                                    1690      1700      1710      1720      1730      1740      1750      1760      1770      1780      1790      1800
                              ....|....|....|....|....|....|....|....|....|....|....|....|....|....|....|....|....|....|....|....|....|....|....|....|
L.squamaria minor_sequence    GGATATGTAATTATTGGAATAATTGTTGGAGACTCAAATGATGGATATGCAAGCATGATAACTTATATGCTGTTCTATATCTCCATGAATCTAGGAACTTTTGCTTGCATTGTATTATTT
                              R  I  C  N  Y  W  N  N  C  W  R  L  K  *  W  I  C  K  H  D  N  L  Y  A  V  L  Y  L  H  E  S  R  N  F  C  L  H  C  I  I
Lathraea squamaria            GGATATGTAATTATTGGAATAATTGTTGGAGACTCAAATGATGGATATGCAAGCATGATAACTTATATGCTGTTCTATATCTCCATGAATCTAGGAACTTTTGCTTGCATTGTATTATTT
                              R  I  C  N  Y  W  N  N  C  W  R  L  K  *  W  I  C  K  H  D  N  L  Y  A  V  L  Y  L  H  E  S  R  N  F  C  L  H  C  I  I
Bartsia_inaequalis            GGATATGTAATTATTGGAATAATTGTTGGAGACTCAAATGATGGATATGCAAGCATGATAACTTATATGCTGTTCTATATCTCCATGAATCTAGGAACTTTTGCTTGCATTGTATTATTT
                               G  Y  V  I  I  G  I  I  V  G  D  S  N  D  G  Y  A  S  M  I  T  Y  M  L  F  Y  I  S  M  N  L  G  T  F  A  C  I  V  L  F

                                    1810      1820      1830      1840      1850      1860      1870      1880      1890      1900      1910      1920
                              ....|....|....|....|....|....|....|....|....|....|....|....|....|....|....|....|....|....|....|....|....|....|....|....|
L.squamaria minor_sequence    GGTCTACGTACCGGAACTGATAACATTCGAGATTATGCAGGATTATACACGAAAGATCCTTTTTTGGCTCTCTCTTTAGCCCTATGTCTCTTATCCCTAGGAGGTCTTCCTCCACTAGCA
                              W  S  T  Y  R  N  *  *  H  S  R  L  C  R  I  I  H  E  R  S  F  F  G  S  L  F  S  P  M  S  L  I  P  R  R  S  S  S  T  S
Lathraea squamaria            GGTCTACGTACCGGAACTGATAACATTCGAGATTATGCAGGATTATACACGAAAGATCCTTTTTTGGCTCTCTCTTTAGCCCTATGTCTCTTATCCCTAGGAGGTCTTCCTCCACTAGCA
                              W  S  T  Y  R  N  *  *  H  S  R  L  C  R  I  I  H  E  R  S  F  F  G  S  L  F  S  P  M  S  L  I  P  R  R  S  S  S  T  S
Bartsia_inaequalis            GGTCTACGTACCGGAACTGATAACATTCGAGATTATGCAGGATTATACACGAAAGATCCTTTTTTGGCTCTCTCTTTAGCCCTATGTCTCTTATCCCTAGGAGGTCTTCCTCCACTAGCA
                               G  L  R  T  G  T  D  N  I  R  D  Y  A  G  L  Y  T  K  D  P  F  L  A  L  S  L  A  L  C  L  L  S  L  G  G  L  P  P  L  A

                                    1930      1940      1950      1960      1970      1980      1990      2000      2010      2020      2030      2040
                              ....|....|....|....|....|....|....|....|....|....|....|....|....|....|....|....|....|....|....|....|....|....|....|....|
L.squamaria minor_sequence    GGTTTTTTCGGAAAACTCTATTTATTCTGGTGTGGATGGCGGGCAGGCGTATATTCCTTGGTTTTAATAGGACTCCTTACAAGCGTTCTTTCTATCTACTATTATCTAAAAATAATCAAG
                              R  F  F  R  K  T  L  F  I  L  V  W  M  A  G  R  R  I  F  L  G  F  N  R  T  P  Y  K  R  S  F  Y  L  L  L  S  K  N  N  Q
Lathraea squamaria            GGTTTTTTCGGAAAACTCTATTTATTCTGGTGTGGATGGCGGGCAGGCGTATATTCCTTGGTTTTAATAGGACTCCTTACAAGCGTTCTTTCTATCTACTATTATCTAAAAATAATCAAG
                              R  F  F  R  K  T  L  F  I  L  V  W  M  A  G  R  R  I  F  L  G  F  N  R  T  P  Y  K  R  S  F  Y  L  L  L  S  K  N  N  Q
Bartsia_inaequalis            GGTTTTTTCGGAAAACTCTATTTATTCTGGTGTGGATGGCGGGCAGGCGTATATTCCTTGGTTTTAATAGGACTCCTTACAAGCGTTCTTTCTATCTACTATTATCTAAAAATAATCAAG
                               G  F  F  G  K  L  Y  L  F  W  C  G  W  R  A  G  V  Y  S  L  V  L  I  G  L  L  T  S  V  L  S  I  Y  Y  Y  L  K  I  I  K

                                    2050      2060      2070      2080      2090      2100      2110      2120      2130      2140      2150      2160
                              ....|....|....|....|....|....|....|....|....|....|....|....|....|....|....|....|....|....|....|....|....|....|....|....|
L.squamaria minor_sequence    TTATTAATGACTGGACGAAACCAAGAAATCACCCCTCACGTGCGAAATTATAGAAGATCTCCTTTAAGATCAAACAATTCCATCGAATTGAGTATGATTGTATGTGTGATAGCATCTACT
```

```
                              V  I  N  D  W  T  K  P  R  N  H  P  S  R  A  K  L  *  K  I  S  F  K  I  K  Q  F  H  R  I  E  Y  D  C  M  C  D  S  I  Y
Lathraea squamaria            TTATTAATGACTGGACGAAACCAAGAAATCACCCCTCACGTGCGAAATTATAGAAGATCTCCTTTAAGATCAAACAATTCCATCGAATTGAGTATGATTGTATGTGTGATAGCATCTACT
                              V  I  N  D  W  T  K  P  R  N  H  P  S  R  A  K  L  *  K  I  S  F  K  I  K  Q  F  H  R  I  E  Y  D  C  M  C  D  S  I  Y
Bartsia_inaequalis            TTATTAATGACTGGACGAAACCAAGAAATCACCCCTCACGTGCGAAATTATAGAAGATCTCCTTTAAGATCAAACAATTCCATCGAATTGAGTATGATTGTATGTGTGATAGCATCTACT
                               L  L  M  T  G  R  N  Q  E  I  T  P  H  V  R  N  Y  R  R  S  P  L  R  S  N  N  S  I  E  L  S  M  I  V  C  V  I  A  S  T

                                      2170      2180      2190      2200      2210      2220
                              ....|....|....|....|....|....|....|....|....|....|....|....|
L.squamaria minor_sequence    ATACCAGGAATATCAATGAACCCGATTATTGCAATTGCTCaGGATACCCTT~~~TTTTAG
                              Y  T  R  N  I  N  E  P  D  Y  C  N  C  S  G  Y  P     F  L
Lathraea squamaria            ATACCAGGAATATCAATGAACCCGATTATTGCAATTGCTCGGGATACCCTTT~~ATTTAG
                              Y  T  R  N  I  N  E  P  D  Y  C  N  C  S  G  Y  P  L     F
Bartsia_inaequalis            ATACCAGGAATATCAATGAACCCGATTATTGCAATTGCTCAGGATACCCTT~~~TTTTAG
                               I  P  G  I  S  M  N  P  I  I  A  I  A  Q  D  T  L     F  *
```

---

psaA

```
                                      10        20        30        40        50        60        70        80        90       100       110       120
                              ....|....|....|....|....|....|....|....|....|....|....|....|....|....|....|....|....|....|....|....|....|....|....|....|
L.squamaria minor sequence    ~~~~~~~~~~~~~~~~~~~~~~~~~~~GTAAAAATTTTGGTGGATAAGGATCCCGTAAAAACTTCTTTCGAGGAATGGGCCAAACCGGGTCATTTCTCAAGAACAATAGCTAAAGGACCT
                                                         V  K  I  L  V  D  K  D  P  V  K  T  S  F  E  E  W  A  K  P  G  H  F  S  R  T  I  A  K  G  P
Lathraea squamaria            ~~~~~~~~~~~~~~~~~~~~~~~~~~~GTAAAAATTTTGGTGGATAAGGATCCCGTAAAAACTTCTTTCGAGGAATGGGCCAAACCGGGTCATTTCTCAAGAACAATAGCTAAAGGACCT
                                                         V  K  I  L  V  D  K  D  P  V  K  T  S  F  E  E  W  A  K  P  G  H  F  S  R  T  I  A  K  G  P
Bartsia inaequalis            ATGATTATTCGTTCGCCGGAACCAGAAGTAAAAATTTTGGTGGATAAGGATCCCGTAAAAACTTCTTTCGAGGAATGGGCCAAACCGGGCCATTTCTCAAGAACAATAGCTAAAGGGCCT
                               M  I  I  R  S  P  E  P  E  V  K  I  L  V  D  K  D  P  V  K  T  S  F  E  E  W  A  K  P  G  H  F  S  R  T  I  A  K  G  P

                                     130       140       150       160       170       180       190       200       210       220       230       240
                              ....|....|....|....|....|....|....|....|....|....|....|....|....|....|....|....|....|....|....|....|....|....|....|....|
L.squamaria minor sequence    GAGACTACCACTTGG~~GATCTGGAACCTACATGCTGATGCTCACGATTTTGATAGCCATACTAGTGATTTGGAGGAGATCTCTCGAAAAGTATTTAGTGCCCATTTTGGTCAACTCTCT
                               E  T  T  T  W     D  L  E  P  T  C  *  C  S  R  F  *  *  P  Y  *  *  F  G  G  D  L  S  K  S  I  *  C  P  F  W  S  T  L
Lathraea squamaria            GAGACTACCACTTGG~~GATCTGGAACCTACATGCTGATGCTCACGATTTTGATAGCCATACTAGTGATTTGGAGGAGATCTCTCGAAAAGTATTTAGTGCCCATTTTGGTCAACTCTCT
                               E  T  T  T  W     D  L  E  P  T  C  *  C  S  R  F  *  *  P  Y  *  *  F  G  G  D  L  S  K  S  I  *  C  P  F  W  S  T  L
Bartsia inaequalis            GAGACTACCACTTGG~~~ATCTGGAACCTACATGCCGATGCTCACGATTTTGATAGCCATACTAGTGATTTGGAGGAGATCTCTCGAAAAGTATTTAGTGCCCATTTCGGTCAACTCTCC
                               E  T  T  T  W      I  W  N  L  H  A  D  A  H  D  F  D  S  H  T  S  D  L  E  E  I  S  R  K  V  F  S  A  H  F  G  Q  L  S

                                     250       260       270       280       290       300       310       320       330       340       350       360
                              ....|....|....|....|....|....|....|....|....|....|....|....|....|....|....|....|....|....|....|....|....|....|....|....|
L.squamaria minor sequence    ATCATCTTTCTTTGGCTGAGCGGCATGTATTTCCATGGTGTTCGTTTTTCCAATTATGAAGCGTGGCTAAGTGATCCAACTCACATTGGGCCAAGTGCCCAGGTGGTTTGGCCAATAGTG
                              Y  H  L  S  L  A  E  R  H  V  F  P  W  C  S  F  F  Q  L  *  S  V  A  K  *  S  N  S  H  W  A  K  C  P  G  G  L  A  N  S
Lathraea squamaria            ATCATCTTTCTTTGGCTGAGCGGCATGTATTTCCATGGTGTTCGTTTTTCCAATTATGAAGCGTGGCTAAGTGATCCAACTCACATTGGGCCAAGTGCCCAGGTGGTTTGGCCAATAGTG
                              Y  H  L  S  L  A  E  R  H  V  F  P  W  C  S  F  F  Q  L  *  S  V  A  K  *  S  N  S  H  W  A  K  C  P  G  G  L  A  N  S
Bartsia inaequalis            ATCATCTTTCTTTGGCTAAGCGGCATGTATTTCCACGGTGCTCGTTTTTCCAATTATGAAGCGTGGCTAAGTGATCCAACTCACATTGGGCCAAGTGCCCAGGTGGTTTGGCCAATAGTG
                               I  I  F  L  W  L  S  G  M  Y  F  H  G  A  R  F  S  N  Y  E  A  W  L  S  D  P  T  H  I  G  P  S  A  Q  V  V  W  P  I  V

                                     370       380       390       400       410       420       430       440       450       460       470       480
                              ....|....|....|....|....|....|....|....|....|....|....|....|....|....|....|....|....|....|....|....|....|....|....|....|
L.squamaria minor sequence    GGCCAAGAAATATTGAACGGTGATGTGGGCGGGGGGTTCCGAGGAATACAAATAACCTCTGGTTTTTTT~~~CAGATTTGGCGAGCATCTGGAATAACTAGTGAATTACAGCTCTATTGT
                              G  P  R  N  I  E  R  *  C  G  R  G  V  P  R  N  T  N  N  L  W  F  F     S  D  L  A  S  I  W  N  N  *  *  I  T  A  L  L
Lathraea squamaria            GGCCAAGAAATATTGAACGGTGATGTGGGCGGGGGGTTCCGAGGAATACAAATAACCTCTGGTTTTTTTTT~CAGATTTGGCGAGCATCTGGAATAACTAGTGAATTACAGCTCTATTGT
                              G  P  R  N  I  E  R  *  C  G  R  G  V  P  R  N  T  N  N  L  W  F  F  F  Q  I  W  R  A  S  G  I  T  S  E  L  Q  L  Y  C
Bartsia inaequalis            GGCCAAGAAATATTGAACGGTGATGTGGGCGGGGGGTTCCGAGGAATACAAATAACCTCTGGTTTTTTT~~~CAGATTTGGCGAGCATCTGGAATAACTAGTGAATTACAGCTCTATTGT
                               G  Q  E  I  L  N  G  D  V  G  G  G  F  R  G  I  Q  I  T  S  G  F  F     Q  I  W  R  A  S  G  I  T  S  E  L  Q  L  Y  C

                                     490       500       510       520       530       540       550       560       570       580       590       600
                              ....|....|....|....|....|....|....|....|....|....|....|....|....|....|....|....|....|....|....|....|....|....|....|....|
L.squamaria minor sequence    ACCGCAATTGGTGCATTGGTCTTTGTAGCCTTAATGCTTTTTACTGGTTGGTTTCATTATCATAAAGCGGCCCCAAAATAGGCTTGGTTTCAAGATGTAGAATCTATGTTAAATCACCAT
```

```
                             Y  R  N  W  C  I  G  L  C  S  L  N  A  F  Y  W  L  V  S  L  S  *  S  G  P  K  I  G  L  V  S  R  C  R  I  Y  V  K  S  P
Lathraea squamaria           ACCGCAATTGGTGCATTGGTCTTTGTAGCCTTAATGCTTTTTACTGGTTGGTTTCATTATCATAAAGCGGCCCCAAAATAGGCTTGGTTTCAAGATGTAGAATCTATGTTAAATCACCAT
                              T  A  I  G  A  L  V  F  V  A  L  M  L  F  T  G  W  F  H  Y  H  K  A  A  P  K  *  A  W  F  Q  D  V  E  S  M  L  N  H  H
Bartsia inaequalis           ACTGCAATTGGTGCATTGGTCTTTGCAGCCTTAATGCTTTTTGCTGGTTGGTTTCATTATCATAAAGCGGCCCCAAAATTGGCTTGGTTTCAAGATGTAGAATCTATGTTGAATCACCAT
                              T  A  I  G  A  L  V  F  A  A  L  M  L  F  A  G  W  F  H  Y  H  K  A  A  P  K  L  A  W  F  Q  D  V  E  S  M  L  N  H  H

                                     610       620       630       640       650       660       670       680       690       700       710       720
                             ....|....|....|....|....|....|....|....|....|....|....|....|....|....|....|....|....|....|....|....|....|....|....|....|
L.squamaria minor sequence   TTAGCGGGGTTATTAGGACTTGGGTCTCTCTCTTGGGCgGGGCATCAGGTGCATGTATCTTTACCGATTAACCAATTTCTAAACGCTGGAGTAGATCCGAAAGAGATACCGCTGCCTCAT
                             F  S  G  V  I  R  T  W  V  S  L  L  G  G  A  S  G  A  C  I  F  T  D  *  P  I  S  K  R  W  S  R  S  E  R  D  T  A  A  S
Lathraea squamaria           TTAGCGGGGTTATTAGGACTTGGGTCTCTCTCTTGGGCAGGGCATCAGGTGCATGTATCTTTACCGATTAACCAATTTCTAAACGCTGGAGTAGATCCGAAAGAGATACCGCTGCCTCAT
                              L  A  G  L  L  G  L  G  S  L  S  W  A  G  H  Q  V  H  V  S  L  P  I  N  Q  F  L  N  A  G  V  D  P  K  E  I  P  L  P  H
Bartsia inaequalis           TTAGCGGGGCTACTAGGACTTGGGTCTCTCTCTTGGGCGGGGCATCAGGTGCATGTATCTTTACCGATTAACCAATTTCTAAACGCTGGAGTAGATCCGAAAGAGATACCGCTGCCTCAT
                              L  A  G  L  L  G  L  G  S  L  S  W  A  G  H  Q  V  H  V  S  L  P  I  N  Q  F  L  N  A  G  V  D  P  K  E  I  P  L  P  H

                                     730       740       750       760       770       780       790       800       810       820       830       840
                             ....|....|....|....|....|....|....|....|....|....|....|....|....|....|....|....|....|....|....|....|....|....|....|....|
L.squamaria minor sequence   GAATTTATCTTGAATCGCGATCTTTTGGCGCAACTCTATCCAAGTTTTGCCGAgGGAGCAACCCCATTTTTCACCTTGAATTGGTCAAAATATGCGGAATTgaTTACTTTTCGTGGAGGA
                             *  I  Y  L  E  S  R  S  F  G  A  T  L  S  K  F  C  R  G  S  N  P  I  F  H  L  E  L  V  K  I  C  G  I  D  Y  F  S  W  R
Lathraea squamaria           GAATTTATCTTGAATCGCGATCTTTTGGCGCAACTCTATCCAAGTTTTGCCGAAGGAGCAACCCCATTTTTCACCTTGAATTGGTCAAAATATGCGGAATTTCTTACTTTTCGTGGAGGA
                              E  F  I  L  N  R  D  L  L  A  Q  L  Y  P  S  F  A  E  G  A  T  P  F  F  T  L  N  W  S  K  Y  A  E  F  L  T  F  R  G  G
Bartsia inaequalis           GAATTTATCTTGAATCGCGATCTTTTGGCGCAACTCTATCCAAGTTTTGCCGAGGGAGCAACCCCGTTTTTCACCTTGAATTGGTCAAAATATGCGGAATTTCTTACTTTTCGTGGAGGA
                              E  F  I  L  N  R  D  L  L  A  Q  L  Y  P  S  F  A  E  G  A  T  P  F  F  T  L  N  W  S  K  Y  A  E  F  L  T  F  R  G  G

                                     850       860       870       880       890       900       910       920       930       940       950       960
                             ....|....|....|....|....|....|....|....|....|....|....|....|....|....|....|....|....|....|....|....|....|....|....|....|
L.squamaria minor sequence   TTAGATCCAGTaACTGGGGGTCTGTGGTTGACCGATATTGCACACCATCATTTAGCTATTGCTATTCTCTTCCTGATAGCGGGTCACATGTATAGGACCAACTGGGGCATTGGTCATGGC
                             I  R  S  S  N  W  G  S  V  V  D  R  Y  C  T  P  S  F  S  Y  C  Y  S  L  P  D  S  G  S  H  V  *  D  Q  L  G  H  W  S  W
Lathraea squamaria           TTAGATCCAGTGACTGGGGGTCTGTGGTTGACCGATATTGCACACCATCATTTAGCTATTGCTATTCTCTTCCTGATAGCGGGTCACATGTATAGGACCAACTGGGGCATTGGTCATGGC
                              L  D  P  V  T  G  G  L  W  L  T  D  I  A  H  H  H  L  A  I  A  I  L  F  L  I  A  G  H  M  Y  R  T  N  W  G  I  G  H  G
Bartsia inaequalis           TTAGATCCAGTAACTGGAGGTCTGTGGTTGACCGATATTGCACACCATCATTTAGCTATTGCTATTCTATTCCTGATAGCGGGTCACATGTATAGGACCAACTGGGGTATTGGGCATGGC
                              L  D  P  V  T  G  G  L  W  L  T  D  I  A  H  H  H  L  A  I  A  I  L  F  L  I  A  G  H  M  Y  R  T  N  W  G  I  G  H  G

                                     970       980       990      1000      1010      1020      1030      1040      1050      1060      1070      1080
                             ....|....|....|....|....|....|....|....|....|....|....|....|....|....|....|....|....|....|....|....|....|....|....|....|
L.squamaria minor sequence   CTAAAAAATATTTTAGAAGCGCATAAAGGCCTA~~~~~~~~~~~~~~~~~~~~~~~~~~~TACGAAATCTTAACAACGTCATGGCATGCTCAATTATCTCTTAACCTAGCCATGTTAGGA
                             P  K  K  Y  F  R  S  A  *  R  P                             I  R  N  L  N  N  V  M  A  C  S  I  I  S  *  P  S  H  V  R
Lathraea squamaria           CTAAAAAATATTTTAGAAGCGCATAAAGGCCTA~~~~~~~~~~~~~~~~~~~~~~~~~~~TACGAAATCTTAACAACGTCATGGCATGCTCAATTATCTCTTAACCTAGCCATGTTAGGA
                              L  K  N  I  L  E  A  H  K  G  L                             Y  E  I  L  T  T  S  W  H  A  Q  L  S  L  N  L  A  M  L  G
Bartsia inaequalis           CTAAAAGATATTTTAGAAGCGCATAAAGGCCCATTTACAGGCCAGGGTCATAAAGGCCTATACGAAATCTTAACAACGTCATGGCATGCTCAATTATCTCTTAACCTAGCCATGTTAGGA
                              L  K  D  I  L  E  A  H  K  G  P  F  T  G  Q  G  H  K  G  L  Y  E  I  L  T  T  S  W  H  A  Q  L  S  L  N  L  A  M  L  G

                                    1090      1100      1110      1120      1130      1140      1150      1160      1170      1180      1190      1200
                             ....|....|....|....|....|....|....|....|....|....|....|....|....|....|....|....|....|....|....|....|....|....|....|....|
L.squamaria minor sequence   TCTTTAACCattgtTGTAgCCCATCATATGTATTCCAtGCCCCCTTATCCATATCTAGCTACTGACTATGCTACACAATTgTCATTGTTCACACATCACATGTGGATTGGTGGATTTCTC
                             I  F  N  H  C  C  S  P  S  Y  V  F  H  A  P  L  S  I  S  S  Y  *  L  C  Y  T  I  V  I  V  H  T  S  H  V  D  W  W  I  S
Lathraea squamaria           TCTTTAACC~~~~~TGTAACCCATCATATGTATTCCACGCCCCCTTATCCATATCTAGCTACTGACTATGCTACACAATTATCATTGTTCACACATCACATGTGGATTGGTGGATTTCTC
                              S  L  T        C  N  P  S  Y  V  F  H  A  P  L  S  I  S  S  Y  *  L  C  Y  T  I  I  I  V  H  T  S  H  V  D  W  W  I  S
Bartsia inaequalis           TCTTTAACCATTGTTGTAGCCCATCATATGTATTCCATGCCCCCTTATCCATATCTAGCTACTGACTATGCTACACAATTGTCATTGTTCACACATCACATGTGGATTGGTGGATTTCTC
                              S  L  T  I  V  V  A  H  H  M  Y  S  M  P  P  Y  P  Y  L  A  T  D  Y  A  T  Q  L  S  L  F  T  H  H  M  W  I  G  G  F  L

                                    1210      1220      1230      1240      1250      1260      1270      1280      1290      1300      1310      1320
                             ....|....|....|....|....|....|....|....|....|....|....|....|....|....|....|....|....|....|....|....|....|....|....|....|
L.squamaria minor sequence   ATAGTTGGTgCTGCTGCGCATGCAGCCATTTTTATGGTAAGAGATTATGATCCAACTACTCGATACAACGATCTATTAGATCGTGTCCTTAGGCATCGTGATGCAATAATATCACATCTc
                             H  S  W  C  C  C  A  C  S  H  F  Y  G  K  R  L  *  S  N  Y  S  I  Q  R  S  I  R  S  C  P  *  A  S  *  C  N  N  I  T  S
Lathraea squamaria           ATAGTTGGTACTGCTGCGCATGCAGCCATTTTTATGGTAAGAGATTATGATCCAACTACTCGATACAACGATCTATTAGATCGTGTCCTTAGGCATCGTGATGCAATAATATCACATCTT
                             H  S  W  Y  C  C  A  C  S  H  F  Y  G  K  R  L  *  S  N  Y  S  I  Q  R  S  I  R  S  C  P  *  A  S  *  C  N  N  I  T  S
Bartsia inaequalis           ATAGTTGGTGCTGCTGCGCACGCAGCTATTTTTATGGTAAGAGATTATGATCCAACTACTCGATATAACGATCTATTAGATCGTGTCCTTAGGCATCGTGATGCAATAATATCACATCTC
```

```
                              I V G A A A H A A I F M V R D Y D P T T R Y N D L L D R V L R H R D A I I S H L

                                    1330      1340      1350      1360      1370      1380      1390      1400      1410      1420      1430      1440
                              ....|....|....|....|....|....|....|....|....|....|....|....|....|....|....|....|....|....|....|....|....|....|....|....|
L.squamaria minor sequence    AACTGGgCATGTATATTTCTGGGCTTTCACAGTTTTGGTTTGTATATTCATAATGATACCATGAGCGCCTTAGGGCGTCCTCAAGATATGTTTTCCGATACCaCTATACAATTACAACCc
                              Q L G M Y I S G L S Q F W F V Y S * * Y H E R L R A S S R Y V F R Y H Y T I T T
Lathraea squamaria            AACTGGACATGTATATTTCTGGGCTTTCACAGTTTTGGTTTGTATATTCATAATGATACCATGAGCGCCTTAGGGCGTCCTCAAGATATGTTTTCCGATACCGCTATACAATTACAACCT
                              * L D M Y I S G L S Q F W F V Y S * * Y H E R L R A S S R Y V F R Y R Y T I T T
Bartsia inaequalis            AACTGGGCATGTATATTTCTGGGCTTTCACAGTTTTGGTTTGTATATTCATAATGATACCATGAGCGCCTTAGGGCGTCCTCAAGATATGTTTTCCGATACCGCTATACAATTACAACCC
                               N W A C I F L G F H S F G L Y I H N D T M S A L G R P Q D M F S D T A I Q L Q P

                                    1450      1460      1470      1480      1490      1500      1510      1520      1530      1540      1550      1560
                              ....|....|....|....|....|....|....|....|....|....|....|....|....|....|....|....|....|....|....|....|....|....|....|....|
L.squamaria minor sequence    GTTTTTGCTCAATGGATACAAAATACTCACGCCTTAGCTCCCGGTGCAACGGCtcctggtgcaacggcAAGCaCCAGTTTAacctgGGGGGGTGGTGATTTAGTAGCAGTGGGCgGCAAA
                              R F C S M D T K Y S R L S S R C N G S W C N G K H Q F N L G G W * F S S S G R Q
Lathraea squamaria            GTTTTTGCTCAATGGATACAAAATACTCACGCCTTAGCTCCCGGTGCAACGGC~~~~~~~~~~~~~~~~AAGCCCCAGTTTA~~~~~GGGGGGTGGTGATTTAGTAGCAGTGGGCAGCAAA
                              C F C S M D T K Y S R L S S R C N G                   K P Q F     R G V V I * * Q W A A K
Bartsia inaequalis            GTTTTTGCTCAATGGATACAAAATACCCATGCCTTAGCCCCCGGTGCAACGGCTCCTGGTGCAACGGCAAGCACCAGTTTAACCTGGGGGGGTGGTGATTTAGTAGCAGTGGGCGGCAAA
                               V F A Q W I Q N T H A L A P G A T A P G A T A S T S L T W G G G D L V A V G G K

                                    1570      1580      1590      1600      1610      1620      1630      1640      1650      1660      1670      1680
                              ....|....|....|....|....|....|....|....|....|....|....|....|....|....|....|....|....|....|....|....|....|....|....|....|
L.squamaria minor sequence    GTGGCTTTGTTACCTATTCCGTTAGGAACGGCGGATTTTTTgGTACATCATATCCATGCATTTACGATTCATgTCACAGTTTTGATACTCCtGAAAGGTGTTCTATTTGCTCGTAGCTCC
                              S G F V T Y S V R N G G F F G T S Y P C I Y D S C H S F D T P E R C S I C S * L
Lathraea squamaria            GTGGCTTTGTTACCTATTCCGTTAGGAACGGCGGATTTTTTAGTACATCATATCCATGCATTTACGATTCATATCACAGTTTTGATACTCCGGAAAGGTGTTCTATTTGCTCGTAGCTCC
                               W L C Y L F R * E R R I F * Y I I S M H L R F I S Q F * Y S G K V F Y L L V A P
Bartsia inaequalis            GTGGCTTTGTTACCTATTCCGTTAGGAACGGCGGATTTTTTGGTACATCATATCCATGCATTTACGATTCATGTCACAGTTTTGATACTCCTGAAAGGTGTTCTATTTGCTCGTAGCTCC
                               V A L L P I P L G T A D F L V H H I H A F T I H V T V L I L L K G V L F A R S S

                                    1690      1700      1710      1720      1730      1740      1750      1760      1770      1780      1790      1800
                              ....|....|....|....|....|....|....|....|....|....|....|....|....|....|....|....|....|....|....|....|....|....|....|....|
L.squamaria minor sequence    TGTTTGATACCGGATAAAGCAAATCTTGGTTTTCGTTTTCCTTGT~~~~~~GATGGACCTGGAAGAGGGGGTACATGTCAAGTATCGGCTTGGGATCATGTCTTCTTAGGACTA~~~TtC
                              L F D T G * S K S W F S F S L    * W T W K R G Y M S S I G L G S C L L R T   I
Lathraea squamaria            TGTTTGATACCGGATAAAGCAAATCTTGGTTTTCGTTTTCCTTGTGATGT~GATGGACCTGGAAGAGGGGGTACATGTCAAGTATCGGCTTGGGATCATGTCTTCTTAGGACTAT~ATC
                               V * Y R I K Q I L V F V F L V M * W T W K R G Y M S S I G L G S C L L R T I   S
Bartsia inaequalis            CGTTTGATACCGGATAAAGCAAATCTTGGTTTTCGTTTTCCTTGT~~~~~~GATGGACCCGGAAGAGGGGGTACATGTCAAGTATCAGCTTGGGATCATGTCTTCTTAGGACTA~~~TTC
                               R L I P D K A N L G F R F P C     D G P G R G G T C Q V S A W D H V F L G L   F

                                    1810      1820      1830      1840      1850      1860      1870      1880      1890      1900      1910      1920
                              ....|....|....|....|....|....|....|....|....|....|....|....|....|....|....|....|....|....|....|....|....|....|....|....|
L.squamaria minor sequence    TGGATGTACAATTCGATTTCGGTAGTAATATTCCATTTCAGTTGGAAAATGCAGTCAGATGTTTGGGGTAGTATAAGTGATCaAGGGGTAGTAACTCATATCACGGGAGGAAACTTCGCG
                              L D V Q F D F G S N I P F Q L E N A V R C L G * Y K * S R G S N S Y H G R K L R
Lathraea squamaria            TGGATGTACAATTCGATTTCGGTAGTAATATTCCATTTCAGTTGGAAAATGCAGTCAGATGTTTGGGGTAGTATAAGTGATCGAGGGGTAGTAACTCATATCACGGGAGGAAACTTCGCG
                               G C T I R F R * * Y S I S V G K C S Q M F G V V * V I E G * * L I S R E E T S R
Bartsia inaequalis            TGGATGTACAATTCTATTTCGGTAGTAATATTCCATTTCAGTTGGAAAATGCAGTCAGATGTTTGGGGTAGTATAAGTGATCAAGGGATAGTAACTCATATCACGGGAGGAAACTTCGCG
                               W M Y N S I S V V I F H F S W K M Q S D V W G S I S D Q G I V T H I T G G N F A

                                    1930      1940      1950      1960      1970      1980      1990      2000      2010      2020      2030      2040
                              ....|....|....|....|....|....|....|....|....|....|....|....|....|....|....|....|....|....|....|....|....|....|....|....|
L.squamaria minor sequence    CAGAGTTCTATTACTATTAATGGGTGGCTTCGGGATTTaTTATGGGCACAGGCATCCCAGGTAATTCAGTCTTATGGTTCTTCATTATCTGCATATGGACTTTTTTTCCTAGGCGCTCAT
                              A E F Y Y Y * W V A S G F I M G T G I P G N S V L W F F I I C I W T F F P R R S
Lathraea squamaria            CAGAGTTCTATTACTATTAATGGGTGGCTTCGGGATTTCTTATGGGCACAGGCATCCCAGGTAATTCAGTCTTATGGTTCTTCATTATCTGCATATGGACTTTTTTTCCTAGGCGCTCAT
                               R V L L L L M G G F G I S Y G H R H P R * F S L M V L H Y L H M D F F S * A L I
Bartsia inaequalis            CAGAGTTCTATTACTATTAATGGGTGGCTTCGGGATTTCTTATGGGCACAGGCATCCCAGGTAATTCAGTCTTATGGTTCTTCATTATCTGCATATGGACTTTTTTTCCTAGGCGCTCAT
                               Q S S I T I N G W L R D F L W A Q A S Q V I Q S Y G S S L S A Y G L F F L G A H

                                    2050      2060      2070      2080      2090      2100      2110      2120      2130      2140      2150      2160
                              ....|....|....|....|....|....|....|....|....|....|....|....|....|....|....|....|....|....|....|....|....|....|....|....|
```

```
L.squamaria minor sequence  TTTGTATGGGCTTTTAGTTTAATGTTTCTATTCATCGGGCGTGGTTATTGGCAAGAACTTATTGAATCCATCGTTTGGGCTCATAATAAATTAAAAGTTGCTCCTGCTACTCAGCCGAGA
                             F  C  M  G  F  *  F  N  V  S  I  H  R  A  W  L  L  A  R  T  Y  *  I  H  R  L  G  S  *  *  I  K  S  C  S  C  Y  S  A  E
Lathraea squamaria          TTTGTATGGGCTTTTAGTTTAATGTTTCTATTCATCGGGCGTGGTTATTGGCAAGAACTTATTGAATCCATCGTTTGGGCTCATAATAAATTAAAAGTTGCTCCTGCTACTCAGCCGAGA
                              L  Y  G  L  L  V  *  C  F  Y  S  S  G  V  V  I  G  K  N  L  L  N  P  S  F  G  L  I  I  N  *  K  L  L  L  L  L  S  R  E
Bartsia inaequalis          TTTGTATGGGCTTTTAGTTTAATGTTTCTGTTCAGTGGGCGTGGTTATTGGCAAGAACTTATTGAATCCATCGTTTGGGCTCATAATAAATTAAAAGTTGCTCCTGCTACTCAGCCGAGA
                               F  V  W  A  F  S  L  M  F  L  F  S  G  R  G  Y  W  Q  E  L  I  E  S  I  V  W  A  H  N  K  L  K  V  A  P  A  T  Q  P  R

                                      2170      2180      2190      2200      2210      2220      2230      2240      2250      2260
                            ....|....|....|....|....|....|....|....|....|....|....|....|....|....|....|....|....|....|....|....|....|...
L.squamaria minor sequence  GCCTTGAGTATTGTACAAGGACGTGCTGTAGGAGTAACCCATTACCTTCTGGGTGGAATTGCCACAACATGGGCGTTCTTCTTAGCAAGAATTATTGCAGTAGGATAA
                            S  L  E  Y  C  T  R  T  C  C  R  S  N  P  L  P  S  G  W  N  C  H  N  M  G  V  L  L  S  K  N  Y  C  S  R  I
Lathraea squamaria          GCCTTGAGTATTGTACAAGGACGTGCTGTAGGAGTAACCCATTACCTTCTGGGTGGAATTGCCACAACATGGGCGTTCTTCTTAGCAAGAATTATTGCAGTAGGATAA
                              P  *  V  L  Y  K  D  V  L  *  E  *  P  I  T  F  W  V  E  L  P  Q  H  G  R  S  S  *  Q  E  L  L  Q  *  D
Bartsia inaequalis          GCCTTGAGTATTGTACAAGGACGTGCTGTAGGAGTAACCCATTACCTTCTGGGTGGAATTGCCACAACATGGGCGTTCTTCTTAGCAAGAATTATTGCAGTAGGATAA
                             A  L  S  I  V  Q  G  R  A  V  G  V  T  H  Y  L  L  G  G  I  A  T  T  W  A  F  F  L  A  R  I  I  A  V  G  *
```

<u>*psaB* partial alignment</u>

```
                                970       980       990      1000      1010      1020      1030      1040      1050      1060      1070      1080
                            ....|....|....|....|....|....|....|....|....|....|....|....|....|....|....|....|....|....|....|....|....|....|....|....|
L.squamaria minor sequence  GGGTCTTTATGATACAATCAATAATTCACTTCATTTTCAATTAGGCCTTGCTCTAGCTTCTTTAGGGGTTATTACCTCCTTGGTAGCTCAACACATGTACTCTTTACCTGCTTATGCATT
                              G  L  Y  D  T  I  N  N  S  L  H  F  Q  L  G  L  A  L  A  S  L  G  V  I  T  S  L  V  A  Q  H  M  Y  S  L  P  A  Y  A  F
Lathraea squamaria          GGGTCTTTATGATACAATCAATAATTCACTTCATTTTCAATTAGGCCTTGCTCTAGCTTCTTTAGGGGTTATTACCTCCTTGGTAGCTCAACACATGTACTCTTTACCTGCTTATGCATT
                              G  L  Y  D  T  I  N  N  S  L  H  F  Q  L  G  L  A  L  A  S  L  G  V  I  T  S  L  V  A  Q  H  M  Y  S  L  P  A  Y  A  F
Bartsia inaequalis          GGGTCTTTATGATACAATCAATAATTCACTTCATTTTCAATTAGGCCTTGCTCTAGCTTCTTTAGGGGTTATTACCTCCTTGGTAGCTCAACACATGTACTCTTTACCTGCTTATGCATT
                              G  L  Y  D  T  I  N  N  S  L  H  F  Q  L  G  L  A  L  A  S  L  G  V  I  T  S  L  V  A  Q  H  M  Y  S  L  P  A  Y  A  F

                                      1090      1100      1110      1120      1130      1140      1150      1160      1170      1180      1190      1200
                            ....|....|....|....|....|....|....|....|....|....|....|....|....|....|....|....|....|....|....|....|....|....|....|....|
L.squamaria minor sequence  CATAGCACAAGACTTTACTACTCAAGCTGCATTATATACTCATCACCAATATATCGCAGgATTCATCATGACAGGAGCTTTTGCTCACGGAGCGATATTTTTCATTAGAGATTATAACCC
                              I  A  Q  D  F  T  T  Q  A  A  L  Y  T  H  H  Q  Y  I  A  G  F  I  M  T  G  A  F  A  H  G  A  I  F  F  I  R  D  Y  N  P
Lathraea squamaria          CATAGCACAAGACTTTACTACTCAAGCTGCATTATATACTCATCACCAATATATCGCAGAATTCATCATGACAGGAGCTTTTGCTCACGGAGCGATATTTTTCATTAGAGATTATAACCC
                              I  A  Q  D  F  T  T  Q  A  A  L  Y  T  H  H  Q  Y  I  A  E  F  I  M  T  G  A  F  A  H  G  A  I  F  F  I  R  D  Y  N  P
Bartsia inaequalis          CATAGCACAAGACTTTACTACTCAAGCTGCATTATATACTCATCACCAATATATCGCAGGATTCATCATGACAGGAGCTTTTGCTCACGGAGCGATATTTTTCATTAGAGATTATAACCC
                              I  A  Q  D  F  T  T  Q  A  A  L  Y  T  H  H  Q  Y  I  A  G  F  I  M  T  G  A  F  A  H  G  A  I  F  F  I  R  D  Y  N  P

                                      1210      1220      1230      1240      1250      1260      1270      1280      1290      1300      1310      1320
                            ....|....|....|....|....|....|....|....|....|....|....|....|....|....|....|....|....|....|....|....|....|....|....|....|
L.squamaria minor sequence  GGAACAAAATGAAGATAATGTATTGGCAAGAATGTTAGACcATAAGGAAGCTATTATATCTcATTTAAGTTGGGCCAGCCTCTTTCTAGGATTCCATACCcTGGGACTTTATGTTCATAA
                              E  Q  N  E  D  N  V  L  A  R  M  L  D  H  K  E  A  I  I  S  H  L  S  W  A  S  L  F  L  G  F  H  T  L  G  L  Y  V  H  N
Lathraea squamaria          GGAACAAAATGAAGATAATGTATTGGCAAGAATGTTAGACTATAAGGAAGCTATTATATCTTATTTAAGTTGGGCCAGCCTCTTTCTAGGATTCCATACCTTGGGACTTTATGTTCATAA
                              E  Q  N  E  D  N  V  L  A  R  M  L  D  Y  K  E  A  I  I  S  Y  L  S  W  A  S  L  F  L  G  F  H  T  L  G  L  Y  V  H  N
Bartsia inaequalis          GGAACAAAATGAAGATAATGTATTGGCAAGAATGTTAGACCATAAGGAAGCTATTATATCTCATTTAAGTTGGGCCAGCCTCTTTCTAGGATTCCATACCCTGGGACTTTATGTTCATAA
                              E  Q  N  E  D  N  V  L  A  R  M  L  D  H  K  E  A  I  I  S  H  L  S  W  A  S  L  F  L  G  F  H  T  L  G  L  Y  V  H  N

                                      1330      1340      1350      1360      1370      1380      1390      1400      1410      1420      1430      1440
                            ....|....|....|....|....|....|....|....|....|....|....|....|....|....|....|....|....|....|....|....|....|....|....|....|
L.squamaria minor sequence  TGATGTCATGCTTGCTTTtGGTACTCCGGAAAAtagAATTtGGATCGAACCTATATTTGCTCAATGGATACAATCGGCTCATGGTAAAACTTCATATGGCTTCGATGTAtTTTTcTCTTC
                              D  V  M  L  A  F  G  T  P  E  N  R  I  W  I  E  P  I  F  A  Q  W  I  Q  S  A  H  G  K  T  S  Y  G  F  D  V  F  F  S  S
Lathraea squamaria          TGATGTCATGCTTGCTTTCGGTACTCCGGAAAAGCAAATTAGGATCGAACCTATATTTGCTCAATGGATACAATCGGCTCATGGTAAAACTTCATATGGCTTCGATGTACTTTTATCTTC
                              D  V  M  L  A  F  G  T  P  E  K  Q  I  R  I  E  P  I  F  A  Q  W  I  Q  S  A  H  G  K  T  S  Y  G  F  D  V  L  L  S  S
Bartsia inaequalis          TGATGTCATGCTTGCTTTTGGTACTCCGGAAAAGCAAATTTTGATCGAACCTATATTTGCTCAATGGATACAATCGGCTCATGGTAAAACTGCATATGGCTTCGATGTATTGTTATCTTC
                              D  V  M  L  A  F  G  T  P  E  K  Q  I  L  I  E  P  I  F  A  Q  W  I  Q  S  A  H  G  K  T  A  Y  G  F  D  V  L  L  S  S

                                      1450      1460      1470      1480      1490      1500      1510      1520      1530      1540      1550      1560
                            ....|....|....|....|....|....|....|....|....|....|....|....|....|....|....|....|....|....|....|....|....|....|....|....|
```

```
L.squamaria minor sequence  AACGAGCGGcCCGGCATTCAATGCGGGTCGAAGCATCTGGTTGcctggttgGTTAAATGCTATTAATGAAAATACTAATTCATTATTCTTAACAATAGGTCCtGGAGACTTTTTaGTTCA
                              T  S  G  P  A  F  N  A  G  R  S  I  W  L  P  G  W  L  N  A  I  N  E  N  T  N  S  L  F  L  T  I  G  P  G  D  F  L  V  H
Lathraea squamaria          AACGAGCGGGCCGGCATTCAATGCGGGTCGAAGCATCTGGTTG~~~~~~~~GTTAAATGCTATTAATGAAAATACTAATTCATTATTCTTAACAATAGGTCCCGGAGACTTTTTGGTTCA
                              T  S  G  P  A  F  N  A  G  R  S  I  W  L           V  K  C  Y  *  *  K  Y  *  F  I  I  L  N  N  R  S  R  R  L  F  G  S
Bartsia inaequalis          AACGAGCGGCCCGGCATTCAATGCGGGTCGAAGCATCTGGTTGCCTGGTTGGTTAAATGCTATTAATGAAAATACTAATTCATTATTCTTAACAATAGGTCCCGGAGACTTTTTAGTTCA
                              T  S  G  P  A  F  N  A  G  R  S  I  W  L  P  G  W  L  N  A  I  N  E  N  T  N  S  L  F  L  T  I  G  P  G  D  F  L  V  H

                                    1570      1580      1590      1600      1610      1620      1630      1640      1650      1660      1670      1680
                            ....|....|....|....|....|....|....|....|....|....|....|....|....|....|....|....|....|....|....|....|....|....|....|....|
L.squamaria minor sequence  TCATGCTATTGCTCTGGGTTTACATACAACTACATTGATCTTAGTAAAAGGTGCTTTAGATGCACGCGGTTCAAAGTTAATGCCAGATAAAAAGGATTTCggttatAGTTTTCCGTGCGA
                              H  A  I  A  L  G  L  H  T  T  T  L  I  L  V  K  G  A  L  D  A  R  G  S  K  L  M  P  D  K  K  D  F  G  Y  S  F  P  C  D
Lathraea squamaria          TCATGCTATTGCTCTGGGTTTACATACAACTACATTGATCTTAGTAAAAGGTGCTTTAGATGCACGCGGTTCAAAGTTAATGCCAGATAAAAAGGATTTC~~~~~~AGTTTTCCGTGCGA
                             S  C  Y  C  S  G  F  T  Y  N  Y  I  D  L  S  K  R  C  F  R  C  T  R  F  K  V  N  A  R  *  K  G  F        Q  F  S  V  R
Bartsia inaequalis          TCATGCTATTGCTCTGGGTTTACATACAACTACATTGATCTTAGTAAAAGGTGCTTTAGATGCACGCGGTTCAAAGTTAATGCCAGATAAAAAGGATTTCGGTTATAGTTTTCCGTGCGA
                              H  A  I  A  L  G  L  H  T  T  T  L  I  L  V  K  G  A  L  D  A  R  G  S  K  L  M  P  D  K  K  D  F  G  Y  S  F  P  C  D

                                    1690      1700      1710      1720      1730      1740      1750      1760      1770      1780      1790      1800
                            ....|....|....|....|....|....|....|....|....|....|....|....|....|....|....|....|....|....|....|....|....|....|....|....|
L.squamaria minor sequence  TGGCCCGGGACGGGGG~GGTACTTGTGATATTTCGGCATGGGATGCATTTTATTTGGCAGTTTTTTGGATGTTAAATACTATTGGATGGGTTACTTTTTATTGGCATTGGAAGCACATCA
                              G  P  G  R  G     G  T  C  D  I  S  A  W  D  A  F  Y  L  A  V  F  W  M  L  N  T  I  G  W  V  T  F  Y  W  H  W  K  H  I
Lathraea squamaria          TGGCCCGGGACGGGGGGGGTACTTGTGATATTTCGGCATGGGATGCATTTTATTTGGCAGTTTTTTGGATGTTAAATACTATTGGATGGGTTACTTTTTATTGGCATTGGAAGCACATCA
                             W  P  G  T  G  G  V  L  V  I  F  R  H  G  M  H  F  I  W  Q  F  F  G  C  *  I  L  L  D  G  L  L  F  I  G  I  G  S  T  S
Bartsia inaequalis          TGGCCCGGGACGGGGG~GGTACTTGTGATATTTCGGCATGGGATGCATTTTATTTGGCAGTTTTTTGGATGTTAAATACTATTGGATGGGTTACTTTTTATTGGCATTGGAAGCACATCA
                              G  P  G  R  G     G  T  C  D  I  S  A  W  D  A  F  Y  L  A  V  F  W  M  L  N  T  I  G  W  V  T  F  Y  W  H  W  K  H  I

                                    1810      1820      1830      1840      1850      1860      1870      1880      1890      1900      1910      1920
                            ....|....|....|....|....|....|....|....|....|....|....|....|....|....|....|....|....|....|....|....|....|....|....|....|
L.squamaria minor sequence  CATTATGGCAGGGTAACGTTTCACAGTTTAATGAATCCTCCACTTATTTGATGGGCTGGCTAAGAGATTATTTATgGTTAAACTCTTCACAACTTatcaatgGATATAACCCTTTTGGTA
                            T  L  W  Q  G  N  V  S  Q  F  N  E  S  S  T  Y  L  M  G  W  L  R  D  Y  L  W  L  N  S  S  Q  L  I  N  G  Y  N  P  F  G
Lathraea squamaria          CATTATGGCAGGGTAACGTTTCACAGTTTAATGAATCCTCCACTTATTTGATGGGCTGGCTAAGAGATTATTTATAGTTAAACTCTTCACAACT~~~~~~~~GATATAACCCTTTTGGTA
                             H  Y  G  R  V  T  F  H  S  L  M  N  P  P  L  I  *  W  A  G  *  E  I  I  Y  S  *  T  L  H  N        *  Y  N  P  F  G
Bartsia inaequalis          CATTATGGCAGGGTAACGTTTCACAGTTTAATGAATCCTCCACTTATTTGATGGGCTGGCTCAGAGATTATTTATGGTTAAACTCTTCACAACTTATCAATGGATATAACCCTTTTGGTA
                            T  L  W  Q  G  N  V  S  Q  F  N  E  S  S  T  Y  L  M  G  W  L  R  D  Y  L  W  L  N  S  S  Q  L  I  N  G  Y  N  P  F  G

                                    1930      1940      1950      1960      1970      1980      1990      2000      2010      2020      2030      2040
                            ....|....|....|....|....|....|....|....|....|....|....|....|....|....|....|....|....|....|....|....|....|....|....|....|
L.squamaria minor sequence  TGAATAGTTTATCGGTCTGGGCGtGGATGTTTTTATTTGGACATCTTGTTTGGGCTACTGGATTTATGTTCTTAATCTCCTGGCGtGGATATTGGCAgGAATTGATTGAAACTTTAGCAT
                            M  N  S  L  S  V  W  A  W  M  F  L  F  G  H  L  V  W  A  T  G  F  M  F  L  I  S  W  R  G  Y  W  Q  E  L  I  E  T  L  A
Lathraea squamaria          TGAATAGTTTATCGGTCTGGGCGCGGATGTTTTTATTTGGACATCTTGTTTGGGCTACTGGATTTATGTTCTTAATCTCCTGGCGCGGATATTGGCAAGAATTGATTGAAACTTTAGCAT
                            M  N  S  L  S  V  W  A  R  M  F  L  F  G  H  L  V  W  A  T  G  F  M  F  L  I  S  W  R  G  Y  W  Q  E  L  I  E  T  L  A
Bartsia inaequalis          TGAATAGTTTATCGGTCTGGGCGTGGATGTTTTTATTTGGACATCTTGTTTGGGCTACTGGATTTATGTTCTTAATCTCCTGGCGTGGATATTGGCAGGAATTGATTGAAACTTTAGCAT
                            M  N  S  L  S  V  W  A  W  M  F  L  F  G  H  L  V  W  A  T  G  F  M  F  L  I  S  W  R  G  Y  W  Q  E  L  I  E  T  L  A

                                    2050      2060      2070      2080      2090      2100      2110      2120      2130      2140      2150      2160
                            ....|....|....|....|....|....|....|....|....|....|....|....|....|....|....|....|....|....|....|....|....|....|....|....|
L.squamaria minor sequence  GGGCTCATGAACGCACACCTTTGGCCAATTTGATTCGATGGAGAGATAAA~CCTGTGGCCCTTTCCATTGTGCAAGCAAGATTGGTTgGATTAGCCCACTTTTCTGTAGGTTATATATTC
                            W  A  H  E  R  T  P  L  A  N  L  I  R  W  R  D  K     P  V  A  L  S  I  V  Q  A  R  L  V  G  L  A  H  F  S  V  G  Y  I  F
Lathraea squamaria          GGGCTCATGAACGCACACCTTTGGCCAATTTGATTCGATGGAGAGATAAAACCTGTGGCCCTTTCCATTGTGCAAGCAAGATTGGTTAGATTAGCCCACTTTTCTGTAGGTTATATATTC
                            W  A  H  E  R  T  P  L  A  N  L  I  R  W  R  D  K  T  C  G  P  F  H  C  A  S  K  I  G  *  I  S  P  L  F  C  R  L  Y  I
Bartsia inaequalis          GGGCTCATGAACGCACACCTTTGGCCAATTTGATTCGATGGAGAGATAAA~CCTGTGGCTCTTTCCATTGTGCAAGCAAGATTGGTTGGATTAGCTCACTTTTCTGTAGGTTATATATTC
                            W  A  H  E  R  T  P  L  A  N  L  I  R  W  R  D  K     P  V  A  L  S  I  V  Q  A  R  L  V  G  L  A  H  F  S  V  G  Y  I  F

                                    2170      2180      2190      2200
                            ....|....|....|....|....|....|....|....|...
L.squamaria minor sequence  ACTTATGCAGCTTTCTTGATTGCCTCgACATCGGGAAAATTTGGCTcA
                             T  Y  A  A  F  L  I  A  S  T  S  G  K  F  G  S
Lathraea squamaria          ACTTATGCAGCTTTCTTGATTGCCTCCACATCGGGAAAATTTGGCTAA
                            H  L  C  S  F  L  D  C  L  H  I  G  K  I  W  L
```

```
Bartsia inaequalis          ACTTATGCAGCTTTCTTGATTGCCTCGACATCGGGAAAATTTGGC~~~
                             T  Y  A  A  F  L  I  A  S  T  S  G  K  F  G 
```

---

ycf2

```
                                    10        20        30        40        50        60        70        80        90       100       110       120
                            ....|....|....|....|....|....|....|....|....|....|....|....|....|....|....|....|....|....|....|....|....|....|....|....|
L.squamaria minor sequence  ATGAAAGAACATCCATTCAAATCCTGGATTTTCGAATTGAGAGAGATCAAGAATTCTCACTATTTCTTAGATTCATGGACTCAATTCAATTCCGTAGGGTCTTTCATTCACATTTTTTTC
                             M  K  E  H  P  F  K  S  W  I  F  E  L  R  E  I  K  N  S  H  Y  F  L  D  S  W  T  Q  F  N  S  V  G  S  F  I  H  I  F  F 
Lathraea squamaria          ........................................................................................................................
                             M  K  E  H  P  F  K  S  W  I  F  E  L  R  E  I  K  N  S  H  Y  F  L  D  S  W  T  Q  F  N  S  V  G  S  F  I  H  I  F  F 
Bartsia inaequalis          ........................................................................................................................
                             M  K  E  H  P  F  K  S  W  I  F  E  L  R  E  I  K  N  S  H  Y  F  L  D  S  W  T  Q  F  N  S  V  G  S  F  I  H  I  F  F 

                                   130       140       150       160       170       180       190       200       210       220       230       240
                            ....|....|....|....|....|....|....|....|....|....|....|....|....|....|....|....|....|....|....|....|....|....|....|....|
L.squamaria minor sequence  CACCAAGAACGTTTTCTAAAACTCCTTGACCCCCGAATTTTGAGTATCCTACTTTCATGCAATTCACAGGGTTCAACAAGCAATCGATATTTCACGATCAAGGGTGTAATACTCTTTGTA
                             H  Q  E  R  F  L  K  L  L  D  P  R  I  L  S  I  L  L  S  C  N  S  Q  G  S  T  S  N  R  Y  F  T  I  K  G  V  I  L  F  V 
Lathraea squamaria          ........................................................................................................................
                             H  Q  E  R  F  L  K  L  L  D  P  R  I  L  S  I  L  L  S  C  N  S  Q  G  S  T  S  N  R  Y  F  T  I  K  G  V  I  L  F  V 
Bartsia inaequalis          .........................................................C..............................................................
                             H  Q  E  R  F  L  K  L  L  D  P  R  I  L  S  I  L  L  S  R  N  S  Q  G  S  T  S  N  R  Y  F  T  I  K  G  V  I  L  F  V 

                                   250       260       270       280       290       300       310       320       330       340       350       360
                            ....|....|....|....|....|....|....|....|....|....|....|....|....|....|....|....|....|....|....|....|....|....|....|....|
L.squamaria minor sequence  GTAGCGGTCCTTATA~~~~~~TATCGTATTAACAATCGAAATATGGTCGAAAGAAAAAATCTCTATTGGAGGGGGCTTCTTCCTATGAATTCCATTGGACCCAGAAATGATACATTGGAA
                             V  A  V  L  I        Y  R  I  N  N  R  N  M  V  E  R  K  N  L  Y  W  R  G  L  L  P  M  N  S  I  G  P  R  N  D  T  L  E 
Lathraea squamaria          ...............~~~~~~...................................................................................................
                             V  A  V  L  I        Y  R  I  N  N  R  N  M  V  E  R  K  N  L  Y  W  R  G  L  L  P  M  N  S  I  G  P  R  N  D  T  L  E 
Bartsia inaequalis          ...............TTTATA..............C....................................................................................
                             V  A  V  L  I  F  I  Y  R  I  N  N  R  N  M  V  E  R  K  N  L  Y  W  R  G  L  L  P  M  N  S  I  G  P  R  N  D  T  L  E 

                                   370       380       390       400       410       420       430       440       450       460       470       480
                            ....|....|....|....|....|....|....|....|....|....|....|....|....|....|....|....|....|....|....|....|....|....|....|....|
L.squamaria minor sequence  GAATCGGTTGGGTCTTCCAATATCAATAGGTTGATTGTTTCGCTCCTCTATCTTCCAAAAGGAAAAAAGATCTCGGAGAGTTGTTTCCTGAATCCGAAAGAGAGTACTTGGATTCTCCCA
                             E  S  V  G  S  S  N  I  N  R  L  I  V  S  L  L  Y  L  P  K  G  K  K  I  S  E  S  C  F  L  N  P  K  E  S  T  W  I  L  P 
Lathraea squamaria          ........................................................................................................................
                             E  S  V  G  S  S  N  I  N  R  L  I  V  S  L  L  Y  L  P  K  G  K  K  I  S  E  S  C  F  L  N  P  K  E  S  T  W  I  L  P 
Bartsia inaequalis          ..........................................................................T.............................................
                             E  S  V  G  S  S  N  I  N  R  L  I  V  S  L  L  Y  L  P  K  G  K  K  I  S  E  S  C  F  L  N  P  K  E  S  T  W  I  L  P 

                                   490       500       510       520       530       540       550       560       570       580       590       600
                            ....|....|....|....|....|....|....|....|....|....|....|....|....|....|....|....|....|....|....|....|....|....|....|....|
L.squamaria minor sequence  ATAACTCAAAAGTGTAGCATGCCTGAATCTAACTGGGGTTCGCGGTGGTGGAGGAACTGGAGCGGAAAAAGGAGGGATTCTAGTTGTAAGATATCTAATGAAACCGTCGCTGGAATTGAG
                             I  T  Q  K  C  S  M  P  E  S  N  W  G  S  R  W  W  R  N  W  S  G  K  R  R  D  S  S  C  K  I  S  N  E  T  V  A  G  I  E 
Lathraea squamaria          ........................................................................................................................
                             I  T  Q  K  C  S  M  P  E  S  N  W  G  S  R  W  W  R  N  W  S  G  K  R  R  D  S  S  C  K  I  S  N  E  T  V  A  G  I  E 
Bartsia inaequalis          ......A...............................................................A.................................................
                             I  T  K  K  C  S  M  P  E  S  N  W  G  S  R  W  W  R  N  W  S  G  K  K  R  D  S  S  C  K  I  S  N  E  T  V  A  G  I  E 

                                   610       620       630       640       650       660       670       680       690       700       710       720
                            ....|....|....|....|....|....|....|....|....|....|....|....|....|....|....|....|....|....|....|....|....|....|....|....|
L.squamaria minor sequence  ATCTTATTCAACGAGAAAGATATCAAATATCTGGAGTTTCTTTTTGTATATTATATGGATGATCCGATCCGCAAGGACCGTGATTGGGAATTGTTTGATTGTCTTTCTCTGAGGAAGAGG
                             I  L  F  N  E  K  D  I  K  Y  L  E  F  L  F  V  Y  Y  M  D  D  P  I  R  K  D  R  D  W  E  L  F  D  C  L  S  L  R  K  R 
Lathraea squamaria          ........................................................................................................................
                             I  L  F  N  E  K  D  I  K  Y  L  E  F  L  F  V  Y  Y  M  D  D  P  I  R  K  D  R  D  W  E  L  F  D  C  L  S  L  R  K  R 
```

```
Bartsia inaequalis           .......................................A................................................................................
                              I  L  F  N  E  K  D  I  K  Y  L  E  F  I  F  V  Y  Y  M  D  D  P  I  R  K  D  R  D  W  E  L  F  D  C  L  S  L  R  K  R

                                  730       740       750       760       770       780       790       800       810       820       830       840
                             ....|....|....|....|....|....|....|....|....|....|....|....|....|....|....|....|....|....|....|....|....|....|....|....|
L.squamaria minor sequence   CGAAATAGAATCAACTTGAATTCGGGATCGCTATTCGAAATCTTAGTGAAACACTGGATTTCTTATCTCATGTCTGCTTTTCGTGAAAAAATACCAATTGAAGTGGAGGGTTTCTTCAAA
                              R  N  R  I  N  L  N  S  G  S  L  F  E  I  L  V  K  H  W  I  S  Y  L  M  S  A  F  R  E  K  I  P  I  E  V  E  G  F  F  K
Lathraea squamaria           ........................................................................................................................
                              R  N  R  I  N  L  N  S  G  S  L  F  E  I  L  V  K  H  W  I  S  Y  L  M  S  A  F  R  E  K  I  P  I  E  V  E  G  F  F  K
Bartsia inaequalis           ........................................................................................................................
                              R  N  R  I  N  L  N  S  G  S  L  F  E  I  L  V  K  H  W  I  S  Y  L  M  S  A  F  R  E  K  I  P  I  E  V  E  G  F  F  K

                                  850       860       870       880       890       900       910       920       930       940       950       960
                             ....|....|....|....|....|....|....|....|....|....|....|....|....|....|....|....|....|....|....|....|....|....|....|....|
L.squamaria minor sequence   CAACAAAGGGCTGGGTCAACTATTCAATCAAATGATATTGAGCATGTTTCTCATCTCTTCTCGAGAAACAAGTGGGCTATTTCTTTGCAAAATTGTGCTCAATTTCATATGTGGCAATTT
                              Q  Q  R  A  G  S  T  I  Q  S  N  D  I  E  H  V  S  H  L  F  S  R  N  K  W  A  I  S  L  Q  N  C  A  Q  F  H  M  W  Q  F
Lathraea squamaria           ........................................................................................................................
                              Q  Q  R  A  G  S  T  I  Q  S  N  D  I  E  H  V  S  H  L  F  S  R  N  K  W  A  I  S  L  Q  N  C  A  Q  F  H  M  W  Q  F
Bartsia inaequalis           ........................................................................................................................
                              Q  Q  R  A  G  S  T  I  Q  S  N  D  I  E  H  V  S  H  L  F  S  R  N  K  W  A  I  S  L  Q  N  C  A  Q  F  H  M  W  Q  F

                                  970       980       990      1000      1010      1020      1030      1040      1050      1060      1070      1080
                             ....|....|....|....|....|....|....|....|....|....|....|....|....|....|....|....|....|....|....|....|....|....|....|....|
L.squamaria minor sequence   CGCCAGGATCTCTTCGTTAGTTGGGGGAAGAATCCGCACGAATCGGATTTTTTGAGGAACATATCGAGAGAGAATTTGATTTGGTTAGACAATGTGTGGTTGGTAAACAAGGATCCGTTT
                              R  Q  D  L  F  V  S  W  G  K  N  P  H  E  S  D  F  L  R  N  I  S  R  E  N  L  I  W  L  D  N  V  W  L  V  N  K  D  P  F
Lathraea squamaria           ........................................................................................................................
                              R  Q  D  L  F  V  S  W  G  K  N  P  H  E  S  D  F  L  R  N  I  S  R  E  N  L  I  W  L  D  N  V  W  L  V  N  K  D  P  F
Bartsia inaequalis           ........................................................................................................................
                              R  Q  D  L  F  V  S  W  G  K  N  P  H  E  S  D  F  L  R  N  I  S  R  E  N  L  I  W  L  D  N  V  W  L  V  N  K  D  P  F

                                 1090      1100      1110      1120      1130      1140      1150      1160      1170      1180      1190      1200
                             ....|....|....|....|....|....|....|....|....|....|....|....|....|....|....|....|....|....|....|....|....|....|....|....|
L.squamaria minor sequence   TTTAGAAAGGTACGGAATGTATCGTCAAATATTCAATATGATTCCACAAGATCCAGTTTCGTTCAAGTAACGGATTCTAGCCAACCGAAAGGATCTTCTGATCAATCCAGAGATCATTTG
                              F  R  K  V  R  N  V  S  S  N  I  Q  Y  D  S  T  R  S  S  F  V  Q  V  T  D  S  S  Q  P  K  G  S  S  D  Q  S  R  D  H  L
Lathraea squamaria           ........................................................................................................................
                              F  R  K  V  R  N  V  S  S  N  I  Q  Y  D  S  T  R  S  S  F  V  Q  V  T  D  S  S  Q  P  K  G  S  S  D  Q  S  R  D  H  L
Bartsia inaequalis           ...........................T............................................................................................
                              F  R  K  V  R  N  V  S  S  Y  I  Q  Y  D  S  T  R  S  S  F  V  Q  V  T  D  S  S  Q  P  K  G  S  S  D  Q  S  R  D  H  L

                                 1210      1220      1230      1240      1250      1260      1270      1280      1290      1300      1310      1320
                             ....|....|....|....|....|....|....|....|....|....|....|....|....|....|....|....|....|....|....|....|....|....|....|....|
L.squamaria minor sequence   GATTCCATTAGTAATGAGGATTCGGAATATCACACATTGATCAATCAAAGAGAGATTCAACCACTAAAAGAAAGATCGATTCTTTGGGATCCTTCCTTTCTTCAAACGGAAGGAACAGAG
                              D  S  I  S  N  E  D  S  E  Y  H  T  L  I  N  Q  R  E  I  Q  P  L  K  E  R  S  I  L  W  D  P  S  F  L  Q  T  E  G  T  E
Lathraea squamaria           ........................................................................................................................
                              D  S  I  S  N  E  D  S  E  Y  H  T  L  I  N  Q  R  E  I  Q  P  L  K  E  R  S  I  L  W  D  P  S  F  L  Q  T  E  G  T  E
Bartsia inaequalis           .................................G......................................................................................
                              D  S  I  S  N  E  D  S  E  Y  H  A  L  I  N  Q  R  E  I  Q  P  L  K  E  R  S  I  L  W  D  P  S  F  L  Q  T  E  G  T  E

                                 1330      1340      1350      1360      1370      1380      1390      1400      1410      1420      1430      1440
                             ....|....|....|....|....|....|....|....|....|....|....|....|....|....|....|....|....|....|....|....|....|....|....|....|
L.squamaria minor sequence   ATAGAATCCGACCGATTCCCGAAATCCCTTTCTGGATATTCCTCAATGTCCCGGCTATTCACGGAACGTGAGAAGCAGATGATTAATCATCTGCTTCCGGAAGAAATCGAAGAATTTCTT
                              I  E  S  D  R  F  P  K  S  L  S  G  Y  S  S  M  S  R  L  F  T  E  R  E  K  Q  M  I  N  H  L  L  P  E  E  I  E  E  F  L
Lathraea squamaria           ........................................................................................................................
                              I  E  S  D  R  F  P  K  S  L  S  G  Y  S  S  M  S  R  L  F  T  E  R  E  K  Q  M  I  N  H  L  L  P  E  E  I  E  E  F  L
Bartsia inaequalis           ........................................................................................................................
                              I  E  S  D  R  F  P  K  S  L  S  G  Y  S  S  M  S  R  L  F  T  E  R  E  K  Q  M  I  N  H  L  L  P  E  E  I  E  E  F  L

                                 1450      1460      1470      1480      1490      1500      1510      1520      1530      1540      1550      1560
```

```
                            ....|....|....|....|....|....|....|....|....|....|....|....|....|....|....|....|....|....|....|....|....|....|....|....|
L.squamaria minor sequence  GGGAATCCTACAAGATCGGTTCGTTCTTTTTTCTCTGATAGATGGTCAGAACTTCATCTGGGTTCGAATCCTACTGAGAGGTCCACTAGAGATCAGAAATTCTTGAAGAAACAACAAGAT
                             G  N  P  T  R  S  V  R  S  F  F  S  D  R  W  S  E  L  H  L  G  S  N  P  T  E  R  S  T  R  D  Q  K  F  L  K  K  Q  Q  D
Lathraea squamaria          ........................................................................................................................
                             G  N  P  T  R  S  V  R  S  F  F  S  D  R  W  S  E  L  H  L  G  S  N  P  T  E  R  S  T  R  D  Q  K  F  L  K  K  Q  Q  D
Bartsia inaequalis          ......................................A.......................G.........................................................
                             G  N  P  T  R  S  V  R  S  F  F  S  E  R  W  S  E  L  H  L  G  S  N  P  T  E  R  S  T  R  D  Q  K  F  L  K  K  Q  Q  D

                                  1570      1580      1590      1600      1610      1620      1630      1640      1650      1660      1670      1680
                            ....|....|....|....|....|....|....|....|....|....|....|....|....|....|....|....|....|....|....|....|....|....|....|....|
L.squamaria minor sequence  CTTTCGTTTGTCCCTTCCAGGCGATCGGAAAATAAAGAACTGGTTAATATATTCAAGATAATTACGTATTTACAAAATACTGTCTCAATTCATCCCATTTCACCGGGGTGTGATAGGGTT
                             L  S  F  V  P  S  R  R  S  E  N  K  E  L  V  N  I  F  K  I  I  T  Y  L  Q  N  T  V  S  I  H  P  I  S  P  G  C  D  R  V
Lathraea squamaria          ........................................................................................................................
                             L  S  F  V  P  S  R  R  S  E  N  K  E  L  V  N  I  F  K  I  I  T  Y  L  Q  N  T  V  S  I  H  P  I  S  P  G  C  D  R  V
Bartsia inaequalis          ........................................................................................................................
                             L  S  F  V  P  S  R  R  S  E  N  K  E  L  V  N  I  F  K  I  I  T  Y  L  Q  N  T  V  S  I  H  P  I  S  P  G  C  D  R  V

                                  1690      1700      1710      1720      1730      1740      1750      1760      1770      1780      1790      1800
                            ....|....|....|....|....|....|....|....|....|....|....|....|....|....|....|....|....|....|....|....|....|....|....|....|
L.squamaria minor sequence  CTGAAGGATGAACCGGATATGGACAGTTCCAATAAGATGTCATTCTTGAACAAAAATCCATTTTTTGATTTATTTCATCTATTCCATGACCGGAACAGGGGAGGATACACCTTACACAAC
                             L  K  D  E  P  D  M  D  S  S  N  K  M  S  F  L  N  K  N  P  F  F  D  L  F  H  L  F  H  D  R  N  R  G  G  Y  T  L  H  N
Lathraea squamaria          ........................................................................................................................
                             L  K  D  E  P  D  M  D  S  S  N  K  M  S  F  L  N  K  N  P  F  F  D  L  F  H  L  F  H  D  R  N  R  G  G  Y  T  L  H  N
Bartsia inaequalis          .....................................................................................................................C..
                             L  K  D  E  P  D  M  D  S  S  N  K  M  S  F  L  N  K  N  P  F  F  D  L  F  H  L  F  H  D  R  N  R  G  G  Y  T  L  H  H

                                  1810      1820      1830      1840      1850      1860      1870      1880      1890      1900      1910      1920
                            ....|....|....|....|....|....|....|....|....|....|....|....|....|....|....|....|....|....|....|....|....|....|....|....|
L.squamaria minor sequence  GATTTTGAATCAGAAGAGAGATTTCAAGAAATGGCAGATCTATTCACTCTATCAATAACCGAGCCAGATCTGGTGTATCATAAGGGATTTGCCTTTTCTATT~~~~~~GATTCCTACGGA
                             D  F  E  S  E  E  R  F  Q  E  M  A  D  L  F  T  L  S  I  T  E  P  D  L  V  Y  H  K  G  F  A  F  S  I        D  S  Y  G
Lathraea squamaria          ......................................................................................................~~~~~~............
                             D  F  E  S  E  E  R  F  Q  E  M  A  D  L  F  T  L  S  I  T  E  P  D  L  V  Y  H  K  G  F  A  F  S  I        D  S  Y  G
Bartsia inaequalis          ......................................................................................................TCTATT.......C..A.
                             D  F  E  S  E  E  R  F  Q  E  M  A  D  L  F  T  L  S  I  T  E  P  D  L  V  Y  H  K  G  F  A  F  S  I  S  I  D  S  S  E

                                  1930      1940      1950      1960      1970      1980      1990      2000      2010      2020      2030      2040
                            ....|....|....|....|....|....|....|....|....|....|....|....|....|....|....|....|....|....|....|....|....|....|....|....|
L.squamaria minor sequence  TTGGATCAAAAACAATTCTTGAATGAGGCCAGGGATGAATCGAAAAAGAAATCTTTATTGGTTCTACCTCCTATTTTTTATGAAGAGAATGAATCTTTTTCTCGAAGGATCAGAAAAAAA
                             L  D  Q  K  Q  F  L  N  E  A  R  D  E  S  K  K  K  S  L  L  V  L  P  P  I  F  Y  E  E  N  E  S  F  S  R  R  I  R  K  K
Lathraea squamaria          ........................................................................................................................
                             L  D  Q  K  Q  F  L  N  E  A  R  D  E  S  K  K  K  S  L  L  V  L  P  P  I  F  Y  E  E  N  E  S  F  S  R  R  I  R  K  K
Bartsia inaequalis          ...............................................................................................................C.......G
                             L  D  Q  K  Q  F  L  N  E  A  R  D  E  S  K  K  K  S  L  L  V  L  P  P  I  F  Y  E  E  N  E  S  F  S  R  R  I  R  K  K

                                  2050      2060      2070      2080      2090      2100      2110      2120      2130      2140      2150      2160
                            ....|....|....|....|....|....|....|....|....|....|....|....|....|....|....|....|....|....|....|....|....|....|....|....|
L.squamaria minor sequence  CGGGCCCGGATCTCCTGCGGGAATGATTTGGAAGATCCAAAACCAAAAATGGTGGTATTTGCTAGCAACAACATAATGGAGGCAGTCAATCAATATAGATTGATCCGAAATCTGATTCAA
                             R  A  R  I  S  C  G  N  D  L  E  D  P  K  P  K  M  V  V  F  A  S  N  N  I  M  E  A  V  N  Q  Y  R  L  I  R  N  L  I  Q
Lathraea squamaria          ........................................................................................................................
                             R  A  R  I  S  C  G  N  D  L  E  D  P  K  P  K  M  V  V  F  A  S  N  N  I  M  E  A  V  N  Q  Y  R  L  I  R  N  L  I  Q
Bartsia inaequalis          .......................................................................G................................................
                             R  A  R  I  S  C  G  N  D  L  E  D  P  K  P  K  M  V  V  F  A  S  N  K  I  M  E  A  V  N  Q  Y  R  L  I  R  N  L  I  Q

                                  2170      2180      2190      2200      2210      2220      2230      2240      2250      2260      2270      2280
                            ....|....|....|....|....|....|....|....|....|....|....|....|....|....|....|....|....|....|....|....|....|....|....|....|
L.squamaria minor sequence  ATCCAATATAGTACCTATGGATACATAAGAAATGTATTGAATCGATTCTTTTTAATGAATAGATCCGATCGCAACTTCGAATATGGAATTCAAAGGGATCAAATAGGAAAGGATACTCTG
                             I  Q  Y  S  T  Y  G  Y  I  R  N  V  L  N  R  F  F  L  M  N  R  S  D  R  N  F  E  Y  G  I  Q  R  D  Q  I  G  K  D  T  L
Lathraea squamaria          ........................................................................................................................
```

```
                            I  Q  Y  S  T  Y  G  Y  I  R  N  V  L  N  R  F  F  L  M  N  R  S  D  R  N  F  E  Y  G  I  Q  R  D  Q  I  G  K  D  T  L
Bartsia inaequalis         ........................................................................................................................
                            I  Q  Y  S  T  Y  G  Y  I  R  N  V  L  N  R  F  F  L  M  N  R  S  D  R  N  F  E  Y  G  I  Q  R  D  Q  I  G  K  D  T  L

                                 2290      2300      2310      2320      2330      2340      2350      2360      2370      2380      2390      2400
                           ....|....|....|....|....|....|....|....|....|....|....|....|....|....|....|....|....|....|....|....|....|....|....|....|
L.squamaria minor sequence AATCATAGAACTATAATGAAATATACGATCAACCAACATTTTTCGAATTTGAAAAAGAGTCAGAAGAAATGGTTCGATCCTCTTATCTTTATTTCTCGAACCGAGAGATCCATGAATAGG
                            N  H  R  T  I  M  K  Y  T  I  N  Q  H  F  S  N  L  K  K  S  Q  K  K  W  F  D  P  L  I  F  I  S  R  T  E  R  S  M  N  R
Lathraea squamaria         ........................................................................................................................
                            N  H  R  T  I  M  K  Y  T  I  N  Q  H  F  S  N  L  K  K  S  Q  K  K  W  F  D  P  L  I  F  I  S  R  T  E  R  S  M  N  R
Bartsia inaequalis         .....................................................................................................................C..
                            N  H  R  T  I  M  K  Y  T  I  N  Q  H  F  S  N  L  K  K  S  Q  K  K  W  F  D  P  L  I  F  I  S  R  T  E  R  S  M  N  R

                                 2410      2420      2430      2440      2450      2460      2470      2480      2490      2500      2510      2520
                           ....|....|....|....|....|....|....|....|....|....|....|....|....|....|....|....|....|....|....|....|....|....|....|....|
L.squamaria minor sequence GATCCTGATGCATATAGATACAAATGGTCCAATGGGAGCAAGAATTTTCAGGAACATTTGGACCATTTCGTTTCTGAGCATAAGAGCCGTTTTCAAATAGTGTTCGATCGATTACGTATT
                            D  P  D  A  Y  R  Y  K  W  S  N  G  S  K  N  F  Q  E  H  L  D  H  F  V  S  E  H  K  S  R  F  Q  I  V  F  D  R  L  R  I
Lathraea squamaria         ........................................................................................................................
                            D  P  D  A  Y  R  Y  K  W  S  N  G  S  K  N  F  Q  E  H  L  D  H  F  V  S  E  H  K  S  R  F  Q  I  V  F  D  R  L  R  I
Bartsia inaequalis         ..............................................................A........G................................................
                            D  P  D  A  Y  R  Y  K  W  S  N  G  S  K  N  F  Q  E  H  L  E  H  F  V  S  E  H  K  S  R  F  Q  I  V  F  D  R  L  R  I

                                 2530      2540      2550      2560      2570      2580      2590      2600      2610      2620      2630      2640
                           ....|....|....|....|....|....|....|....|....|....|....|....|....|....|....|....|....|....|....|....|....|....|....|....|
L.squamaria minor sequence AATCAATATTCGATTGATTGGTCTGAGGTTATCGACACAAAAGATTTGTCTAAGCCACTTCGTTTCTTTTTGTCCAAGTCACTTCTTTTTTTGTCCAAGTTGCTTTTCTTTTTGTCTAAC
                            N  Q  Y  S  I  D  W  S  E  V  I  D  T  K  D  L  S  K  P  L  R  F  F  L  S  K  S  L  L  F  L  S  K  L  L  F  F  L  S  N
Lathraea squamaria         ........................................................................................................................
                            N  Q  Y  S  I  D  W  S  E  V  I  D  T  K  D  L  S  K  P  L  R  F  F  L  S  K  S  L  L  F  L  S  K  L  L  F  F  L  S  N
Bartsia inaequalis         ......................................................................................G.................................
                            N  Q  Y  S  I  D  W  S  E  V  I  D  T  K  D  L  S  K  P  L  R  F  F  L  S  K  S  L  L  F  L  S  K  L  L  F  F  L  S  N

                                 2650      2660      2670      2680      2690      2700      2710      2720      2730      2740      2750      2760
                           ....|....|....|....|....|....|....|....|....|....|....|....|....|....|....|....|....|....|....|....|....|....|....|....|
L.squamaria minor sequence TCACTTCCTTTTTTCTGTGTGAGTTTTGGGAATATCCCCATTCATAGGTCCGAGATCTACATCTATGAATTGAAAGGTCCGAATGATCAACTCTGCAATCAGTTGTTAGAATCAATAGGT
                            S  L  P  F  F  C  V  S  F  G  N  I  P  I  H  R  S  E  I  Y  I  Y  E  L  K  G  P  N  D  Q  L  C  N  Q  L  L  E  S  I  G
Lathraea squamaria         ........................................................................................................................
                            S  L  P  F  F  C  V  S  F  G  N  I  P  I  H  R  S  E  I  Y  I  Y  E  L  K  G  P  N  D  Q  L  C  N  Q  L  L  E  S  I  G
Bartsia inaequalis         ..........................C.............................................................................................
                            S  L  P  F  F  C  V  S  F  G  N  I  P  I  H  R  S  E  I  Y  I  Y  E  L  K  G  P  N  D  Q  L  C  N  Q  L  L  E  S  I  G

                                 2770      2780      2790      2800      2810      2820      2830      2840      2850      2860      2870      2880
                           ....|....|....|....|....|....|....|....|....|....|....|....|....|....|....|....|....|....|....|....|....|....|....|....|
L.squamaria minor sequence CTTCAAATTGTTCATTTGAAAAAATGGAAACCCTTCTTATTGGATGATCATGATACGTCCCGAAAATCGAAATTCTTGATCACTGGAGGAAGACCCTTTTTGTTCAATAAGATACCAAAG
                            L  Q  I  V  H  L  K  K  W  K  P  F  L  L  D  D  H  D  T  S  R  K  S  K  F  L  I  T  G  G  R  P  F  L  F  N  K  I  P  K
Lathraea squamaria         ........................................................................................................................
                            L  Q  I  V  H  L  K  K  W  K  P  F  L  L  D  D  H  D  T  S  R  K  S  K  F  L  I  T  G  G  R  P  F  L  F  N  K  I  P  K
Bartsia inaequalis         ..........................................................A.............................................................
                            L  Q  I  V  H  L  K  K  W  K  P  F  L  L  D  D  H  D  T  Y  R  K  S  K  F  L  I  T  G  G  R  P  F  L  F  N  K  I  P  K

                                 2890      2900      2910      2920      2930      2940      2950      2960      2970      2980      2990      3000
                           ....|....|....|....|....|....|....|....|....|....|....|....|....|....|....|....|....|....|....|....|....|....|....|....|
L.squamaria minor sequence TGCATGATTGACTCATTCCATACTAGAAATAATAGCAGGAAATCCTTTGATAACGCGGATTCCTATTTCTCAATGATATTCCACAATCAAGACAATTGGCTGAATCCCGTGAAACCATTT
                            C  M  I  D  S  F  H  T  R  N  N  S  R  K  S  F  D  N  A  D  S  Y  F  S  M  I  F  H  N  Q  D  N  W  L  N  P  V  K  P  F
Lathraea squamaria         ........................................................................................................................
                            C  M  I  D  S  F  H  T  R  N  N  S  R  K  S  F  D  N  A  D  S  Y  F  S  M  I  F  H  N  Q  D  N  W  L  N  P  V  K  P  F
Bartsia inaequalis         .................................C......................................................................................
                            C  M  I  D  S  F  H  T  R  N  N  R  R  K  S  F  D  N  A  D  S  Y  F  S  M  I  F  H  N  Q  D  N  W  L  N  P  V  K  P  F
```

```
                                 3010      3020      3030      3040      3050      3060      3070      3080      3090      3100      3110      3120
                           ....|....|....|....|....|....|....|....|....|....|....|....|....|....|....|....|....|....|....|....|....|....|....|....|
L.squamaria minor sequence CATAGAAGTTCATTGATATCTTCTTTTTATAAAGCAAATCGACTTCGATTCTTGAATAATCCACATCACTTCTGCTTCTATTGTAACACAAGATTCCCCTTTTCTGTGGAAAAGGCCCGT
                            H  R  S  S  L  I  S  S  F  Y  K  A  N  R  L  R  F  L  N  N  P  H  H  F  C  F  Y  C  N  T  R  F  P  F  S  V  E  K  A  R 
Lathraea squamaria         ........................................................................................................................
                            H  R  S  S  L  I  S  S  F  Y  K  A  N  R  L  R  F  L  N  N  P  H  H  F  C  F  Y  C  N  T  R  F  P  F  S  V  E  K  A  R 
Bartsia inaequalis         ........................................................................................................................
                            H  R  S  S  L  I  S  S  F  Y  K  A  N  R  L  R  F  L  N  N  P  H  H  F  C  F  Y  C  N  T  R  F  P  F  S  V  E  K  A  R 

                                 3130      3140      3150      3160      3170      3180      3190      3200      3210      3220      3230      3240
                           ....|....|....|....|....|....|....|....|....|....|....|....|....|....|....|....|....|....|....|....|....|....|....|....|
L.squamaria minor sequence ATCAATAATTATGATTTTACGTATCGACAATTCCTCAATATCTTGTTCATTCGCAACAAAATATTTTCTTTGTGCGTCGGTAAAAAAAAACATGCTTTTGGGGGGAGATATACTATTTCA
                            I  N  N  Y  D  F  T  Y  R  Q  F  L  N  I  L  F  I  R  N  K  I  F  S  L  C  V  G  K  K  K  H  A  F  G  G  R  Y  T  I  S 
Lathraea squamaria         ........................................................................................................................
                            I  N  N  Y  D  F  T  Y  R  Q  F  L  N  I  L  F  I  R  N  K  I  F  S  L  C  V  G  K  K  K  H  A  F  G  G  R  Y  T  I  S 
Bartsia inaequalis         ............................................................................................................G...........
                            I  N  N  Y  D  F  T  Y  R  Q  F  L  N  I  L  F  I  R  N  K  I  F  S  L  C  V  G  K  K  K  H  A  F  G  G  R  D  T  I  S 

                                 3250      3260      3270      3280      3290      3300      3310      3320      3330      3340      3350      3360
                           ....|....|....|....|....|....|....|....|....|....|....|....|....|....|....|....|....|....|....|....|....|....|....|....|
L.squamaria minor sequence CCAATCGAGTCACAGGTATCTAACATATTCATAACTAACGATTTTCCACAAAGTGGTGACGAAACGTATAACTTGTCCAAATCTTTCCATTTTCCAAGTCGATGCGATCCATTCGTTCGT
                            P  I  E  S  Q  V  S  N  I  F  I  T  N  D  F  P  Q  S  G  D  E  T  Y  N  L  S  K  S  F  H  F  P  S  R  C  D  P  F  V  R 
Lathraea squamaria         ........................................................................................................................
                            P  I  E  S  Q  V  S  N  I  F  I  T  N  D  F  P  Q  S  G  D  E  T  Y  N  L  S  K  S  F  H  F  P  S  R  C  D  P  F  V  R 
Bartsia inaequalis         .......................................................................................................A................
                            P  I  E  S  Q  V  S  N  I  F  I  T  N  D  F  P  Q  S  G  D  E  T  Y  N  L  S  K  S  F  H  F  P  S  R  Y  D  P  F  V  R 

                                 3370      3380      3390      3400      3410      3420      3430      3440      3450      3460      3470      3480
                           ....|....|....|....|....|....|....|....|....|....|....|....|....|....|....|....|....|....|....|....|....|....|....|....|
L.squamaria minor sequence AGAACTATTTACTCGATCgCAGACATTTCTGGAACACCTCTAACAGAGGGACAAATAGTCAATTTTGAAAGAACTTATTGTCAACCTCTTTCAGATATGAATCTATCTGATTCAGAAGGG
                            R  T  I  Y  S  I  A  D  I  S  G  T  P  L  T  E  G  Q  I  V  N  F  E  R  T  Y  C  Q  P  L  S  D  M  N  L  S  D  S  E  G 
Lathraea squamaria         ..................A.....................................................................................................
                            R  T  I  Y  S  I  T  D  I  S  G  T  P  L  T  E  G  Q  I  V  N  F  E  R  T  Y  C  Q  P  L  S  D  M  N  L  S  D  S  E  G 
Bartsia inaequalis         .............................................................................................A..........................
                            R  T  I  Y  S  I  A  D  I  S  G  T  P  L  T  E  G  Q  I  V  N  F  E  R  T  Y  C  Q  P  L  S  N  M  N  L  S  D  S  E  G 

                                 3490      3500      3510      3520      3530      3540      3550      3560      3570      3580      3590      3600
                           ....|....|....|....|....|....|....|....|....|....|....|....|....|....|....|....|....|....|....|....|....|....|....|....|
L.squamaria minor sequence AAGAACTTGCATCAGTATCTCAATTCAAACATGGGTTTGATTCACACTCCATGTTCTGAGAAATATTTACCATCCtAAAAGAGGAAAAAACGGAGTCTTTGTCTAAAGAAATGCGTTGAG
                            K  N  L  H  Q  Y  L  N  S  N  M  G  L  I  H  T  P  C  S  E  K  Y  L  P  S  *  K  R  K  K  R  S  L  C  L  K  K  C  V  E 
Lathraea squamaria         ...........................................................................G............................................
                            K  N  L  H  Q  Y  L  N  S  N  M  G  L  I  H  T  P  C  S  E  K  Y  L  P  S  E  K  R  K  K  R  S  L  C  L  K  K  C  V  E 
Bartsia inaequalis         ...........................................................................G............................................
                            K  N  L  H  Q  Y  L  N  S  N  M  G  L  I  H  T  P  C  S  E  K  Y  L  P  S  E  K  R  K  K  R  S  L  C  L  K  K  C  V  E 

                                 3610      3620      3630      3640      3650      3660      3670      3680      3690      3700      3710      3720
                           ....|....|....|....|....|....|....|....|....|....|....|....|....|....|....|....|....|....|....|....|....|....|....|....|
L.squamaria minor sequence AAAGGGCAGATGTATAGAACCTTTCAACGAGATAGTGCTTTTTCAACTCTCTCAAAATGGAATCTATTCCAAACATATATGCCATGGTTCCTTACTTCGACAGGGTACAAATATCTAAAT
                            K  G  Q  M  Y  R  T  F  Q  R  D  S  A  F  S  T  L  S  K  W  N  L  F  Q  T  Y  M  P  W  F  L  T  S  T  G  Y  K  Y  L  N 
Lathraea squamaria         ........................................................................................................................
                            K  G  Q  M  Y  R  T  F  Q  R  D  S  A  F  S  T  L  S  K  W  N  L  F  Q  T  Y  M  P  W  F  L  T  S  T  G  Y  K  Y  L  N 
Bartsia inaequalis         ..................................................A.....................................................................
                            K  G  Q  M  Y  R  T  F  Q  R  D  S  A  F  S  T  L  S  K  W  N  L  F  Q  T  Y  M  P  W  F  L  T  S  T  G  Y  K  Y  L  N 

                                 3730      3740      3750      3760      3770      3780      3790      3800      3810      3820      3830      3840
                           ....|....|....|....|....|....|....|....|....|....|....|....|....|....|....|....|....|....|....|....|....|....|....|....|
L.squamaria minor sequence TTGATATTTTTAGATACTTTTTCAGAACTATTGCCGATACTAAGTAGCAGTCAAAAATTTgTATCCATTTTTCATGATATTATGCATGGATCAGGTATATCATGGCGAATTCTTCAGAAA
                            L  I  F  L  D  T  F  S  E  L  L  P  I  L  S  S  S  Q  K  F  V  S  I  F  H  D  I  M  H  G  S  G  I  S  W  R  I  L  Q  K 
```

```
Lathraea squamaria          ............................................................T...........................................................
                             L  I  F  L  D  T  F  S  E  L  L  P  I  L  S  S  S  Q  K  F  L  S  I  F  H  D  I  M  H  G  S  G  I  S  W  R  I  L  Q  K
Bartsia inaequalis          ........................................................................................................................
                             L  I  F  L  D  T  F  S  E  L  L  P  I  L  S  S  S  Q  K  F  V  S  I  F  H  D  I  M  H  G  S  G  I  S  W  R  I  L  Q  K

                                  3850      3860      3870      3880      3890      3900      3910      3920      3930      3940      3950      3960
                            ....|....|....|....|....|....|....|....|....|....|....|....|....|....|....|....|....|....|....|....|....|....|....|....|
L.squamaria minor sequence  AAATGGTGTCTTCCACAATGGAATCTGATAAGTGAGATTTCGAGTAAGTGTTTACATAATCTTCTTCTGTCCGAAGAAATGATTCATCGAAATAATGAGTCACCATTGATATCGACACAT
                             K  W  C  L  P  Q  W  N  L  I  S  E  I  S  S  K  C  L  H  N  L  L  L  S  E  E  M  I  H  R  N  N  E  S  P  L  I  S  T  H
Lathraea squamaria          ........................................................................................................................
                             K  W  C  L  P  Q  W  N  L  I  S  E  I  S  S  K  C  L  H  N  L  L  L  S  E  E  M  I  H  R  N  N  E  S  P  L  I  S  T  H
Bartsia inaequalis          ........................................................................................................................
                             K  W  C  L  P  Q  W  N  L  I  S  E  I  S  S  K  C  L  H  N  L  L  L  S  E  E  M  I  H  R  N  N  E  S  P  L  I  S  T  H

                                  3970      3980      3990      4000      4010      4020      4030      4040      4050      4060      4070      4080
                            ....|....|....|....|....|....|....|....|....|....|....|....|....|....|....|....|....|....|....|....|....|....|....|....|
L.squamaria minor sequence  CTGAGATCGCCAAATGTTCGGGAGTTCCTCTATTCAATCCTTTTCCTTCTTCTTGTTGCTGGATATCTCGTTCGTACACATCTTCTCTTTGTTTCCCGGGCCTCTAGTGAGTTACAGACA
                             L  R  S  P  N  V  R  E  F  L  Y  S  I  L  F  L  L  L  V  A  G  Y  L  V  R  T  H  L  L  F  V  S  R  A  S  S  E  L  Q  T
Lathraea squamaria          ........................................................................................................................
                             L  R  S  P  N  V  R  E  F  L  Y  S  I  L  F  L  L  L  V  A  G  Y  L  V  R  T  H  L  L  F  V  S  R  A  S  S  E  L  Q  T
Bartsia inaequalis          .....................................................................................................................C..
                             L  R  S  P  N  V  R  E  F  L  Y  S  I  L  F  L  L  L  V  A  G  Y  L  V  R  T  H  L  L  F  V  S  R  A  S  S  E  L  Q  P

                                  4090      4100      4110      4120      4130      4140      4150      4160      4170      4180      4190      4200
                            ....|....|....|....|....|....|....|....|....|....|....|....|....|....|....|....|....|....|....|....|....|....|....|....|
L.squamaria minor sequence  GAGTTCGAAAGGGTCAAATCTTTGATGATTCCATCATCTATGACTGAGTTGCGAAAACTTCTGGATAGGTATCCTACATCTGCACCGAATTCTTTCTGGTTAAAGAATCTCTTTCTAGTT
                             E  F  E  R  V  K  S  L  M  I  P  S  S  M  T  E  L  R  K  L  L  D  R  Y  P  T  S  A  P  N  S  F  W  L  K  N  L  F  L  V
Lathraea squamaria          ........................................................................................................................
                             E  F  E  R  V  K  S  L  M  I  P  S  S  M  T  E  L  R  K  L  L  D  R  Y  P  T  S  A  P  N  S  F  W  L  K  N  L  F  L  V
Bartsia inaequalis          ........................................................................................................................
                             E  F  E  R  V  K  S  L  M  I  P  S  S  M  T  E  L  R  K  L  L  D  R  Y  P  T  S  A  P  N  S  F  W  L  K  N  L  F  L  V

                                  4210      4220      4230      4240      4250      4260      4270      4280      4290      4300      4310      4320
                            ....|....|....|....|....|....|....|....|....|....|....|....|....|....|....|....|....|....|....|....|....|....|....|....|
L.squamaria minor sequence  GCTCTGGAACAATTAGGAGATTCTTTAGAAGAAATACGGGCTTCTGGCGGCAACATGCTTGGTCCCGCTTATGGGGTCAAATCAATACGTTCTAAgAAGAAATATTTGAATATCAATCtc
                             A  L  E  Q  L  G  D  S  L  E  E  I  R  A  S  G  G  N  M  L  G  P  A  Y  G  V  K  S  I  R  S  K  K  K  Y  L  N  I  N  L
Lathraea squamaria          ...............................................................................................A........................
                             A  L  E  Q  L  G  D  S  L  E  E  I  R  A  S  G  G  N  M  L  G  P  A  Y  G  V  K  S  I  R  S  K  K  K  Y  L  N  I  N  L
Bartsia inaequalis          ........................................................................................................................
                             A  L  E  Q  L  G  D  S  L  E  E  I  R  A  S  G  G  N  M  L  G  P  A  Y  G  V  K  S  I  R  S  K  K  K  Y  L  N  I  N  L

                                  4330      4340      4350      4360      4370      4380      4390      4400      4410      4420      4430      4440
                            ....|....|....|....|....|....|....|....|....|....|....|....|....|....|....|....|....|....|....|....|....|....|....|....|
L.squamaria minor sequence  atcgataTCATCGATCTCATACCAAATCCCAcCAATCGAATCACTTTTTCGAGAAATACGAGATATCTAAGTCATACAAGTAAAGAGATCTATTCATTGATAAGAAAAAGAAAAAAcGTG
                             I  D  I  I  D  L  I  P  N  P  T  N  R  I  T  F  S  R  N  T  R  Y  L  S  H  T  S  K  E  I  Y  S  L  I  R  K  R  K  N  V
Lathraea squamaria          ~~~~~~~~~......................T....................................................................................T...
                                      I  D  L  I  P  N  P  I  N  R  I  T  F  S  R  N  T  R  Y  L  S  H  T  S  K  E  I  Y  S  L  I  R  K  R  K  N  V
Bartsia inaequalis          ~~~~~~~~~......................T........................................................................................
                                      I  D  L  I  P  N  P  I  N  R  I  T  F  S  R  N  T  R  Y  L  S  H  T  S  K  E  I  Y  S  L  I  R  K  R  K  N  V

                                  4450      4460      4470      4480      4490      4500      4510      4520      4530      4540      4550      4560
                            ....|....|....|....|....|....|....|....|....|....|....|....|....|....|....|....|....|....|....|....|....|....|....|....|
L.squamaria minor sequence  AATGGGGATTGGATTGATGATAAAATcGAATCCTGGGTCGCGAACAGTGATTCGATTGATGATGAAGAAAGAGACTTCTTGGTTCAGTTCTCCGCC~~~~~~~~~GAAAAAAGGGTTGAT
                             N  G  D  W  I  D  D  K  I  E  S  W  V  A  N  S  D  S  I  D  D  E  E  R  D  F  L  V  Q  F  S  A           E  K  R  V  D
Lathraea squamaria          ..........................A.....................................................................TTACCGACA...............
                             N  G  D  W  I  D  D  K  I  E  S  W  V  A  N  S  D  S  I  D  D  E  E  R  D  F  L  V  Q  F  S  A  L  P  T  E  K  R  V  D
Bartsia inaequalis          ..........................A.....................................................................TTAACGACA...............
                             N  G  D  W  I  D  D  K  I  E  S  W  V  A  N  S  D  S  I  D  D  E  E  R  D  F  L  V  Q  F  S  A  L  T  T  E  K  R  V  D
```

```
                                      4570      4580      4590      4600      4610      4620      4630      4640      4650      4660      4670      4680
                            ....|....|....|....|....|....|....|....|....|....|....|....|....|....|....|....|....|....|....|....|....|....|....|....|
L.squamaria minor sequence  CAAATTCTATTGAGTCTGACTCATAGTGATCATTTATCAAAAAATGACTCTGGTTATCAAATGATTGAACAACCGGGAGCAATTTACTTAAGATACTTAGTTGACATTCATAAAAAGTAT
                             Q  I  L  L  S  L  T  H  S  D  H  L  S  K  N  D  S  G  Y  Q  M  I  E  Q  P  G  A  I  Y  L  R  Y  L  V  D  I  H  K  K  Y
Lathraea squamaria          ........................................................................................................................
                             Q  I  L  L  S  L  T  H  S  D  H  L  S  K  N  D  S  G  Y  Q  M  I  E  Q  P  G  A  I  Y  L  R  Y  L  V  D  I  H  K  K  Y
Bartsia inaequalis          .................................................................................................................C......
                             Q  I  L  L  S  L  T  H  S  D  H  L  S  K  N  D  S  G  Y  Q  M  I  E  Q  P  G  A  I  Y  L  R  Y  L  V  D  I  H  N  K  Y

                                      4690      4700      4710      4720      4730      4740      4750      4760      4770      4780      4790      4800
                            ....|....|....|....|....|....|....|....|....|....|....|....|....|....|....|....|....|....|....|....|....|....|....|....|
L.squamaria minor sequence  CTATTGAATTATGAGTTCAATACATCCTGTTTAGCAGAAAGACGGGTATTCCTTGCTCATTATCAGACAATCACTTATTCACAAACTTCGTGTGGGACTAATACTTTGCATTTCCCATCT
                             L  L  N  Y  E  F  N  T  S  C  L  A  E  R  R  V  F  L  A  H  Y  Q  T  I  T  Y  S  Q  T  S  C  G  T  N  T  L  H  F  P  S
Lathraea squamaria          ........................................................................................................................
                             L  L  N  Y  E  F  N  T  S  C  L  A  E  R  R  V  F  L  A  H  Y  Q  T  I  T  Y  S  Q  T  S  C  G  T  N  T  L  H  F  P  S
Bartsia inaequalis          ........................................................................................................................
                             L  L  N  Y  E  F  N  T  S  C  L  A  E  R  R  V  F  L  A  H  Y  Q  T  I  T  Y  S  Q  T  S  C  G  T  N  T  L  H  F  P  S

                                      4810      4820      4830      4840      4850      4860      4870      4880      4890      4900      4910      4920
                            ....|....|....|....|....|....|....|....|....|....|....|....|....|....|....|....|....|....|....|....|....|....|....|....|
L.squamaria minor sequence  CAcGGAAAACCTTTTTCGCTCCGCTTAGCCTTATCCCCCTCTAGGGGGATTTTcGTGATAGGTTCTATAGGAACTGGACGATCCTATTTGGTCAAATACCTAGCGACAAACTCCTATGTT
                             H  G  K  P  F  S  L  R  L  A  L  S  P  S  R  G  I  F  V  I  G  S  I  G  T  G  R  S  Y  L  V  K  Y  L  A  T  N  S  Y  V
Lathraea squamaria          ..T..................................................A..................................................................
                             H  G  K  P  F  S  L  R  L  A  L  S  P  S  R  G  I  L  V  I  G  S  I  G  T  G  R  S  Y  L  V  K  Y  L  A  T  N  S  Y  V
Bartsia inaequalis          ..T..................................................A..................................................................
                             H  G  K  P  F  S  L  R  L  A  L  S  P  S  R  G  I  L  V  I  G  S  I  G  T  G  R  S  Y  L  V  K  Y  L  A  T  N  S  Y  V

                                      4930      4940      4950      4960      4970      4980      4990      5000      5010      5020      5030      5040
                            ....|....|....|....|....|....|....|....|....|....|....|....|....|....|....|....|....|....|....|....|....|....|....|....|
L.squamaria minor sequence  CCTTTCATTACGGTATTTCTGAACAAGTTCCTGGATAACAAGCCTAAAGGTTTTCTTTTTGATAATATCGATATTGATGGTAGTGACGATATTGATGAT~~~~~~~~~AGTGACAATATT
                             P  F  I  T  V  F  L  N  K  F  L  D  N  K  P  K  G  F  L  F  D  N  I  D  I  D  G  S  D  D  I  D  D           S  D  N  I
Lathraea squamaria          ...................................................................................................~~~~~~~~~............
                             P  F  I  T  V  F  L  N  K  F  L  D  N  K  P  K  G  F  L  F  D  N  I  D  I  D  G  S  D  D  I  D  D           S  D  N  I
Bartsia inaequalis          ...................................................................................................ATTGATGAT............
                             P  F  I  T  V  F  L  N  K  F  L  D  N  K  P  K  G  F  L  F  D  N  I  D  I  D  G  S  D  D  I  D  D  I  D  D  S  D  N  I

                                      5050      5060      5070      5080      5090      5100      5110      5120      5130      5140      5150      5160
                            ....|....|....|....|....|....|....|....|....|....|....|....|....|....|....|....|....|....|....|....|....|....|....|....|
L.squamaria minor sequence  GATGCTAGTGACGATATCGAtCGTGACCTTGATACGGAGCTGGAACTGCTAACTATGATGAATGCGCTAACTATGGATATGATGTCGGGAATAGGCCGATTTTATATCACCCTTCAATTC
                             D  A  S  D  D  I  D  R  D  L  D  T  E  L  E  L  L  T  M  M  N  A  L  T  M  D  M  M  S  G  I  G  R  F  Y  I  T  L  Q  F
Lathraea squamaria          ....................C...................................................................................................
                             D  A  S  D  D  I  D  R  D  L  D  T  E  L  E  L  L  T  M  M  N  A  L  T  M  D  M  M  S  G  I  G  R  F  Y  I  T  L  Q  F
Bartsia inaequalis          .......T...T............................................................................................................
                             D  A  I  D  D  I  D  R  D  L  D  T  E  L  E  L  L  T  M  M  N  A  L  T  M  D  M  M  S  G  I  G  R  F  Y  I  T  L  Q  F

                                      5170      5180      5190      5200      5210      5220      5230      5240      5250      5260      5270      5280
                            ....|....|....|....|....|....|....|....|....|....|....|....|....|....|....|....|....|....|....|....|....|....|....|....|
L.squamaria minor sequence  GAATTAGCAAAAGCAATGTCTCCTTGCATAATATGGATTCCAAACATCCATGATCTGGATGTGAATGAGTCgAATTACTTATCCTTCGGTCTATTAGTGAACCATCTCTCTGAAAGATGT
                             E  L  A  K  A  M  S  P  C  I  I  W  I  P  N  I  H  D  L  D  V  N  E  S  N  Y  L  S  F  G  L  L  V  N  H  L  S  E  R  C
Lathraea squamaria          .......................................................................A................................................
                             E  L  A  K  A  M  S  P  C  I  I  W  I  P  N  I  H  D  L  D  V  N  E  S  N  Y  L  S  F  G  L  L  V  N  H  L  S  E  R  C
Bartsia inaequalis          ...............................................T........................................................................
                             E  L  A  K  A  M  S  P  C  I  I  W  I  P  N  I  H  D  L  D  V  N  E  S  N  Y  L  S  F  G  L  L  V  N  H  L  S  E  R  C

                                      5290      5300      5310      5320      5330      5340      5350      5360      5370      5380      5390      5400
                            ....|....|....|....|....|....|....|....|....|....|....|....|....|....|....|....|....|....|....|....|....|....|....|....|
L.squamaria minor sequence  TCCACTAGAAATATTCTTGTTATTGCTTCGACTCATATTCCCCAAAAAGTGGATCCCGCTCTAATAGCTCCGAATAAATTAAATACGTGCATTAAGATACGAAGGCTTCTTATTCCACAA
```

```
                             S  T  R  N  I  L  V  I  A  S  T  H  I  P  Q  K  V  D  P  A  L  I  A  P  N  K  L  N  T  C  I  K  I  R  R  L  L  I  P  Q
Lathraea squamaria          ........................................................................................................................
                             S  T  R  N  I  L  V  I  A  S  T  H  I  P  Q  K  V  D  P  A  L  I  A  P  N  K  L  N  T  C  I  K  I  R  R  L  L  I  P  Q
Bartsia inaequalis          ........................................................................................................................
                             S  T  R  N  I  L  V  I  A  S  T  H  I  P  Q  K  V  D  P  A  L  I  A  P  N  K  L  N  T  C  I  K  I  R  R  L  L  I  P  Q

                                     5410      5420      5430      5440      5450      5460      5470      5480      5490      5500      5510      5520
                            ....|....|....|....|....|....|....|....|....|....|....|....|....|....|....|....|....|....|....|....|....|....|....|....|
L.squamaria minor sequence  CAACGAAAGCACTTTTTCACTCTTTCATATACTAGGGGATTTCACTTGGAAAAGAAAATGTTCCATACTAACGGATTCGGTTCCATAACCATGGGTTCCAATGCAAGAGATCTTGTAGCA
                             Q  R  K  H  F  F  T  L  S  Y  T  R  G  F  H  L  E  K  K  M  F  H  T  N  G  F  G  S  I  T  M  G  S  N  A  R  D  L  V  A
Lathraea squamaria          ........................................................................................................................
                             Q  R  K  H  F  F  T  L  S  Y  T  R  G  F  H  L  E  K  K  M  F  H  T  N  G  F  G  S  I  T  M  G  S  N  A  R  D  L  V  A
Bartsia inaequalis          ........................................................................................................................
                             Q  R  K  H  F  F  T  L  S  Y  T  R  G  F  H  L  E  K  K  M  F  H  T  N  G  F  G  S  I  T  M  G  S  N  A  R  D  L  V  A

                                     5530      5540      5550      5560      5570      5580      5590      5600      5610      5620      5630      5640
                            ....|....|....|....|....|....|....|....|....|....|....|....|....|....|....|....|....|....|....|....|....|....|....|....|
L.squamaria minor sequence  CTTACCAATGAGGCCCTATCGATTAGTATTACACAGAAgAAATCAATTCTAGACACTAATACAATTAGATCCGCTCTTCATAGACAAACTTGGGATTTGCGATCCCAGGTAAGATCGGTT
                             L  T  N  E  A  L  S  I  S  I  T  Q  K  K  S  I  L  D  T  N  T  I  R  S  A  L  H  R  Q  T  W  D  L  R  S  Q  V  R  S  V
Lathraea squamaria          ......................................A.................................................................................
                             L  T  N  E  A  L  S  I  S  I  T  Q  K  K  S  I  L  D  T  N  T  I  R  S  A  L  H  R  Q  T  W  D  L  R  S  Q  V  R  S  V
Bartsia inaequalis          ........................................................................................................................
                             L  T  N  E  A  L  S  I  S  I  T  Q  K  K  S  I  L  D  T  N  T  I  R  S  A  L  H  R  Q  T  W  D  L  R  S  Q  V  R  S  V

                                     5650      5660      5670      5680      5690      5700      5710      5720      5730      5740      5750      5760
                            ....|....|....|....|....|....|....|....|....|....|....|....|....|....|....|....|....|....|....|....|....|....|....|....|
L.squamaria minor sequence  CAGGATCATGGGATCCTTTCCTATCAGATAGGAcGGGCTGTAGCACAAAATGTACTTCTAAGTAATTGCCCCATAGATCCTATATCTATCTATATGAAGAAGAAATCATGTAACGAAGGG
                             Q  D  H  G  I  L  S  Y  Q  I  G  R  A  V  A  Q  N  V  L  L  S  N  C  P  I  D  P  I  S  I  Y  M  K  K  K  S  C  N  E  G
Lathraea squamaria          .................................A......................................................................................
                             Q  D  H  G  I  L  S  Y  Q  I  G  R  A  V  A  Q  N  V  L  L  S  N  C  P  I  D  P  I  S  I  Y  M  K  K  K  S  C  N  E  G
Bartsia inaequalis          .................................A......................................................................................
                             Q  D  H  G  I  L  S  Y  Q  I  G  R  A  V  A  Q  N  V  L  L  S  N  C  P  I  D  P  I  S  I  Y  M  K  K  K  S  C  N  E  G

                                     5770      5780      5790      5800      5810      5820      5830      5840      5850      5860      5870      5880
                            ....|....|....|....|....|....|....|....|....|....|....|....|....|....|....|....|....|....|....|....|....|....|....|....|
L.squamaria minor sequence  GATTCTTATTTGTACAAATGGTACTTCGAACTTGGAACGAGCATGAAGAAATTAACGATACTTCTTTATCTTTTAAGTTGTTCTGCCGGATCGGTCGCTCAAGATCTTTGGTCTCTACCC
                             D  S  Y  L  Y  K  W  Y  F  E  L  G  T  S  M  K  K  L  T  I  L  L  Y  L  L  S  C  S  A  G  S  V  A  Q  D  L  W  S  L  P
Lathraea squamaria          ........................................................................................................................
                             D  S  Y  L  Y  K  W  Y  F  E  L  G  T  S  M  K  K  L  T  I  L  L  Y  L  L  S  C  S  A  G  S  V  A  Q  D  L  W  S  L  P
Bartsia inaequalis          ........................................................................................................................
                             D  S  Y  L  Y  K  W  Y  F  E  L  G  T  S  M  K  K  L  T  I  L  L  Y  L  L  S  C  S  A  G  S  V  A  Q  D  L  W  S  L  P

                                     5890      5900      5910      5920      5930      5940      5950      5960      5970      5980      5990      6000
                            ....|....|....|....|....|....|....|....|....|....|....|....|....|....|....|....|....|....|....|....|....|....|....|....|
L.squamaria minor sequence  GGACCCGATGAAAAAAATGGGATCACTTCTTATGGACTCGTTGAGAATGATTCGGATCTAGTTCATGGCCTATTAGAAGTAGAAGGCGCTCTGGTGAGATCTTCACGGACAGAAAAAGAT
                             G  P  D  E  K  N  G  I  T  S  Y  G  L  V  E  N  D  S  D  L  V  H  G  L  L  E  V  E  G  A  L  V  R  S  S  R  T  E  K  D
Lathraea squamaria          ........................................................................................................................
                             G  P  D  E  K  N  G  I  T  S  Y  G  L  V  E  N  D  S  D  L  V  H  G  L  L  E  V  E  G  A  L  V  R  S  S  R  T  E  K  D
Bartsia inaequalis          ......................................T.................................................................................
                             G  P  D  E  K  N  G  I  T  S  Y  G  L  V  E  N  D  S  D  L  V  H  G  L  L  E  V  E  G  A  L  V  R  S  S  R  T  E  K  D

                                     6010      6020      6030      6040      6050      6060      6070      6080      6090      6100      6110      6120
                            ....|....|....|....|....|....|....|....|....|....|....|....|....|....|....|....|....|....|....|....|....|....|....|....|
L.squamaria minor sequence  TGCAGTCCGTTTGAGAATGATCGAGTTACATTGCTTCTTCGGACCGAACCGAGGAATCCCTTAGATATGATGCAAAATGGATCTTGTTCTATCTTTGATCAGAGATTTCTCTATGAAAAA
                             C  S  P  F  E  N  D  R  V  T  L  L  L  R  T  E  P  R  N  P  L  D  M  M  Q  N  G  S  C  S  I  F  D  Q  R  F  L  Y  E  K
Lathraea squamaria          ........................................................................................................................
                             C  S  P  F  E  N  D  R  V  T  L  L  L  R  T  E  P  R  N  P  L  D  M  M  Q  N  G  S  C  S  I  F  D  Q  R  F  L  Y  E  K
Bartsia inaequalis          ..........................................C.............................................................................
```

```
                                 C   S   P   F   E   N   D   R   V   T   L   L   L   R   P   E   P   R   N   P   L   D   M   M   Q   N   G   S   C   S   I   F   D   Q   R   F   L   Y   E   K

                                          6130      6140      6150      6160      6170      6180      6190      6200      6210      6220      6230      6240
                                 ....|....|....|....|....|....|....|....|....|....|....|....|....|....|....|....|....|....|....|....|....|....|....|....|
L.squamaria minor sequence       TACGAATCGGAGTTTGAAGAAGGGGAGGGGGAAGGAGCCCTTGACCCGCAACAGATCGAGGAGGATTTATTCAATCACATAGTTTGGGCTCCTAGAATATGGCGCCCTTGGGGCTTTCTA
                                  Y  E  S  E  F  E  E  G  E  G  E  G  A  L  D  P  Q  Q  I  E  E  D  L  F  N  H  I  V  W  A  P  R  I  W  R  P  W  G  F  L
Lathraea squamaria               ........................................................................................................................
                                  Y  E  S  E  F  E  E  G  E  G  E  G  A  L  D  P  Q  Q  I  E  E  D  L  F  N  H  I  V  W  A  P  R  I  W  R  P  W  G  F  L
Bartsia inaequalis               ......................................T.................................................................................
                                  Y  E  S  E  F  E  E  G  E  G  E  G  A  L  D  P  Q  Q  I  E  E  D  L  F  N  H  I  V  W  A  P  R  I  W  R  P  W  G  F  L

                                          6250      6260      6270      6280      6290      6300      6310      6320      6330      6340      6350      6360
                                 ....|....|....|....|....|....|....|....|....|....|....|....|....|....|....|....|....|....|....|....|....|....|....|....|
L.squamaria minor sequence       TTTGATGATTGTATCGAAAGGCCCAATTCATTGGGATTTCCCTATGGGTCCAGGTCATTTCGGGGTAAGAGGATCATTTACGATGAAGGGTATGAGCTTCAAGAGAATGATTCGGAGTTC
                                  F  D  D  C  I  E  R  P  N  S  L  G  F  P  Y  G  S  R  S  F  R  G  K  R  I  I  Y  D  E  G  Y  E  L  Q  E  N  D  S  E  F
Lathraea squamaria               ........................................................................................................................
                                  F  D  D  C  I  E  R  P  N  S  L  G  F  P  Y  G  S  R  S  F  R  G  K  R  I  I  Y  D  E  G  Y  E  L  Q  E  N  D  S  E  F
Bartsia inaequalis               ..................~~~~~~...GA....................................C........................G........G......C.............
                                  F  D  D  C  I  E        N  E  L  G  F  P  Y  G  S  R  S  F  R  G  K  R  I  I  Y  D  E  G  D  E  L  E  E  T  D  S  E  F

                                          6370      6380      6390      6400      6410      6420      6430      6440      6450      6460      6470      6480
                                 ....|....|....|....|....|....|....|....|....|....|....|....|....|....|....|....|....|....|....|....|....|....|....|....|
L.squamaria minor sequence       TTGCAGAGTGGAACCATGCAGTACCAGACACGAGATAGATCTTCCAAAGAACAAGGCCTTTTTCGAATAAGCCAATTCATTTGGGACCCTGCAGATCCGCTCTTTTTCCTATTCAAAGAT
                                  L  Q  S  G  T  M  Q  Y  Q  T  R  D  R  S  S  K  E  Q  G  L  F  R  I  S  Q  F  I  W  D  P  A  D  P  L  F  F  L  F  K  D
Lathraea squamaria               ........................................................................................................................
                                  L  Q  S  G  T  M  Q  Y  Q  T  R  D  R  S  S  K  E  Q  G  L  F  R  I  S  Q  F  I  W  D  P  A  D  P  L  F  F  L  F  K  D
Bartsia inaequalis               ........................................................................................................................
                                  L  Q  S  G  T  M  Q  Y  Q  T  R  D  R  S  S  K  E  Q  G  L  F  R  I  S  Q  F  I  W  D  P  A  D  P  L  F  F  L  F  K  D

                                          6490      6500      6510      6520      6530      6540      6550      6560      6570      6580      6590      6600
                                 ....|....|....|....|....|....|....|....|....|....|....|....|....|....|....|....|....|....|....|....|....|....|....|....|
L.squamaria minor sequence       CAGCCCCCAGGCTCTGTGTTTTCACATCGAGAATTATTTGCAGATGAAGAGATGTCAAAGGGGCTTCTTACTTCCCAAACAGATCCTCCTACATCTATATATAAACGCTGGTTTATCAAG
                                  Q  P  P  G  S  V  F  S  H  R  E  L  F  A  D  E  E  M  S  K  G  L  L  T  S  Q  T  D  P  P  T  S  I  Y  K  R  W  F  I  K
Lathraea squamaria               ........................................................................................................................
                                  Q  P  P  G  S  V  F  S  H  R  E  L  F  A  D  E  E  M  S  K  G  L  L  T  S  Q  T  D  P  P  T  S  I  Y  K  R  W  F  I  K
Bartsia inaequalis               ........................................................................................................................
                                  Q  P  P  G  S  V  F  S  H  R  E  L  F  A  D  E  E  M  S  K  G  L  L  T  S  Q  T  D  P  P  T  S  I  Y  K  R  W  F  I  K

                                          6610      6620      6630      6640      6650      6660      6670      6680      6690      6700      6710      6720
                                 ....|....|....|....|....|....|....|....|....|....|....|....|....|....|....|....|....|....|....|....|....|....|....|....|
L.squamaria minor sequence       AATACACAAGAAAAGCACTTCGAGTTGTTGATTAATCGTCAGAGATGGCTT~~~~~~~~~AGTTCATTATCTAATGGATCTTTCCGTTCTAATACTCTATCCGAGAGTTATCAGTATTTA
                                  N  T  Q  E  K  H  F  E  L  L  I  N  R  Q  R  W  L           S  S  L  S  N  G  S  F  R  S  N  T  L  S  E  S  Y  Q  Y  L
Lathraea squamaria               ...................................................~~~~~~~~~............................................................
                                  N  T  Q  E  K  H  F  E  L  L  I  N  R  Q  R  W  L           S  S  L  S  N  G  S  F  R  S  N  T  L  S  E  S  Y  Q  Y  L
Bartsia inaequalis               ...................................................AGAACCAAT............................................................
                                  N  T  Q  E  K  H  F  E  L  L  I  N  R  Q  R  W  L  R  T  N  S  S  L  S  N  G  S  F  R  S  N  T  L  S  E  S  Y  Q  Y  L

                                          6730      6740      6750      6760      6770      6780      6790      6800      6810      6820
                                 ....|....|....|....|....|....|....|....|....|....|....|....|....|....|....|....|....|....|....|....|..
L.squamaria minor sequence       TCAACTCTGTTCCTATCTAACGGAACGCTATTGGATCAAATGACAAAGACATTGTTAAGAAAAAGATGGCTTTTCCCGGATGAAATGCAAATTGGATTCATG
                                  S  T  L  F  L  S  N  G  T  L  L  D  Q  M  T  K  T  L  L  R  K  R  W  L  F  P  D  E  M  Q  I  G  F  M
Lathraea squamaria               ......................................................................................................
                                  S  T  L  F  L  S  N  G  T  L  L  D  Q  M  T  K  T  L  L  R  K  R  W  L  F  P  D  E  M  Q  I  G  F  M
Bartsia inaequalis               ......................................................................................................
                                  S  T  L  F  L  S  N  G  T  L  L  D  Q  M  T  K  T  L  L  R  K  R  W  L  F  P  D  E  M  Q  I  G  F  M
```
