# Supplementary material for: Complete Plastid Genome of the Recent Holoparasite Lathraea squamaria Reveals Earliest Stages of Plastome Reduction in Orobanchaceae
Source: PLoS One. 2016 Mar 2;11(3):e0150718. doi: 10.1371/journal.pone.0150718 (PMC4775063; doi:10.1371/journal.pone.0150718)

S1 Figure. Analysis of rpoC1, rpoC2, and rbcL cDNAs. a: Electrophoresis of results of cDNA amplification showing transcription and/or splicing of plastid genes (lanes 1-3 – rpoC1, fruits; 4-6 – rpoC1, perianth; 7-11 – rpoC2; 12-17 – rbcL). b: Chromatogram showing polymorphism in rbcL cDNA and DNA (T/C, marked by asterisks).

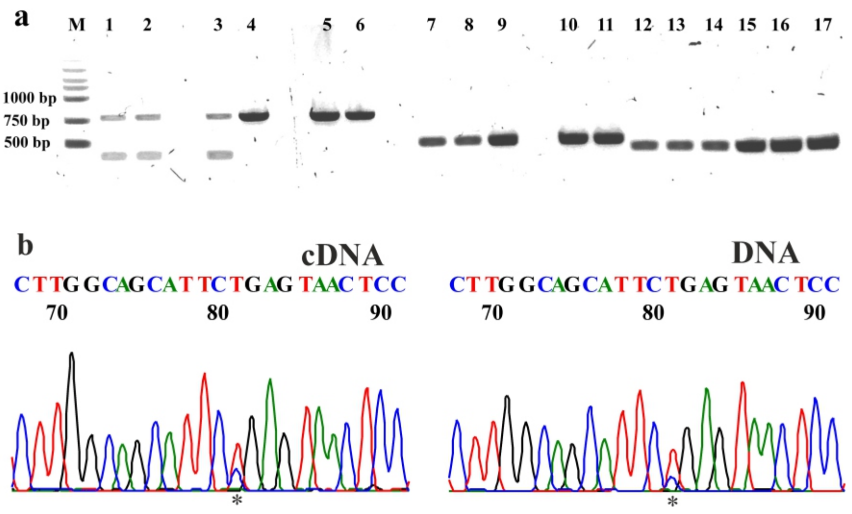

Supplement: S1 Fig — (PDF) [file pone.0150718.s002.pdf]
