# Supplementary material for: Complete Plastid Genome of the Recent Holoparasite Lathraea squamaria Reveals Earliest Stages of Plastome Reduction in Orobanchaceae
Source: PLoS One. 2016 Mar 2;11(3):e0150718. doi: 10.1371/journal.pone.0150718 (PMC4775063; doi:10.1371/journal.pone.0150718)
Supplement: S2 Fig — (PDF) [file pone.0150718.s003.pdf]

S2 Figure. Estimated ages in million years and 95% highest posterior density (HPD) intervals

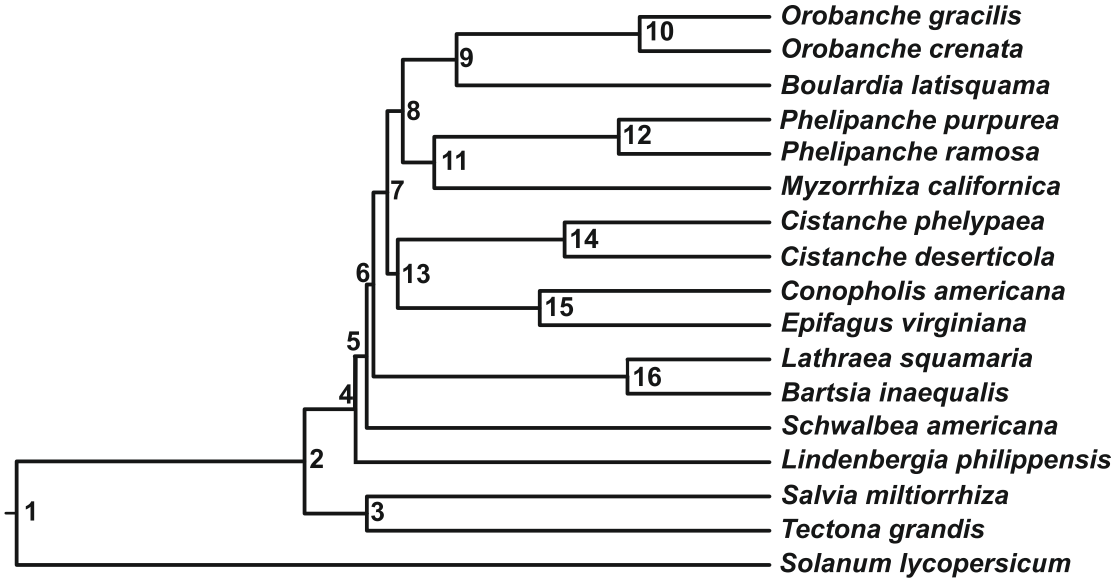

node	estimated age	95% HPD
1	96.69	94.89 - 99.75
2	45.46	43.10 - 50.21
3	39.37	31.67 - 45.12
4	40.46	37.67 - 44.79
5	39.38	36.54 - 43.58
6	38.72	35.86 - 42.87
7	37.36	34.45 - 41.47
8	35.85	32.85 - 39.90
9	30.59	26.45 - 34.90
10	12.69	08.92 - 17.21
11	32.77	29.33 - 36.84
12	14.73	10.80 - 19.14
13	36.34	33.29 - 40.43
14	20.03	13.70 - 26.55
15	22.45	16.99 - 28.04
16	13.89	08.17 - 21.59
