# Supplementary material for: Complete Plastid Genome of the Recent Holoparasite Lathraea squamaria Reveals Earliest Stages of Plastome Reduction in Orobanchaceae
Source: PLoS One. 2016 Mar 2;11(3):e0150718. doi: 10.1371/journal.pone.0150718 (PMC4775063; doi:10.1371/journal.pone.0150718)
Supplement: S1 Table — (PDF) [file pone.0150718.s004.pdf]

S1 Table. Primers used for plastome assembly and cDNA amplification.

Name	Sequence	Localization	Purpose
Lsqua-C11-F	AGTAGGATTCGACAATGGGTTTGA	20808-20831	PCR
Lsqua-C28-R	TGTTGGGACTGGGTTAGAACGACA	25292-25315	PCR
Lsqua-C28-F	ATTTCTCTTTGCCAAGGAGAAGATGC	35600-35625	PCR
Lsqua-C14-R	CAATTATGAAGCGTGGCTAAGTGAT	40496-40520	PCR
Lsqua-14-F	CGATAGCACCTTGATCAGTTAATTCAA	53597-53623	PCR
Lsqua-C5-R	GAACTCCCAATTCTCTAGCAAAT	55051-55073	PCR
Lsqua-C5-F	CCTCCATACTGAGATATACCATAAGAC	69210-69236	PCR
Lsqua-C23-R	ACAGCAACCCTAGTCGCCATAT	72473-72494	PCR
Lsqua-C25-F	GGGAGATATACTATTTCACCAATCG	87390-87414	PCR
Lsqua-C18-R	CATCTCGATCGGAAAAGAATCAAGAG	94690-94715	PCR
Lsqua-C18-F	TGTAGTGACGAATCTTGTATGTGTTC	97283-97308	PCR
Lsqua-C16-R	GAACGCTCCCCTACCGATGTAT	103008-103029	PCR
Lsqua-C16-F	GGATACTAATTGGCAAGAATTTGAAC	108013-108038	PCR
Lsqua-C7-R	GGACAAAATTCTAAACGCCTTA	108394-108415	PCR
Lat3-intern-R	TCAATTTATCTCTCTCAACTTGGA	54750-54773	sequencing
Lat4-intern-F	TTGGTTCTACCATATTTTTCATTTTATCC	70210-70238	sequencing
Lat4-intern1-F	CTAGAACCACCTAAAGTTCCAACTAA	72269-72294	sequencing
Lat6-intern-R	CGTTAGCTACAGCACTGCAC	98686-98705	sequencing
rbcL:54293U21	CCACAAACAGAGACTAAAGCA	54313-54333	cDNA amplification
rbcL:54646L22	GAATCCAAATACGTTTCCTACA	54666-54687	cDNA amplification
rpoC2:17330U21	TTGAACGCACTTCTAACACCT	17350-17370	cDNA amplification
rpoC2:17789L22	TACCGTTTAATCACTCATAACC	17809-17830	cDNA amplification
rpoC1:22354U21	GGATAAATCTAGATCGGCTAA	22374-22394	cDNA amplification
rpoC1:23444L20	GAAGATATCAGATGGGCTAC	23464-23483	cDNA amplification
Lsqua-LSC-IRa-F	AATTGTATGGCCGATCATTGTG	81864-81885	PCR
Lsqua- LSC-IRa-R	GTTATCCTGCACTCGGAAGAA	150329-150349	PCR
Lsqua-IRa-SSC-F	ACTGAGTAAACTTGTTCAAGAATCG	108100-108124	PCR
Lsqua- IRa-SSC-R	GTTGGCTCAATTAACGGAGTTT	108288-108309	PCR
Lsqua- SSC-IRb-F	CTCCCAATTGTTCTAACTCCGTT	124154-124179	PCR
Lsqua-IRb-LSC-R	TATATAAATCATCGTAACGAAGATGAT	141-167	PCR
Lath:63862U22F	CAAATATATCATAAGCTAAACC	63862-63884	PCR
Lath:64410L21	AATTAAACCTAACACGATTCC	64410-64431	PCR
