# Supplementary material for: Complete Plastid Genome of the Recent Holoparasite Lathraea squamaria Reveals Earliest Stages of Plastome Reduction in Orobanchaceae
Source: PLoS One. 2016 Mar 2;11(3):e0150718. doi: 10.1371/journal.pone.0150718 (PMC4775063; doi:10.1371/journal.pone.0150718)
Supplement: S2 Table — (PDF) [file pone.0150718.s005.pdf]

S2 Table. Genes encoded in the *Lathraea squamaria* and *Lindenbergia philippensis* plastomes. [a] containing one intron, [b] containing two introns, [c] genes in the IR regions (i.e., duplicated), *pseudogenes

Functional category	Group of genes	*Lathraea squamaria*	*Lindenbergia philippensis*
Protein synthesis and DNA-replication	rRNA genes	rrn16[c], rrn23[c], rrn4.5[c], rrn5[c]	rrn16[c], rrn23[c], rrn4.5[c], rrn5[c],
	tRNA genes	trnA-UGC[ac], trnC-GCA, trnD-GUC, trnE-UUC, trnH-GUG, trnF-GAA, trnG-GCC, trnG-UCC[a], trnI-GAU[ac], trnI-CAU[c], trnK-UUU[a], trnL-CAA[c], trnL-UAA[a], trnL-UAG, trnfM-CAU, trnM-CAU, trnN-GUU[c], trnP-UGG, trnQ-UUG, trnR-ACG[c], trnR-UCU, trnS-GCU, trnS-GGA, trnS-GCU, trnT-GGU, trnT-UGU, trnV-GAC[c], trnV-UAC, trnW-CCA, trnY-GUA	trnA-UGC[ac], trnC-GCA, trnD-GUC, trnE-UUC, trnH-GUG, trnF-GAA, trnG-GCC, trnG-UCC[a], trnI-GAU[ac], trnI-CAU[c], trnK-UUU[a], trnL-CAA[c], trnL-UAA[a], trnL-UAG, trnfM-CAU, trnM-CAU, trnN-GUU[c], trnP-UGG, trnQ-UUG, trnR-ACG[c], trnR-UCU, trnS-GCU, trnS-GGA, trnS-GCU, trnT-GGU, trnT-UGU, trnV-GAC[c], trnV-UAC, trnW-CCA, trnY-GUA
	Ribosomal small subunit	rps2, rps3, rps4, rps7[c], rps8, rps11, rps12_5’end, rps12_3’end[ac], rps14, rps15, rps16[a], rps18, rps19*	rps2, rps3, rps4, rps7[c], rps8, rps11, rps12_5’end, rps12_3’end[ac], rps14, rps15, rps16[a], rps18, rps19
	Ribosomal large subunit	rpl2[ac], rpl14, rpl16[a], rpl20, rpl22, rpl23*[c], rpl32, rpl33, rpl36	rpl2[ac], rpl14, rpl16[a], rpl20, rpl22, rpl23[c], rpl32, rpl33, rpl36
	DNA-dependent RNA polymerase	rpoA*, rpoB, rpoC1[a], rpoC2*	rpoA, rpoB, rpoC1[a], rpoC2
Photosynthesis	Large subunit of rubisco	rbcL*	rbcL
	Photosystem I	psaA*, psaB*, psaC, psaI, psaJ*	psaA, psaB, psaC, psaI, psaJ
	Photosystem II	psbA*, psbB*, psbC*, psbD*, psbE, psbF, psbH*, psbI*, psbJ*, psbK, psbL, psbM, psbN, psbT*, psbZ	psbA, psbB, psbC, psbD, psbE, psbF, psbH, psbI, psbJ, psbK, psbL, psbM, psbN, psbT, psbZ
	NADH	ndhA*, ndhB*[c], ndhC, ndhD*,	ndhA ndhJ, ndhK, ndhC,

	dehydrogenase	ndhE, ndhF*, ndhG*, ndhH*, ndhI*, ndhJ*, ndhK*	ndhB[ac], ndhF, ndhI, ndhH, ndhD ndhE, ndhG
	Cytochrome b/f complex	petA*, petB*, petD*, petG*, petN	petA, petB[a], petD[a], petG, petL, petN
	ATP synthase	atpA, atpB, atpE, atpF[a], atpH, atpI	atpA, atpB, atpE, atpF[a], atpH, atpI
Other	Maturase	matK	matK
	Subunit of acetyl-CoA carboxylase	accD	accD
	Membrane protein	cemA*	cemA
	Protease	clpP[b]	clpP[b]
	Translational initiation factor	infA	infA
	c-type cytochrome synthesis	ccsA*	ccsA
	Conserved open reading frames (ycf)	ycf1, ycf1*, ycf2[c], ycf3[b], ycf4*	ycf1, ycf1*, ycf2[c], ycf3[b], ycf4
